# Dithiocarbazate ligands and their Ni(II) complexes with potential biological activity: Structural, antitumor and molecular docking study

**DOI:** 10.3389/fmolb.2023.1146820

**Published:** 2023-03-07

**Authors:** Cássia de Q. O. Cavalcante, Tales H. A. da Mota, Diêgo M. de Oliveira, Érica C. M. Nascimento, João B. L. Martins, Fabio Pittella-Silva, Claudia C. Gatto

**Affiliations:** ^1^ University of Brasília, Institute of Chemistry, Laboratory of Inorganic Synthesis and Crystallography, Brasília, DF, Brazil; ^2^ University of Brasília, Faculdade UnB Ceilândia, Multidisciplinary Laboratory of Human Health, Brasília, DF, Brazil; ^3^ University of Brasília, Institute of Chemistry, Laboratory of Computational Chemistry, Brasília, DF, Brazil; ^4^ University of Brasília, Faculty of Health Sciences and Medicine, Laboratory of Molecular Cancer Pathology, Brasília, DF, Brazil

**Keywords:** Ni(II) complexes, dithiocarbazate, crystal structure, mass spectrometry, Hirshfeld surface, antitumor activity

## Abstract

In the search for new metal complexes with antitumor potential, two dithiocarbazate ligands derived from 1,1,1-trifluoro-2,4-pentanedione (H_2_L^1^) and (H_2_L^2^) and four Ni(II) complexes, [Ni(L^1^)PPh_3_] (1), [Ni(L^1^)Py] (2), [Ni(L^2^)PPh_3_] (3), and [Ni(L^2^)Py] (4), were successfully synthesized and investigated by physical-chemistry and spectroscopic methods. The crystal structure of the H_2_L^1^ and the Ni(II) complexes has been elucidated by single-crystal X-ray diffraction. The obtained structure from H_2_L^1^ confirms the cyclization reaction and formation of the pyrazoline derivative. The results showed square planar geometry to the metal centers, in which dithiocarbazates coordinated by the *ONS* donor system and a triphenylphosphine or pyridine molecule complete the coordination sphere. Hirshfeld surface analysis by *d*_*norm*_ function was investigated and showed π–π stacking interactions upon the molecular packing of H_2_L^1^ and non-classical hydrogen bonds for all compounds. Fingerprint plots showed the main interactions attributed to H⋅H C⋅H, O⋅H, Br⋅H, and F⋅H, with contacts contributing between 1.9% and 38.2%. The mass spectrometry data indicated the presence of molecular ions [M + H]^+^ and characteristic fragmentations of the compounds, which indicated the same behavior of the compounds in solution and solid state. Molecular docking simulations were studied to evaluate the properties and interactions of the free dithiocarbazates and their Ni(II) complexes with selected proteins and DNA. These results were supported by *in vitro* cytotoxicity assays against four cancer cell lines, showing that the synthesized metal complexes display promising biological activity.

## 1 Introduction

Despite the discovery in recent decades of new drugs and advances in the treatment of the most varied types of cancer, there are still many problems regarding the selectivity and side effects of chemotherapeutic agents, which motivates many groups of researchers to synthesize new compounds and test the potential cytotoxic activity against different cancer cell lines (Oun et al., 2018; Ambika et al., 2019; Raisa and Marhaendraputro, 2019; Matela, 2020; Mbugua et al., 2020; Parveen, 2020; Pellei et al., 2021; Yusof et al., 2021).

The Schiff bases are organic compounds widely studied due to their applications in bioinorganic chemistry. In this sense, dithiocarbazates stand out, which are ligands that show different biological activities (Nanjundan et al., 2017; Kongot et al., 2019; Lima et al., 2020; Samy and Omar, 2020; Ramilo-Gomes et al., 2021; Yusof et al., 2022), with emphasis on antitumor action. Recently, studies described copper and vanadium complexes with dithiocarbazates with interesting results and showing an IC_50_ value much better than that of cisplatin (Gou et al., 2021; Sahu et al., 2022). In addition to their biological actions, dithiocarbazates are also studied for their chemical and structural versatility. They have different donor atoms that can coordinate with metals and form stable chelates. These compounds can also be modified in their -R groups, leading to different possibilities and coordination sites, such as bi, tri, or polydentate forms, in addition to forming complexes with different geometries (Li et al., 2012; Lima et al., 2018; Takjoo et al., 2018; Yekke-Ghasemi et al., 2018; Yusof et al., 2019; Boshaala et al., 2021). Furthermore, the biological action of free dithiocarbazates can be potentiated or suppressed (protective effect) after complexing with different metal ions (Cavalcante et al., 2019; Sohtun et al., 2020).

Nickel has gained prominence in the field of bioinorganic chemistry (Elsayed et al., 2021; Lima et al., 2021), as can be seen in recently published works with nickel and dithiocarbazates. Some of these compounds were tested for their cytotoxicity against strains of breast, colon, and liver cancer cells and showed outstanding antitumor activity against breast cancer cells MCF7, inducing cell death by cellular apoptosis. Some studies show that the presence of halogens in the dithiocarbazate structures can considerably increase the biological activity (Chrzanowska et al., 2021; da Silva et al., 2022).

Considering our interest in developing metal complexes with antitumor potential (Lima et al., 2021; Almeida et al., 2022; Cavalcante et al., 2022), we describe the synthesis and characterization by single-crystal X-ray diffraction, Hirshfeld surface, FT-IR, UV–Vis, mass spectrometry, and ^1^H nuclear magnetic resonance of dithiocarbazate ligands derivated from 1,1,1-trifluoro-2,4-pentanedione and their Ni(II) complexes. In addition, the cytotoxic potential of all compounds against human cancer cell lines was evaluated, and the results were compared to indicate the impact of structural variations on biological activity (Gu et al., 2019; Wang et al., 2019; Cavalcante et al., 2022). Finally, the complexes were evaluated for molecular docking studies.

## 2 Materials and methods

### 2.1 Materials and instrumentation

The reagents and solvents used in this work were obtained from commercial sources (Sigma-Aldrich). ESI-MS and ESI-MSMS mass spectra were obtained using solutions with a concentration of 50 μM in methanol for the complexes [Ni(L^1^)Py] (2) and [Ni(L^2^)Py] (4) and in the ratio 99/1% (methanol/dimethylformamide) for all other compounds. The spectra were obtained in positive mode, using 0.1% acetic acid, and the equipment used was an AB Sciex Triple TOF 5600 + spectrometer. ^1^H Nuclear Magnetic Resonance (^1^H NMR) spectra were obtained on a BRUKER Avance III HD 14T spectrometer, and the samples were prepared with 0.5 mL of deuterated dimethylsulfoxide solvent (DMSO-d_6_) using tetramethylsilane as an internal reference. Fourier transform infrared spectra (FT-IR) were obtained using the Varian 640-IR FT-IR equipment in the range of 4,000–400 cm^−1^ and 4 cm^−1^ resolution. The samples were analyzed in the form of a solid pellet, containing a ratio of 1.0 mg of the compound to 100.0 mg of KBr. UV–Vis spectra were determined using solutions with concentrations of 2 × 10^−5^ mol.L^−1^ in two different solvents: methanol (MeOH) and dimethylsulfoxide (DMSO). The equipment used for the analysis was the VARIAN Cary 5,000 spectrophotometer.

### 2.2 Synthesis of H_2_L^1^

The synthesis was based on the literature (Ali et al., 2011; Cavalcante et al., 2022). A mixture of 314.9 mg (3 mmol) of hydrazine dihydrochloride and 359.9 mg (9 mmol) of NaOH in 30 mL of ethanol 95% and 5 mL of H_2_O was cooled down to 5°C and refluxed for 1 h. A quantity of 0.180 mL (3 mmol) of carbon disulfide was added slowly for 1 h. After that, at room temperature, 750 mg (3 mmol) of 1-bromo-4-(bromomethyl)benzene was added to the initial mixture previously dissolved in 10 mL of isopropyl alcohol, with constant stirring. After 1 h, 0.35 mL (3 mmol) of 1,1,1-trifluoro-2,4-pentanedione was added, and the final mixture was refluxed and heated for 4 h. Colorless crystals suitable for X-ray diffraction were obtained and filtered after slow evaporation of the solvent. Yield: 63% (780.5 mg). Melting point: 85°C. Calcd for C_13_H_12_BrF_3_N_2_OS_2_: C, 37.78; H, 2.93; N, 6.78; Found: C, 37.73; H, 2.69; N, 6.66. Selected IR bands (KBr, ν/cm^-1^): ν(C=S) 1,300, ν(C–S) 734, ν(O–H) 3,199, ν(N–N) 1,113, ν(C=N) 1,643, ν(C–F) 1,200–1,137, ν(C=C) 1,588 e 1486. RMN ^1^H (DMSO-d_6_ δ, ppm): 2.05 (s, 3H, C**H**_**3**_); 3.32 (d, ^*2*^ *J* = 19.81 Hz, H, C**H**_**2**_); 3.64 (d, ^*2*^ *J* = 19.81 Hz, H, C**H**_**2**_); 4.30 (d, ^*2*^ *J* = 13.94 Hz, H, S-C**H**_**2**_); 4.35 (d, ^*2*^ *J* = 13.94 Hz, H, S-C**H**_**2**_); aromatic ring: 7.34 (d, ^*3*^ *J*_*ortho*_ = 8.44 Hz, 2H, –CH^9^_Ar_ =), 7.51 (d, ^*3*^ *J*_*ortho*_ = 8.44 Hz, 2H, –CH^10^_Ar_ =), 8.26 (s, H, O-H). UV–Vis (MeOH): λmax = 238 nm, 297 nm, and 389 nm. UV–Vis (DMSO): λmax = 258 nm, 302 nm, and 258 nm. ESI-MS [M + H]^+^ (calcd, found, m/z) = 412.9605/412.9595.

### 2.3 Synthesis of H_2_L^2^

For the synthesis of the H_2_L^2^ ligand, the same steps for the synthesis of the H_2_L^1^ ligand were performed with a variation in the third step, where instead of adding 1-bromo-4-(bromomethyl)benzene, 648 mg (3 mmol) of 4-nitrobenzyl bromide was added previously dissolved in 10 mL of isopropyl alcohol. A yellow solid was obtained, which was filtered and allowed to dry at room temperature. Yield: 89% (1,009.2 mg). Melting point: 87°C. Calcd for C_13_H_12_F_3_N_3_O_3_S_2_: C, 41.15; H, 3.19; N, 11.07; Found: C, 40.86; H, 2.89; N, 11.56. Selected IR bands (KBr, ν/cm^−1^): ν(C=S) 1,310, ν(C–S) 732, ν(O–H) 3,151, ν(N–N) 1,113, ν(C=N) 1,636, ν(C–F) 1,205–1,138, ν(C=C) 1,596 e 1446, ν(NO_2_)_asy_ 1,515, ν(NO_2_)_sym_ 1,344. RMN ^1^H (DMSO-d_6_ δ, ppm): 2.06 (s, 3H, C**H**_**3**_); 3.32 (d, ^*2*^ *J* = 19.81 Hz, H, C**H**_**2**_); 3.65 (d, ^*2*^ *J* = 19.81 Hz, H, C**H**_**2**_); 4.49 (d, ^*2*^ *J* = 14.31 Hz, H, S-C**H**_**2**_); 4.55 (d, ^*2*^ *J* = 14.31 Hz, H, S-C**H**_**2**_); aromatic ring: 7.65 (d, ^*3*^ *J*_*ortho*_ = 8.80 Hz, 2H, –CH^9^_Ar_ =), 8.18 (d, ^*3*^ *J*_*ortho*_ = 8.80 Hz, 2H, –CH^10^_Ar_ =), 8.33 (s, H, O-H). UV–Vis (MeOH): λmax = 275 nm. UV–Vis (DMSO): λmax = 284 nm. ESI-MS [M + H]^+^ (calcd, found, m/z) = 380.0350/380.0348.

### 2.4 Synthesis of (1)

In the first step, 0.2 mmol (52.4 mg) of triphenylphosphine (PPh_3_) previously dissolved in 5 mL of MeOH and 0.1 mmol (23.7 mg) of NiCl_2_⋅6H_2_O in 5 mL of MeOH were refluxed for 20 min. After this, 0.1 mmol (41.3 mg) of H_2_L^1^ dissolved in 5 mL of methanol was added, and the reaction continued for another 1 h at reflux. A red precipitate was filtered and recrystallized from a mixture of 2 mL of dimethylformamide (DMF) and 2 mL of methanol. Red crystals suitable for single-crystal X-ray diffraction were obtained after slow evaporation of the solvents. Yield: 72% (52.3 mg). Melting point: Calcd for C_31_H_25_BrF_3_N_2_NiOPS_2_: C, 50.85; H, 3.44; N, 3.83; found: C, 50.07; H, 3.43; N, 4.41. 134°C. Selected IR bands (KBr, ν/cm^-1^): ν(C–S) 745, ν(N–N) 1,096, ν(C=N) 1,599, ν(C–F) 1,182–1,137, ν(C=C) 1,529 e 1483, ν(Ni-PPh_3_) 693. RMN ^1^H (DMSO-d_6_ δ, ppm): 2.41 (s, 3H, C**H**_**3**_); 4.28 (s, 2H, S-C**H**_**2**_); 5.89 (s, H, C**H**); aromatic: 7.31 (d, ^*3*^ *J*_*ortho*_ = 8.44 Hz, 2H, –CH^9^_Ar_ =), 7.44–7.33 (m, 17H, –CH^10^_Ar_ = and H of PPh_3_). UV–Vis (MeOH): λmax = 237 nm, 262 nm, 318 nm, and 369 nm. UV–Vis (DMSO): λmax = 265 nm and 380 nm. ESI-MS [M + H]^+^ (calcd, found, m/z) = 730.9713/730.9730.

### 2.5 Synthesis of (2)

An amount of 0.2 mmol (16 µL) of pyridine (Py) was added to a solution of 0.1 mmol (23.7 mg) of NiCl_2_⋅6H_2_O in 5 mL of MeOH and refluxed for 20 min. After this, 0.1 mmol (41.3 mg) of H_2_L^1^ dissolved in 5 mL of methanol was added, and the reaction continued for another 1 h at reflux. A red precipitate was filtered and recrystallized with 2 mL of DMF. Red crystals suitable for single-crystal X-ray diffraction were obtained after slow evaporation of the solvent. Yield: 65% (35.6 mg). Melting point: 142°C. Calcd for C_18_H_15_BrF_3_N_3_NiOS_2_: C, 39.38; H, 2.75; N, 7.65; Found: C, 39.33; H, 2.57; N, 7.55. Selected IR bands (KBr, ν/cm^−1^): ν(C–S) 760, ν(N–N) 1,070, ν(C=N) 1,606, ν(C–F) 1,192–1,114, ν(C=C) 1,528 e 1485, δ(Py) 689. RMN ^1^H (DMSO-d_6_ δ, ppm): 1.44 (s, 3H, C**H**_**3**_); 4.03 (s, 2H, S-C**H**_**2**_); 4.89 (s, H, C**H**); aromatic: 7.34 (d, ^*3*^ *J*_*ortho*_ = 8.07 Hz, 2H, –CH^9^_Ar_ =), 7.52 (d, ^*3*^ *J*_*ortho*_ = 8.07 Hz, 2H, –CH^10^_Ar_ =) 8.12–7.97 (m, 3H, H of Py), 10.46 (s, 2H, H of Py). UV–Vis (MeOH): λmax = 246 nm, 310 nm, and 372 nm. UV–Vis (DMSO): λmax = 265 nm and 381 nm. ESI-MS [M + H]^+^ (calcd, found, m/z) = 547.9224/547.9243.

### 2.6 Synthesis of (3)

An amount of 0.2 mmol (52.4 mg) of triphenylphosphine (PPh_3_) previously dissolved in 5 mL of MeOH and 0.1 mmol (23.7 mg) of NiCl_2_⋅6H_2_O in 5 mL of MeOH were refluxed for 20 min. After this, 0.1 mmol (37.9 mg) of H_2_L^2^ dissolved in 5 mL of methanol was added, and the reaction continued for another 1 h at reflux. A red precipitate was filtered and recrystallized from a mixture of 2 mL of DMF and 2 mL of MeOH. Red crystals suitable for single-crystal X-ray diffraction were obtained after slow evaporation of the solvents. Yield: 86% (60.0 mg). Melting point: 167°C. Calcd for C_31_H_25_F_3_N_3_NiO_3_PS_2_: C, 53.52; H, 3.61; N, 6.02; found: C, 53.56; H, 3.53; N, 6.17. Selected IR bands (KBr, ν/cm^-1^): ν(C–S) 747, ν(N–N) 1,096, ν(C=N) 1,598, ν(C–F) 1,179–1,139, ν(C=C) 1,479, ν(NO_2_)_asy_ 1,515, ν(NO_2_)_sym_ 1,342, ν(Ni-PPh_3_) 693. RMN ^1^H (DMSO-d_6_ δ, ppm): 2.39 (s, 3H, C**H**_**3**_); 4.43 (s, 2H, S-C**H**_**2**_); 5.58 (s, H, C**H**); aromatic: 7.44–7.73 (m, 17H, –CH^9^_Ar_ = and H of PPh_3_), 8.18 (d, ^*3*^ *J*_*ortho*_ = 8.44 Hz, 2H, –CH^10^_Ar_ = ). UV–Vis (MeOH): λmax = 241 nm, 268 nm, and 368 nm. UV–Vis (DMSO): λmax = 271 nm and 378 nm. ESI-MS [M + H]^+^ (calcd, found, m/z) = 698.0459/698.0463.

### 2.7 Synthesis of (4)

An amount of 0.2 mmol (16 µL) of pyridine (Py) was added to a solution of 0.1 mmol (23.7 mg) of NiCl_2_⋅6H_2_O in 5 mL of MeOH and refluxed for 20 min. After this, 0.1 mmol (37.9 mg) of H_2_L^2^ dissolved in 5 mL of methanol was added, and the reaction continued for another 1 h at reflux. A red precipitate was filtered and recrystallized with 2 mL of DMF. Red crystals suitable for single-crystal X-ray diffraction were obtained after slow evaporation of the solvent. Yield: 74% (38.3 mg). Melting point: 194°C. Calcd for C_18_H_15_F_3_N_4_NiO_3_S_2_: C, 41.97; H, 2.93; N, 10.88; found: C, 42.33; H, 2.80; N, 10.86. Selected IR bands (KBr, ν/cm^-1^): ν(C–S) 759, ν(N–N) 1,068, ν(C=N) 1,604, ν(C–F) 1,190–1,113, ν(C=C) 1,486, ν(NO_2_)_asy_ 1,516, ν(NO_2_)_sym_ 1,342, δ(Py) 689. RMN ^1^H (DMSO-d_6_ δ, ppm): 1.23 (s, 3H, C**H**_**3**_); 4.09 (s, 2H, S-C**H**_**2**_); 4.71 (s, H, C**H**); aromátics: 7.67 (d, ^*3*^ *J*_*ortho*_ = 8.44 Hz, 2H, –CH^9^_Ar_ = ), 8.13 (m, H, H of Py), 8.21 (d, ^*3*^ *J*_*ortho*_ = 8.44 Hz, 2H, –CH^10^_Ar_ = ) 8.27 (s, 2H, H of Py), 11.14 (s, 2H, H of Py). UV–Vis (MeOH): λmax = 264 nm and 371 nm. UV–Vis (DMSO): λmax = 270 nm and 380 nm. ESI-MS [M + H]^+^ (calcd, found, m/z) = 514.9969/514.9952.

### 2.8 Single-crystal X-ray structure determination

The equipment used for the X-ray diffraction was a Bruker CCD SMART APEX II (Charge Coupled Device Detector Bruker) diffractometer. This equipment has a graphite monochromator with an Mo–Kα radiation source (0.71073 Å), maintaining a collection temperature of approximately 296 K. SADABS (Sheldrick, 1997) was used for absorption correction. All structures were later solved using the Olex2 program (Dolomanov et al., 2009), with the SHELXS (Sheldrick, 2008) refinement option, and finished from option SHELXL (Sheldrick, 2015) with minimization of least squares. Data from the unit cells were obtained by collecting three matrices, each with twelve images. The images of the crystalline structures of the complexes and the representations of their unit cells were also generated in the program Olex2. The crystal data, experimental details, and refinement results are summarized in Table 1.

**TABLE 1:** X-ray diffraction data collection and refinement parameters for the ligand H_2_L^1^ and the complexes (1–4).

	H_2_L^1^	(1)	(2)	(3)	(4)
Empirical formula	C_13_H_12_BrF_3_N_2_OS_2_	C_31_H_25_BrF_3_N_2_NiOPS_2_	C_18_H_15_BrF_3_N_3_NiOS_2_	C_31_H_25_F_3_N_3_NiO_3_PS_2_	C_18_H_15_F_3_N_4_NiO_3_S_2_
Formula weight	413.28	732.24	549.07	698.34	515.17
Crystal system	Monoclinic	Orthorhombic	Monoclinic	Orthorhombic	Monoclinic
Space group	*P2*_*1*_ */c*	*P2*_*1*_ *2*_*1*_ *2*_*1*_	*P2*_*1*_ */n*	*P2*_*1*_ *2*_*1*_ *2*_*1*_	*P2*_*1*_ */n*
a (Å)	9.809 (2)	8.243 (13)	4.346 (3)	8.187 (5)	4.249 (6)
b (Å)	8.998 (2)	14.951 (2)	38.97 (3)	14.883 (9)	39.312 (5)
c (Å)	18.304 (4)	25.420 (5)	12.785 (9)	25.209 (15)	12.622 (17)
β (º)	91.068 (5)	90	96.935 (19)	90	98.778 (4)
V (Å^3^)	1,615.2 (6)	3,132.9 (9)	2,150 (3)	3,072 (3)	2083.6 (5)
Z	4	4	4	4	4
Density (mg·cm^−3^)	1.700	1.552	1.697	1.510	1.642
Index ranges	−11 ≤ h ≤ 11	−10 ≤ h ≤ 10	−5 ≤ h ≤ 5	−9 ≤ h ≤ 9	−5 ≤ h ≤ 5
	−10 ≤ k ≤ 10	−17 ≤ k ≤ 14	−46 ≤ k ≤ 46	−17 ≤ k ≤ 15	−47 ≤ k ≤ 47
	−22 ≤ l ≤ 21	−31 ≤ l ≤ 27	−15 ≤ l ≤ 15	−30 ≤ l ≤ 20	−15 ≤ l ≤ 15
Absorption coefficient (mm^−1^)	2.835	2.125	2.995	0.876	1.187
Absorption correction	Multi-scan	Multi-scan	Multi-scan	Multi-scan	Multi-scan
Reflections collected	20532	20689	26755	10270	25275
Independent reflections/Rint	2,973/0.104	5,858/0.158	3,886/0.171	5,627/0.085	3,772/0.054
Data/restraints/param	2,973/0/202	5,858/0/381	3,886/0/264	5,627/0/398	3,772/0/281
R_1_/wR_2_ [I > 2s(I)]	0.042/0.081	0.060/0.082	0.042/0.072	0.062/0.080	0.072/0.188
Goodness-of-fit on F^2^	1.013	0.937	0.752	0.896	1.172
Largest diff. peak and hole (eÅ^–3^)	0.41 and −0.43	0.46 and −0.48	0.47 and −0.57	0.37 and −0.33	0.93 and −0.52

### 2.9 Hirshfeld surface analysis

Crystallographic information files (CIFs) obtained from single-crystal X-ray diffraction were used as input files, and the Hirshfeld surfaces (HS) and related 2D-fingerprint plots (FP) were generated using the CrystalExplorer 17.5 program (Turner et al., 2017). The distances *d*_*i*_ and *d*_*e*_ correspond to the distance from the mapped surface to the nearest atom outside the surface and the distance from the mapped surface to the nearest atom inside the surface, respectively. The program CrystalExplorer correlates these two geometric functions and generates the *d*_*norm*_ surface, in which the distances are normalized by the van der Waals radii of the atoms involved. The generated *d*_*norm*_ surface allows mapping the regions involved in the intermolecular interactions important in the crystal formation through a color pattern that goes from blue (longer contacts) to red (closer contacts). The 3D *d*_*norm*_ surfaces were mapped over a fixed color scale of −0.2000 (red) to 1.4000 (blue). Fingerprint plots are *d*_*i*_ *versus d*_*e*_ 2D graphs and present a summary of the contacts present in the structures and are unique for each compound.

### 2.10 Biological activity

#### 2.10.1 Cell culture

The adherent cell lines MDA-MB-231 (breast cancer) and U251 (glioma) and the non-adherent cells NALM-6 and 697 (leukemia) composed an *in vitro* model for screening the cytotoxic activity of the compounds. The cells were cultured in plastic plates (adherent cells) or flakes (non-adherent cells) under controlled conditions (a humid atmosphere of 5% CO_2_ at 37°C) in Dulbecco’s modified Eagle’s medium (DMEM) supplemented with 10% (v/v) fetal bovine serum, penicillin (100 IU·mL^−1^) and streptomycin (100 mg mL^-1^), which was replaced every 2 to 3 days.

#### 2.10.2 Cell treatment and analysis of viability

For treatment, all compounds were diluted in dimethyl sulfoxide (DMSO) and underwent serial dilution. The concentrations used in the experiments ranged from 0.29 µM to 150 µM. The cells were seeded in 96-well plates, and the wells were split into groups (N = 8) and then exposed, separately, to the compounds at increasing concentrations. The final concentration of DMSO was 0.01% in all groups and the untreated controls. Cell viability was measured by the MTT reduction method for adherent cancer cells. This method is based on the ability of viable cells to metabolize yellow 3-(4,5-dimethyl-2-thiazolyl)-2,5-diphenyl-2H-tetrazolium bromide (MTT) through their mitochondrial dehydrogenases into a purple-stained formazan product, which is then measured by spectrophotometry. Briefly, the cells were plated at a density of 11.000 cells per well and treated with each compound. After 72 h, the culture medium was exchanged with a medium containing the MTT solution, and the plates were incubated for 2 hours. Cells are then lysed for spectrophotometric quantification at a wavelength of 595 nm. For the non-adherent leukemia cells, the viability after treatment with the compounds was measured using the resazurin reduction method. This assay is based on the ability of viable cells to metabolize resazurin. Briefly, the cells were plated at a density of 300.000 cells per well and treated with each compound. After 72 h, the fluorimetric resazurin reduction method was conducted according to the manufacturer’s instructions (CellTiter-Blue; Promega). In all cases, the results were expressed as a percentage of the control viability.

#### 2.10.3 Data analysis

Data were expressed as the mean SEM or median and ranges (according to the distribution) and submitted to analytical treatment. The statistical approach adopted for each analysis is described in the figure legends. Non-parametric tests were used for data with non-normal distribution. Probability values of *p* < 0.05 were accepted as an indication of a statistically significant difference. Non-linear regressions were used to demonstrate the dose–response patterns, and they were performed using the logarithmic equation models of the GraphPad Prism software library. *R* ^2^ values greater than 0.9 were adopted as satisfactory.

### 2.11 Molecular docking

The protein–ligand docking software GOLD (Jones et al., 1997), included in the computational molecular modeling package of the Cambridge Crystallographic Data Centre suits, was used to model the best pose and main interactions between the ligands H_2_L^1^ and H_2_L^2^ and four nickel complexes (1, 2, 3, and 4) with four different biological targets. The enzyme targets were established in the proteomic participant of the four different types of cells taken into account in the present study. The rank of the best fitness of binding energy was obtained using the ASP fitness function (Annamala et al., 2007), which brings the ChemScore metal terms in its score function.

For the NALM-6 cell type, the enzyme target chosen was the human deoxycytidine kinase (DCK), with the C4S-S74E mutant that is complexed with UDP (uridine-5′-diphosphate) and the DI-39 inhibitor molecules; the crystallographic structure of this protein presents a resolution of 2.09 Å, and it is deposited in the *RSCB Protein Data Bank* under the code **4KCG** (Nathanson et al., 2014). This enzyme is related to regulation of replication of lymphoblastic leukemia cells. The docking study for this receptor was carried out in the duplicate essay. One essay considers the active site related to the UDP ligand site, and the other essay was taken centering the grid box in the region of the DI-39 inhibitor to better understand how our ligands promote the inhibition of this enzyme.

To define a proper proteomic target for the 697 cell type to perform the docking study, the crystallographic structure chosen was the DNA binding protein double homeobox 4 fused with immunoglobulin domain 1 and 2 (DUX4_1-150_-DNA_ERG_ HD1–HD2) complexed with the expression recombinant gene (ERG_ALT_). The abnormal expression of this protein is related to the completed leukemogenesis in pediatric patients with acute lymphoblastic leukemia (Zhang et al., 2021). The crystallographic structure and coordinates of this proteomic system are deposited in the *Protein Data Bank* under code **7DW5** and present a resolution of 2.83 Å. Since this macromolecular system does not present a ligand or inhibitor complexed with it, the active site region chosen to perform the docking study was taken in the region of the linker junction of the two monomers of the DUX4_1-150_-DNA_ERG_, and was considered the center of the coordinates of the position of the CE1:His78 residue atom of the A chain.

Cyclic-dependent kinase 6 (CDK6) is one of the enzymes responsible for regulating cell cycle. The U251 cell modifies its cycle when glioblastoma is established in the body system (Bronner et al., 2019). The CDK6–cyclin D complexes with CKD4 complex are involved in the phosphorylation and inactivation of the retinoblastoma protein. Proper inhibition of these CDKs is a therapeutic target for cancer treatment. In this work, we select a CDK6 transferase enzyme complexed with a brain-penetrant potent inhibitor (**NIJ**) to perform our docking study related to the U251 cell proteome. The crystallographic structure and coordinates of this CDK6 system are deposited in the *Protein Data Bank* under code **6OQO** and present a resolution of 1.98 Å.

The abnormal cell growth pattern of cancer cells is regulated by the decrease of the extracellular pH. The human carbonic anhydrase (CA) enzyme, which is expressed in MDA-MB-231 cell genome codification, is the machinery responsible for catalyzing the conversion of CO_2_ to HCO_3_^−^ and H^+^ ion (Petreni et al., 2020) by regulation of the pH outside the cell membrane. The crystallographic structure and coordinates of the CA enzyme, complexed with a potent inhibitor named QYA, deposited in the *Protein Data Bank* under the code **6VJ3** with a resolution of 1.35 Å was used to perform one of the docking studies in the present work to evaluate and model a possible method via inhibition of the molecules studied here.

To better perform our docking study and validate our protocol, we primarily run a pre-docking study and a re-docking study with the known original complexed inhibitors for each protein target. The pre-docking study was carried out with each ligand originally complexed with the protein selected for each type of cell. The pre-docking study was performed using the ASP fitness score function under the systematic search of fix/fix receptor–ligand conformations, taking into account that all rotatable bonds were kept fixed, sequentially the best-fit pose superposition related with the original crystallographic structure using as a parameter the lowest root-mean-square deviation (RMSD) value when both molecular structures were compared. All crystallographic protein structures studied were previously prepared using the protonation standard protocol established in GOLD software to add the hydrogen atoms according to the proper pKa for each residue of the proteins, especially respecting the most probable protonation state of all histidine, glutamic acid, and aspartic acid residues, as well as the best dihedral angle of rotation of all asparagine and glutamine residues taken into account. The water molecules were completely removed from all crystallographic structures, as well as any other kind of molecules that did not correspond to protein residues. The original ligands complexed with the proteins have the coordinates of their molecular structures extracted to be used as ligands during the pre-docking study and compared posteriorly with the best-fitted pose obtained. After a systematic study to define the best size of the binding active site region, we took into account the regions including all residues at 8, 10, and 12 Å of distance around the region of the original ligand, and we observed that the better fit and pose conformations for the original ligands and also our molecules were the binding site regions considering all residues with 12 Å of the distance of the center of the coordinates of the original complexed ligand. The protocol validated with the pre-docking study was taking the population size as 100, performing 200,000 operations, and fixing the mutation frequency as 10 with the crossover mutation frequency.

As a result of our pre-docking study, using the redock methodology, we obtained the RMSD between the best-fitted docked pose and the crystallographic coordinates of the original ligands established as 0.2 Å for the UDP ligand (4KCG crystallographic structure of the protein from NALM-6 cells). No RMDS value was found for 7DW5 crystallographic structure of the protein from 697 cells since this structure does not have any ligand complexed with it. The RMSD value was 0.48 Å for the NIJ ligand at (6OQO crystallographic structure of the protein from U251 cells) and 0.56 Å for the QYA ligand at (6VJ3 crystallographic structure of the protein from MDA-MB-231 cells). The superposition of all poses obtained in the redock study is shown in Supplementary Figure S32, in the supplementary section. Supplementary Figure S33 shows all the interactions highlighted in the complex formed between the known ligands with each target protein. These low values of the RMSD gave a confusability rate to run a new docking study with our six ligands. After those tight procedures and good validation of the docking protocol, we finally performed the docking study of our six ligand molecules, composed of four nickel-complexed compounds and two organic ligands.

## 3 Results and discussion

**FIGURE 1:**
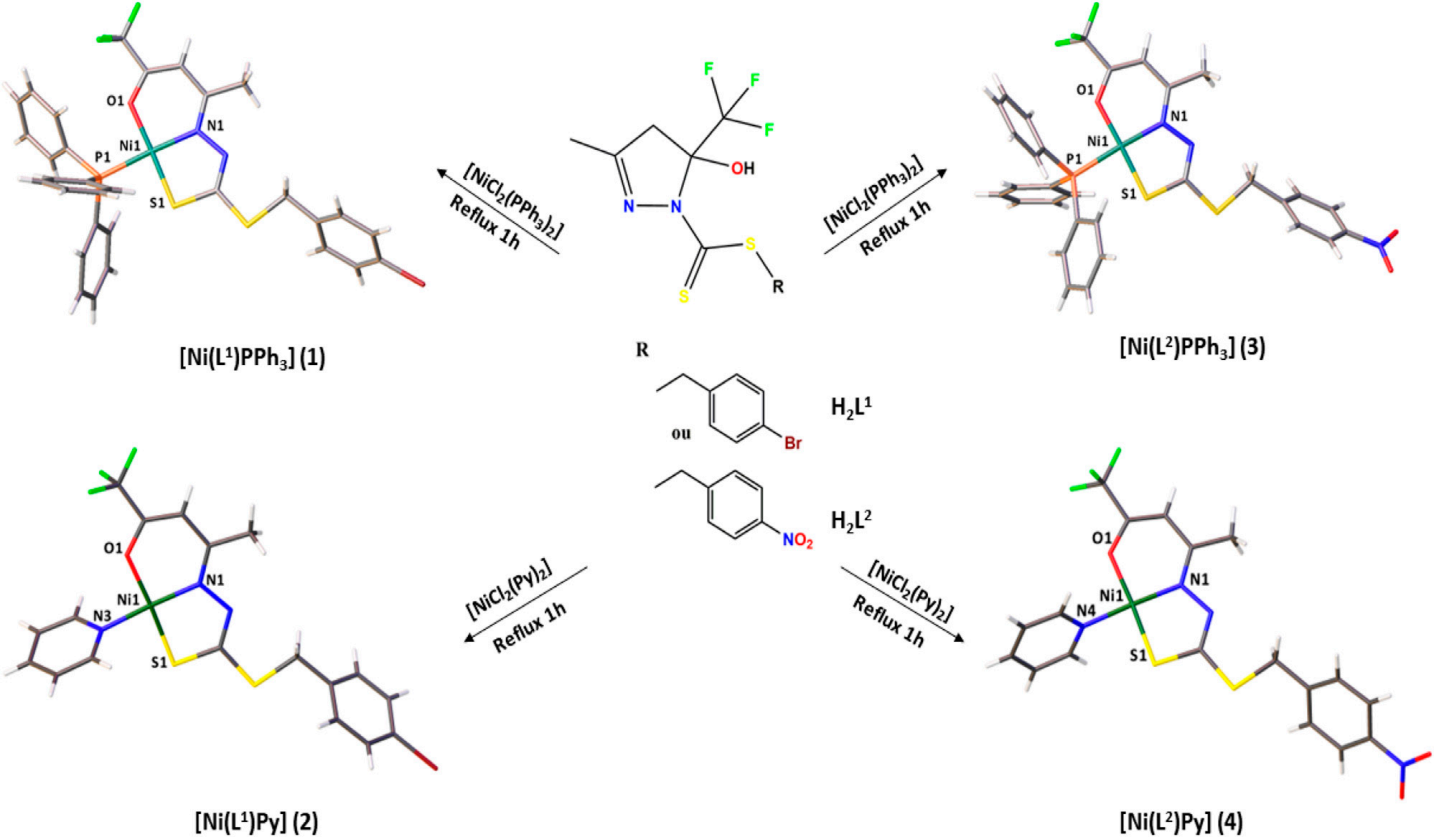
Synthesis of the Ni(II) complexes (1–4).

Two dithiocarbazate ligands, H_2_L^1^ and H_2_L^2^, and their nickel (II) complexes [Ni(L^1^)PPh_3_] 1), [Ni(L^1^)Py] 2), [Ni(L^2^)PPh_3_] 3), and [Ni(L^2^)Py] 4) were synthesized and characterized by physicochemical and spectroscopic methods. The crystal structures of the ligand H_2_L^1^ and complexes (1–4) were established by single-crystal X-ray diffraction, as presented in Figure 1.

### 3.1 Structural analysis

**FIGURE 2:**
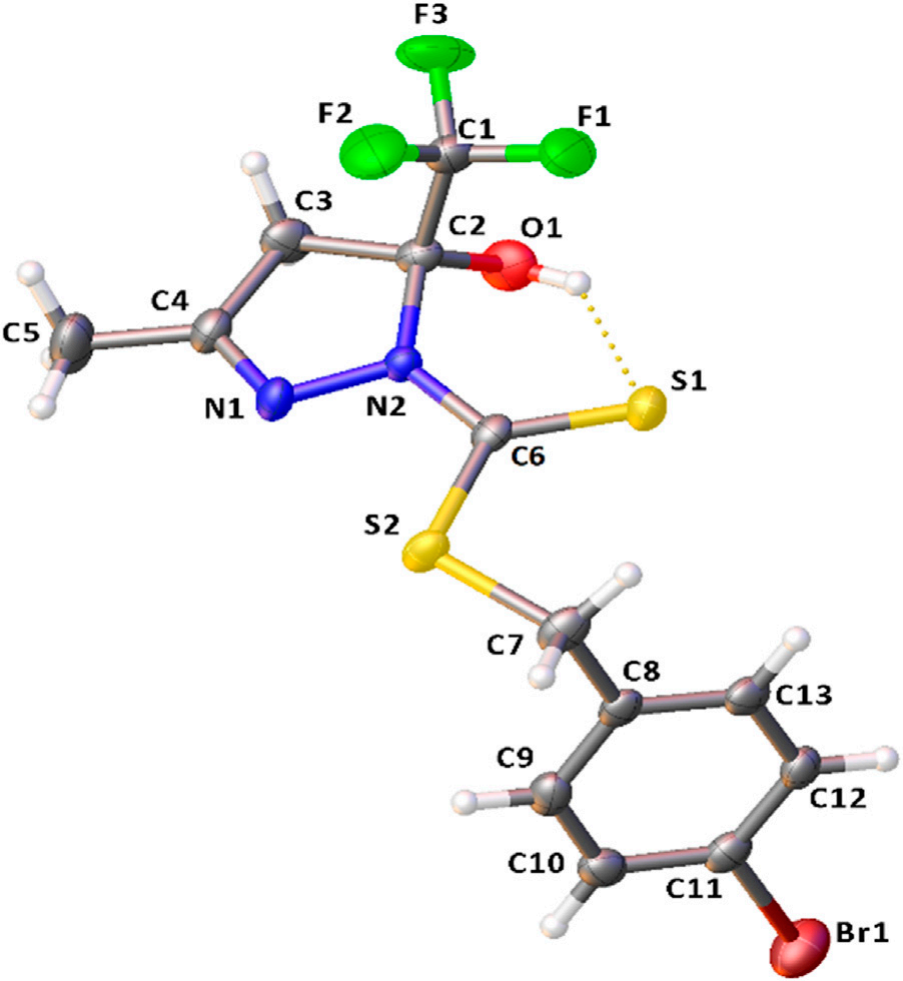
Molecular structure of H_2_L^1^ with crystallographic labeling (30% probability displacement). The intramolecular interaction is shown as a dashed line.

The crystal structure of H_2_L^1^ is established accurately by single-crystal X-ray diffraction (Figure 2). It is important to emphasize that dithiocarbazates derived from β-diketones could be predominantly in their cyclic or acyclic form. The crystal structure revealed a cyclic compound in the solid state, in which the structure was formed in the last step of the synthesis after a nucleophilic attack of the N (1) to the C (4) and the subsequent attack of N (2) to the C (2), forming a five-membered ring, similar to other compounds in the literature (Deflon et al., 2010; Pavan et al., 2010; Ali et al., 2011; De Sousa et al., 2011). Also, it should be underscored that this class of compounds presents the thione–thiol tautomeric equilibrium in solution. It is possible to observe that H_2_L^1^ is in thione form, evidenced by the bond length C (6)-S (1) of 1.665 (4) Å and C (6)-N (2) of 1.347 (4) Å. These bonds are very similar to other compounds already reported in the literature, such as the ligand 1-phenyl-1,3-butanedione-S-benzyl-dithiocarbazate synthesized by Sousa (De Sousa et al., 2011).

The bond angles observed between C1-C2-O1 of 110.7 (3)°, C1-C2-C3 of 109.7 (3)°, C1-C2-N2 of 110.3 (3)°, and C3-C2-O1 of 111.0 (3)° indicate that C2 is sp^3^-hybridized, and they are all close to 109.5°. The ligand structure also shows that each ring of the structure (C2-C3-C4-N1-N2) and (C8-C9-C10-C11-C12-C13) is nearly planar, but they are twisted concerning each other. The angle between the mean planes of the rings is 99.43 (13)°.

**FIGURE 3:**
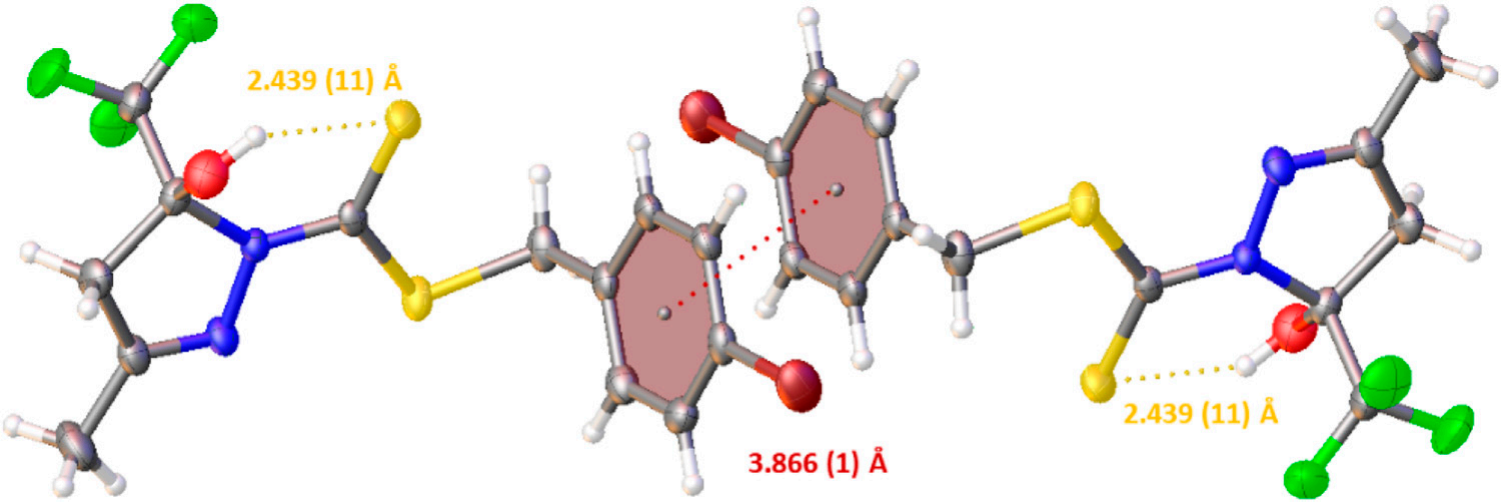
Projection view of H_2_L^1^ showing an intramolecular hydrogen bond and the π⋅⋅⋅π stacking interactions (as a dashed line).

It was also possible to observe an intramolecular hydrogen bond between S1 and H1of 2.439 (11) Å and a π–π stacking interaction with a distance of 3.866 (1) Å between the centroids and displacement of 1.755 (1) Å. These interactions play an important role in the structural arrangement and help organize the crystal structure of H_2_L^1^. Figure 3 illustrates the interactions observed in H_2_L^1^.

**FIGURE 4:**
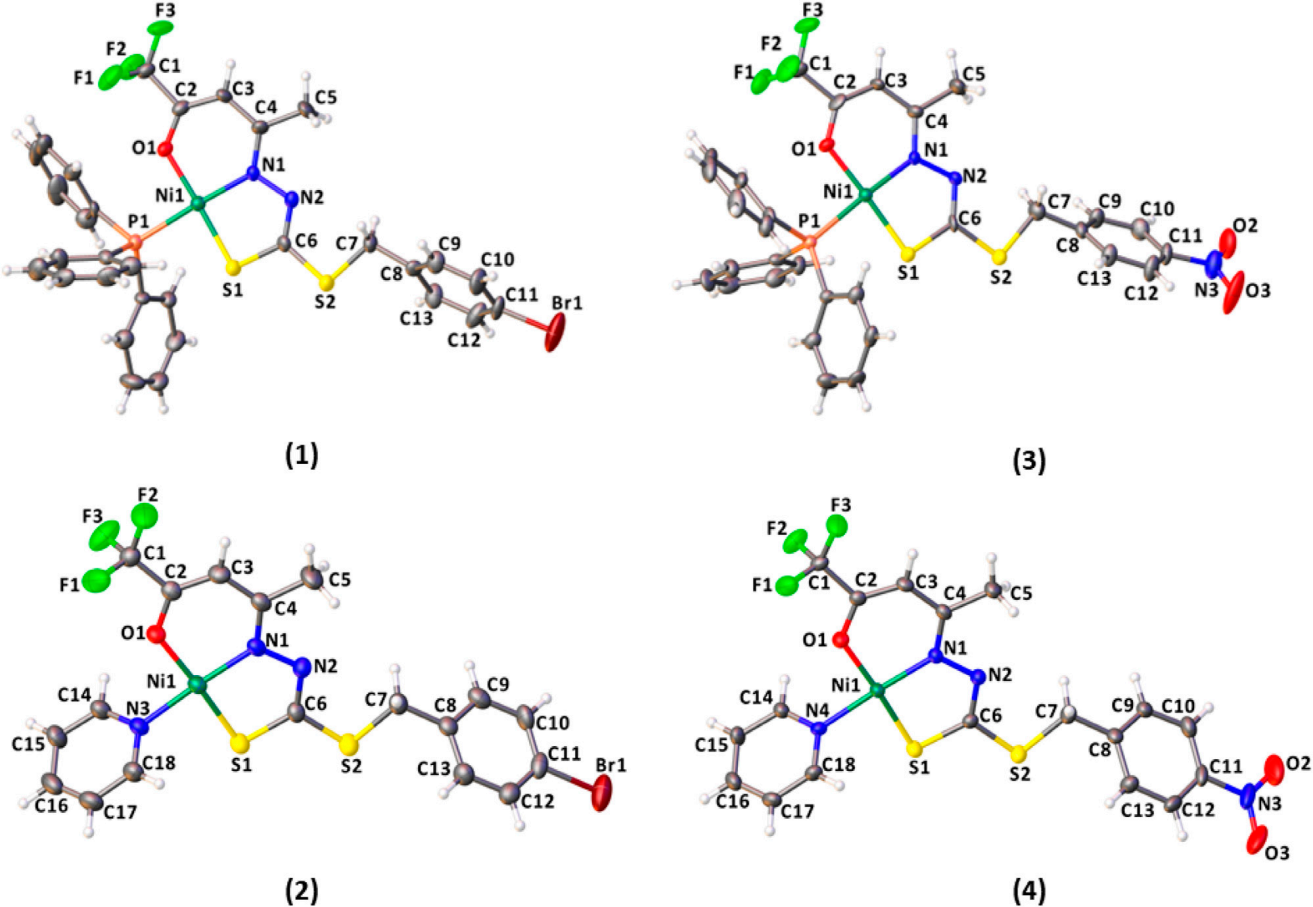
Molecular structure of the complexes (1–4) with crystallographic labeling (30% probability displacement).

The structural analysis of the complexes (1–4) revealed the Ni(II) atoms in a square planar coordination geometry, as presented in Figure 4. The ligands H_2_L^1^ or H_2_L^2^ are coordinated to the metal center by the *ONS* atoms in a deprotonated and dianionic form. The coordination sphere is completed by coordination with neutral and monodentate coligands, including triphenylphosphine (1 and 3) or pyridine molecule (2 and 4). Comparing the complexes, less solubility is observed for (1) and (3), due to the slightly higher steric effect. The dithiocarbazates are coordinated to the Ni(II) atoms in acyclic form, where the established six and five-membered chelate rings are nearly planar; however, there is a twist in the final part of the ligand, with the twist angle between the planar rings being between 51.9 (2) and 58.8 (3)°.

The bond lengths of the *ONS* donor system of H_2_L^1^ and H_2_L^2^ are between 1.866 (5) and 1.910 (8) Å to Ni1-N1, 2.128 (3) and 2.147 (2) Å to Ni1-S1, and 1.845 (6) Å and 1,861 (3) Å to Ni1-O1, and these distances are similar to the bond lengths reported in the literature for other similar Ni(II) complexes (Cavalcante et al., 2019; Yusof et al., 2019; Zahan et al., 2019; Bilyj et al., 2021; Manan and Mohammat, 2022). The bond length Ni(1)-P (1) is 2.223 (3) Å in (1) and 2.216 (3) Å in (3), in agreement with the range found for Ni(II) complexes (Elsayed et al., 2021; Lima et al., 2021). In addition, the Ni-N bond length with the pyridine nitrogen is 1.915 (4) Å in (2) and 1.923 (6) Å in (4), and the results agree with those of the related compounds (Lima et al., 2020). Selected bond distances and bond angles for H_2_L^1^ and complexes (1–4) are listed in Table 2.

**TABLE 2:** Selected bond lengths (Å) and angles (°) for H_2_L^1^ and the complexes (1–4).

bond lengths (Å)										
	H_2_L^1^		(1)		(2)		(3)		(4)	
O1-Ni1	-		1.852 (6)		1.861 (3)		1.845 (6)		1.857 (5)	
N1-Ni1	-		1.910 (8)		1.876 (4)		1.885 (7)		1.866 (5)	
S1-Ni1	-		2.130 (3)		2.147 (2)		2.128 (3)		2.146 (2)	
P1-Ni1	-		2.223 (3)		-		2.216 (3)		-	
N*-Ni1	-		-		1.915 (4)		-		1.923 (6)	
C2-C3	1.520 (5)		1.368 (14)		1.359 (6)		1.357 (13)		1.357 (9)	
C6-S2	1.748 (4)		1.746 (11)		1.761 (5)		1.764 (10)		1.756 (7)	
N2-C6	1.347 (4)		1.287 (11)		1.300 (5)		1.285 (11)		1.302 (8)	
C6-S1	1.665 (4)		1.741 (11)		1.726 (5)		1.727 (10)		1.724 (7)	
C4-N1	1.279 (4)		1.337 (12)		1.316 (5)		1.321 (11)		1.325 (8)	
C2-O1	1.382 (4)		1.283 (11)		1.296 (5)		1.279 (11)		1.288 (8)	

Bond angles (°)										
	H_2_L^1^		(1)		(2)		(3)		(4)	
S1-Ni1-P1	-		89.59 (12)		-		89.43 (11)		-	
S1-Ni1-N1	-		87.4 (3)		88.15 (13)		87.6 (3)		88.26 (17)	
S1-Ni1-O1	-		177.3 (2)		174.35 (10)		177.5 (2)		174.23 (17)	
P1-Ni1-N1	-		176.0 (3)		-		176.5 (3)		-	
P1-Ni1-O1	-		88.2 (2)		-		88.6 (2)		-	
O1-Ni1-N1	-		94.7 (3)		95.75 (16)		94.3 (3)		95.6 (2)	
O1-Ni1-N*	-		-		86.70 (15)		-		86.7 (2)	
N1-Ni1-N*	-		-		176.64 (18)		-		177.3 (2)	
S1-Ni1-N*	-		-		89.59 (12)		-		89.55 (17)	
C1-C2-O1	110.7 (3)		112.6 (11)		113.0 (5)		111.8 (10)		112.5 (6)	
C1-C2-C3	109.7 (3)		119.7 (11)		120.1 (5)		120.6 (10)		120.2 (6)	
C3-C2-O1	111.0 (3)		127.8 (11)		126.9 (5)		127.7 (10)		127.2 (6)	

In all complexes, the ligand was coordinated to the Ni(II) atom in the *E* isomer and thiol tautomer. This can be observed in the bond lengths S (1)-C (6), between 1.724 (7) Å and 1.741 (11) Å, and also C (6)-N (2), between 1.285 (11) Å and 1.302 (8) Å, which show characteristics of single and double bonds, respectively. The same behavior is observed for Ni(II) complexes with dithiocarbazate ligands already reported in the literature. (Elsayed et al., 2021).

To predict the geometry of the complex, the (Okuniewski et al., 2015) parameter τ_4_ was used, defined as *τ*_4_ = 360°-(α + β)/360°-2θ, where α and β are the two greatest angles of the coordination polyhedron, where 0 indicates square planar and 1 tetrahedral geometry. For the complexes (1–4), the calculated values were found between 0.042 and 0.064 and are consistent with the observed distorted square planar geometries and in agreement with other studies reported in the literature (Elsayed et al., 2021; Lima et al., 2021).

In addition, non-classical intermolecular interactions were observed between the O (1) and H (17) atoms of the triphenylphosphine molecule with a distance of 2.609 (7) Å in the complex (1) and 2.613 (7) Å in the complex (3). Interactions between C (12)-H (12)⋅⋅⋅N (2) with distances of 2.669 (8) Å in (1) and 2.736 (8) Å in (3) are also observed (Supplementary Figure S1, S3). The X-ray diffraction data also show intermolecular interaction between C (14)-H (14)⋅⋅⋅F (1) with a distance of 2.414 (3) Å in the complex (2) and 2.397 (5) Å in the complex (4), as illustrated in Supplementary Figures S2 and S4 in SI.

### 3.2 Hirshfeld surface analysis

To analyze the intermolecular interactions of the compounds, the CIF files (crystallographic information files) obtained from X-ray single-crystal measurements were used to generate the Hirshfeld surfaces (HS) in the *d*_*norm*_ function by the program CrystalExplorer (Turner et al., 2017). The 3D *d*_*norm*_ surface shows a color pattern between red, white, and blue, which allows qualitatively evaluating the contacts that most contribute to the supramolecular arrangement of the compound. Red regions indicate contacts shorter than the sum of the van der Waals radii showing strong interactions, while blue regions show more distant contacts (Spackman and Jayatilaka, 2009; Cavalcante et al., 2022; Yusof et al., 2022).

**FIGURE 5:**
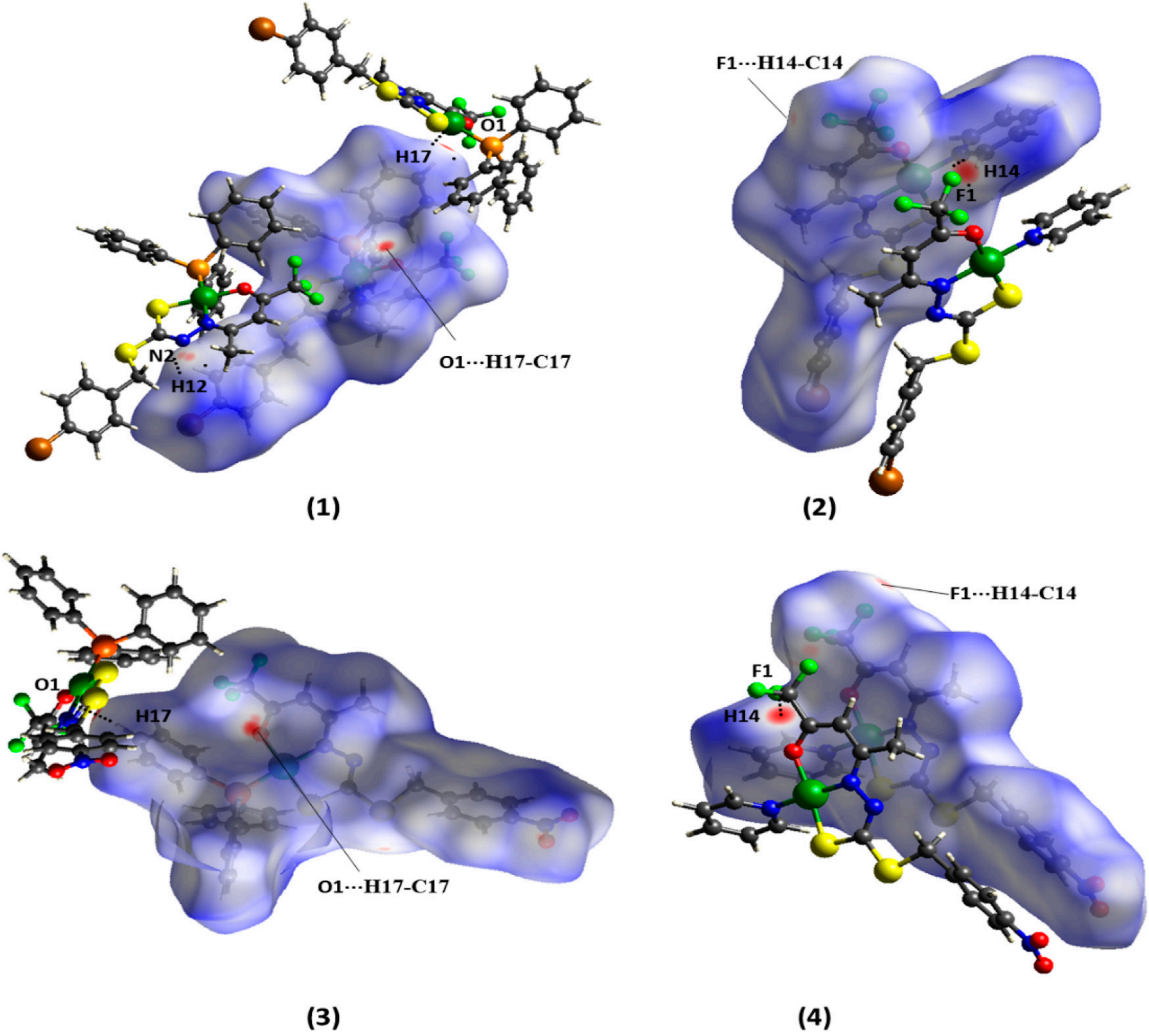
Hirshfeld surface of the complexes (1–4) mapped with *d*_*norm*_.

The Hirshfeld surface by the *d*_*norm*_ function for the ligand H_2_L^1^ shows practically only white and blue regions, indicating weak hydrogen bonds (Supplementary Figure S5 in SI) (Takjoo et al., 2010; Santiago et al., 2020). On the other hand, the maps generated by the *d*_*norm*_ function for the complexes show red spots, indicative of stronger intermolecular interactions than those of the free ligand, as shown in Figure 5. These interactions could be identified as non-classical hydrogen bonds O⋅⋅⋅H-C and N⋅⋅⋅H-C in (1), O⋅⋅⋅H-C in (3), and F⋅⋅⋅H-C in (2) and (4), which are in agreement with those observed by the X-ray single-crystal diffraction.

**FIGURE 6:**
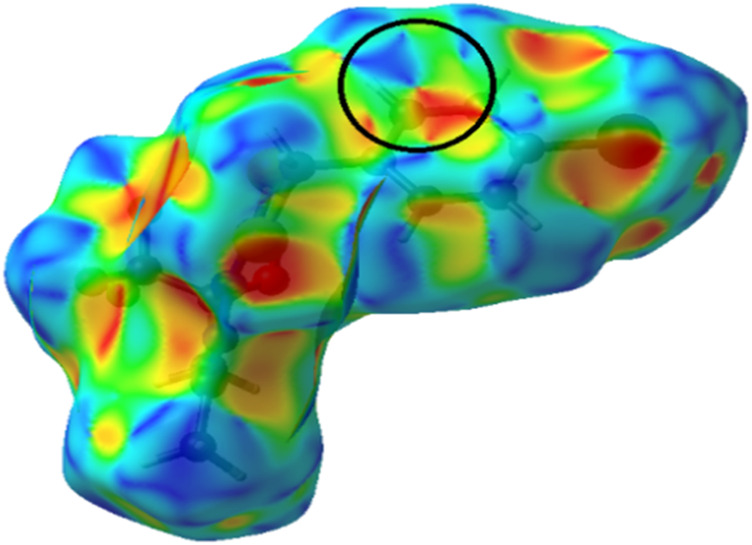
Hirshfeld surface mapped in shape index for H_2_L^1^.

The shape index surface was used to investigate the π–π stacking interactions for the crystal packing of the compounds, which are indicated by a pattern of complementary bumps and hollows, evidenced by the red and blue triangles, respectively (Spackman and Jayatilaka, 2009; Santiago et al., 2020; Cavalcante et al., 2022). The shape index indicates the presence of π∙∙∙π stacking interactions between the rings of the dithiocarbazate for H_2_L^1^, with a distance of 3.866 (1) Å between the centroids, as observed in Figure 6.

**FIGURE 7:**
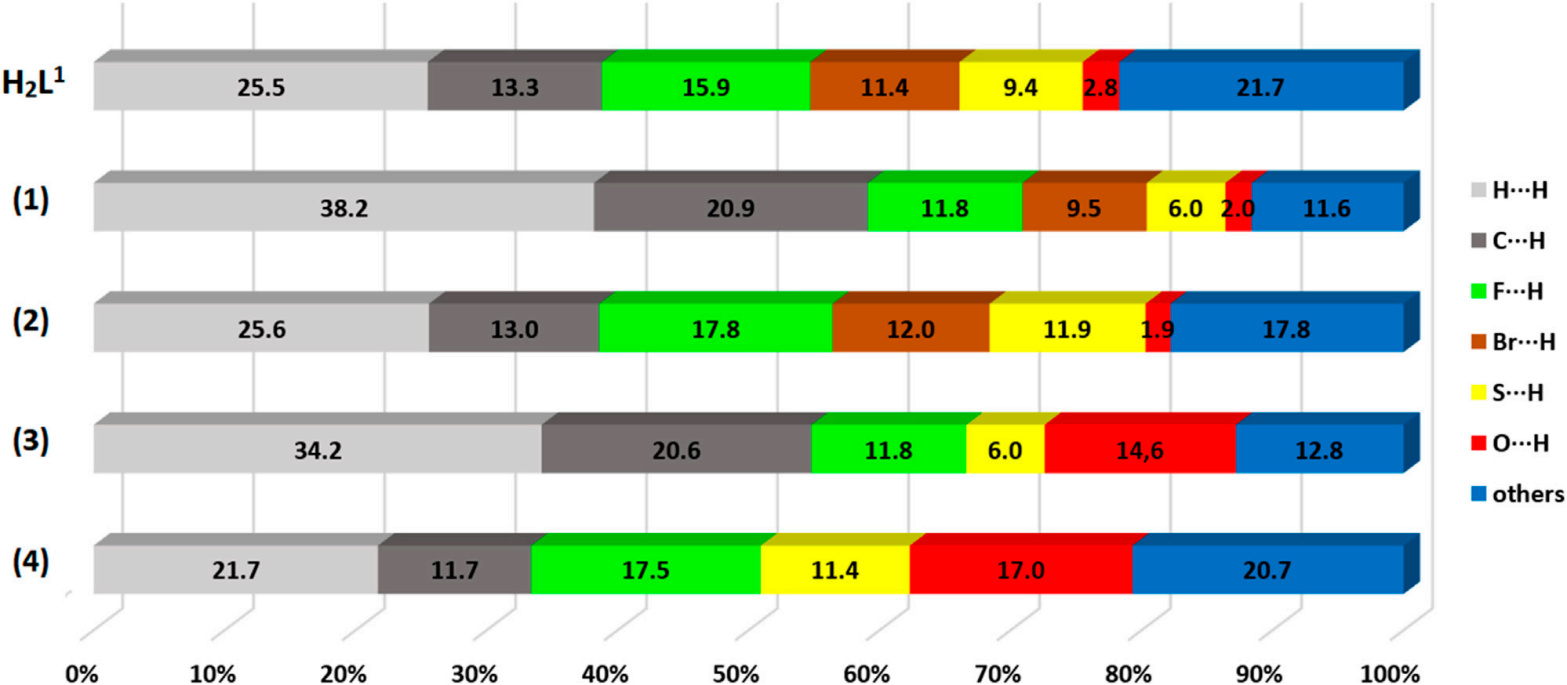
Percentage contribution of close contacts for H_2_L^1^ and the complexes (1–4).

In addition, fingerprint graphs were obtained for the compounds (Supplementary Figure S6–S10 in SI), to obtain quantitative data on the contacts (even distant ones) that most contribute to formation of the crystals. The contribution percentages referring to the fingerprint graphs are summarized in Figure 7. The results indicated that there is a large change in the contribution percentages of each pair of atoms between the H_2_L^1^ ligand and its complexes, (1) and (2). It is also possible to observe that the change of the final substituent of the dithiocarbazate (-NO_2_ or -Br) partially alters the percentages in each pair, (1) and (3) or (2) and (4), although there is a structural similarity between the complexes. The greatest contributions of the interactions are between H⋅⋅⋅H, C⋅⋅⋅H, O⋅⋅⋅H, Br⋅⋅⋅H, and F⋅⋅⋅H for the compounds studied, with contacts contributing between 1.9% and 38.2%.

### 3.3 Spectroscopic analyses

The vibrational spectra recorded by FT-IR analysis for the H_2_L^1^ and H_2_L^2^ and complexes (1–4) are shown in Electronic Supplementary Information (Supplementary Figure S11–S16 and Supplementary Table S1). In general, the spectra show important bands which were attributed to ν(C=S), ν(C–S), ν(O–H), ν(N–N), ν(C=C), and ν(C=N), in similarity with previously published works of other Ni(II) complexes with dithiocarbazate ligand-based (Qiu et al., 2014; Cavalcante et al., 2019; Lima et al., 2021; Zahan et al., 2021; Yusof et al., 2022).

Comparing the FT-IR spectra of the ligands and complexes, we observed the ν(O–H) for H_2_L^1^ and H_2_L^2^ in 3,199 cm^–1^ and 3,151 cm^–1^, respectively, and this band is not detected in the spectra of the complexes, indicating the deprotonation of the dithiocarbazates with the coordination of the thioenolate sulfur to the Ni(II) atoms. Additionally, the ν(C=S) between 1,300 and 1,310 cm^–1^ in the free ligands is not observed in the spectra of the complexes (1–4). This result is in agreement with the X-ray diffraction analysis, in which the ligands change from thione to thiol tautomer after complexation (Deflon et al., 2010; Elsayed et al., 2021). Another band involved in the coordination sphere that changes after complexation is attributed to the azomethine ν(C=N), which is observed at lower frequencies in the spectra of the complexes, indicating N-Ni(II) coordination. The same occurs with the ν(N–N) band, found at lower frequencies after complexation (Deflon et al., 2010; Elsayed et al., 2021). Bands at 689 cm^–1^ are also observed in the spectrum of complexes (2) and (4), which can be attributed to δ(Py), indicative of Ni–Py coordination (Cavalcante et al., 2019; Santra et al., 2020; Gou et al., 2021; Cavalcante et al., 2022). Bands of ν(Ni–PPh_3_) are also identified in the spectra of (1) and (3) at 693 cm^–1^ due to the coordination of the nickel atom to the triphenylphosphine molecule (Lima et al., 2021).

In the absorption spectra of the free ligands H_2_L^1^ and H_2_L^2^, it is possible to observe bands corresponding to the π→π* transition of the azomethine group at around 275–297 nm in MeOH and around 284–302 nm in DMSO (Takjoo et al., 2011; Alam et al., 2019; Cavalcante et al., 2022). In the H_2_L^1^ ligand spectrum, bands at 238 nm and 389 nm in MeOH and 258 nm and 396 in DMSO are also observed, which have already been attributed as being π→π* transitions of aromatic groups and n→π* of dithiozabazate moiety, respectively (Takjoo and Centore, 2013; Alam et al., 2019). In the spectra of the complexes, it is possible to observe a hypsochromic shift of the π→π* band corresponding to the π→π* transition of the azomethine, which decreases to the range of 259–268 nm in MeOH and 265–271 nm in DMSO, which is indicative of the coordination of the group to the Ni(II) atom (Lima et al., 2021). It was still possible to observe bands in the spectrum of the four complexes that can be attributed to the ligand–metal charge transition (LMCT) in the range of 368–372 nm in MeOH and 378–381 nm in DMSO, already attributed in other studies as being an indication of the coordination of the dithiocarbazate to the Ni(II) atom by the sulfur atom of the thiolate group (Nanjundan et al., 2017; Alam et al., 2019; Elsayed et al., 2021). The electronic spectra are shown in SI (Supplementary Figure S28–S31 and Supplementary Tables S8).

### 3.4 Mass spectrometry

**FIGURE 8:**
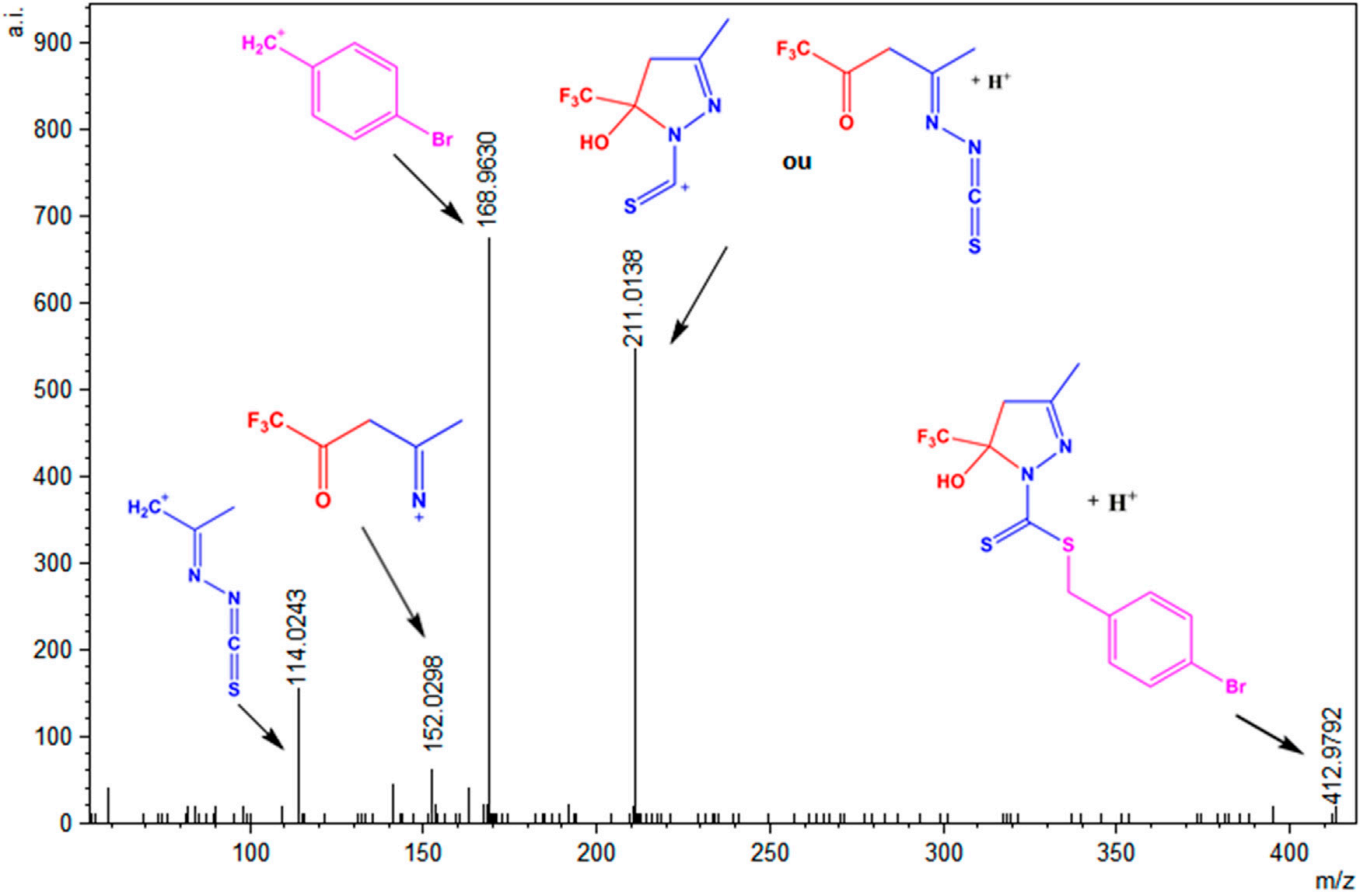
ESI(+)-MS/MS spectrum for H_2_L^1^.

To evaluate the species present in the solution, electrospray_ionization (ESI(+)–MS(/MS)) mass spectra were obtained in the positive mode for the ligands H_2_L^1^ and H_2_L^2^ and the Ni(II) complexes (1)–(4) (Lalli et al., 2013; Hu et al., 2014). Supplementary Figures S17–S22 show the ESI(+)-MS mass spectra for the compounds and show that all of them present peaks of the isotopic distributions, as expected for the [M + H]^+^ ions (Gohlke, 2018). The ESI(+)-MSMS spectrum for H_2_L^1^ is shown in Figure 8, and it is possible to observe a peak in m/z = 412.9792, which corresponds to the [M + H]^+^ species. The peak in m/z = 211.0138 can be attributed to the species formed after the loss of the S-benzyl group. Other peaks were observed in m/z = 152.0298 and m/z = 114. 0243, corresponding to the fragments formed after the loss of thiocyanic acid (HSCN) and C_2_HF_3_O, respectively. In addition, the base peak in m/z = 168.9630 corresponds to a fragment of the final portion of the dithiocarbazate (Takjoo et al., 2016; Cavalcante et al., 2019; Lima et al., 2020). The same behavior is observed for H_2_L^2^, with a spectrum illustrated in Supplementary Figure S23 in the SI.

**FIGURE 9:**
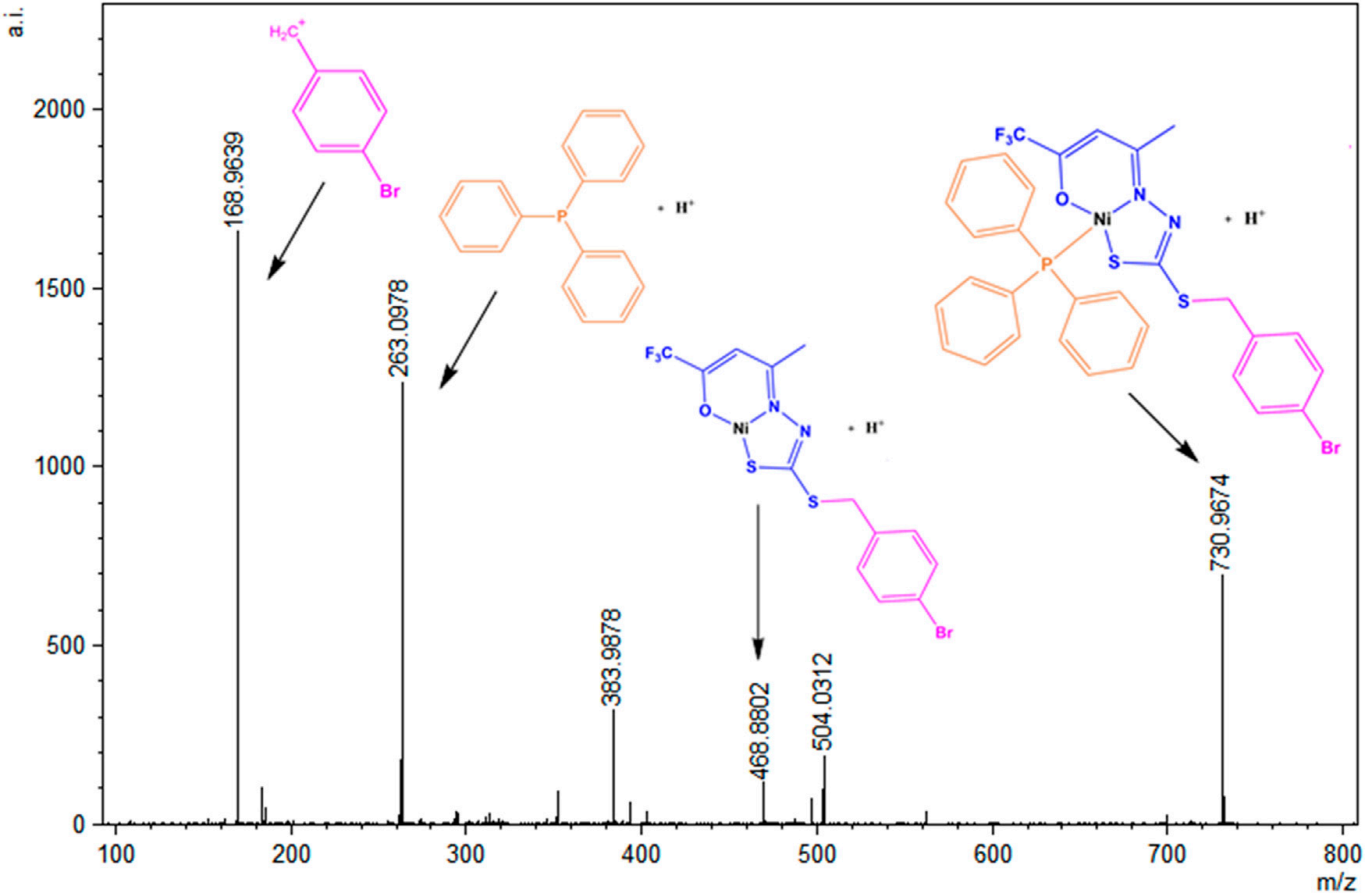
ESI(+)-MS/MS spectrum for (1).

The ESI (+)-MSMS spectrum for the complex (1) is presented in Figure 9, and it is possible to identify the presence of the species [M + H]^+^ in m/z = 730.0974. The peak in m/z = 468.8802 can be identified as a fragment formed after the loss of a triphenylphosphine molecule, and the peak at m/z = 263 corresponds to the protonated PPh_3_ molecule. The base peak at m/z = 168.0978 corresponds to a fragment of the H_2_L^1^ ligand, also identified as the base peak in the spectrum of the free dithiocarbazate.

**FIGURE 10:**
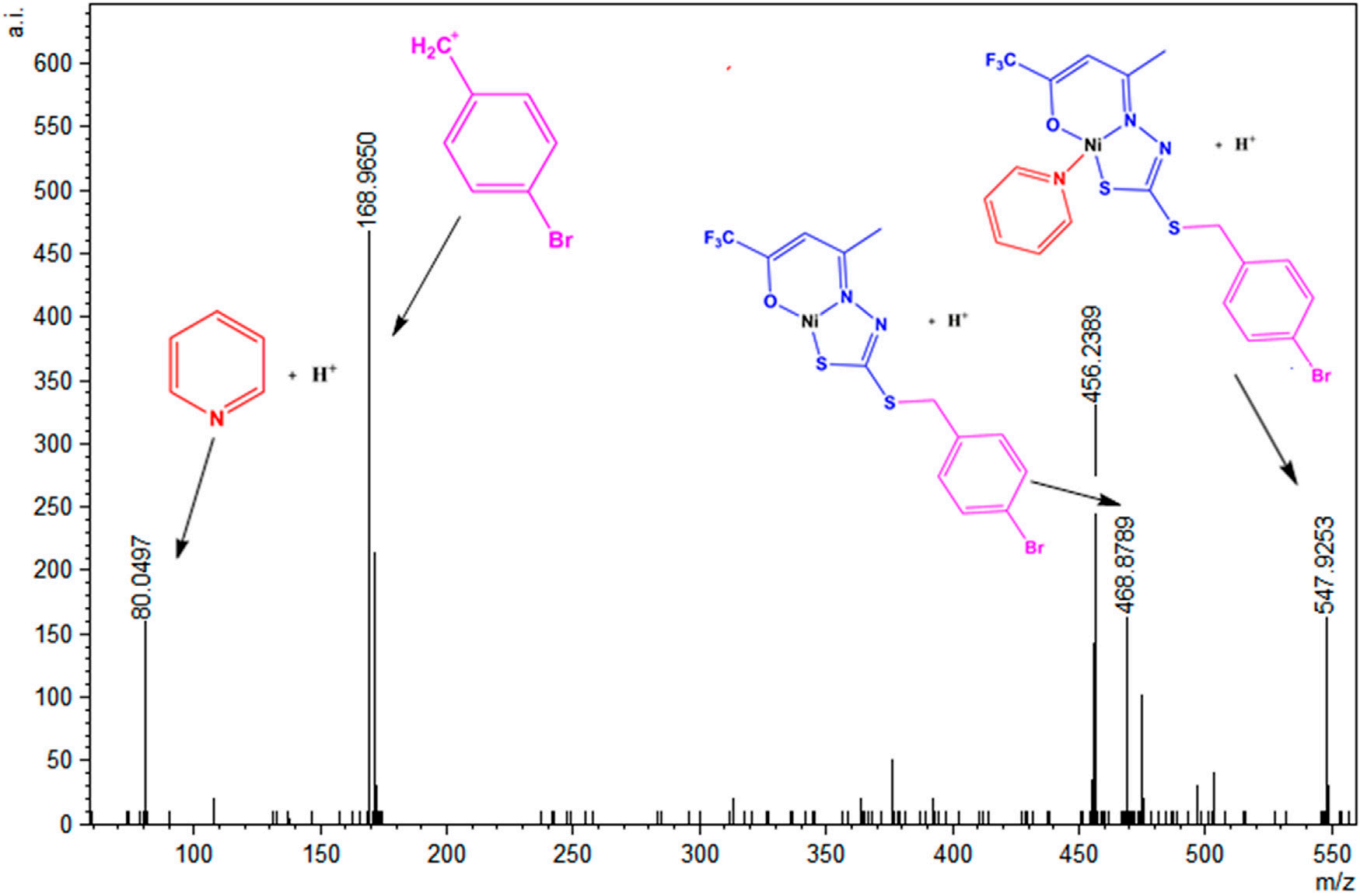
ESI(+)-MS/MS spectrum for (2).

A similar behavior was observed in the solution for (2), which has its ESI-MSMS spectrum illustrated in Figure 10. The peak at m/z = 547.9253 corresponds to the protonated molecular ion, and the peak at m/z = 468.8789 can be identified after the loss of a pyridine molecule. The base peak corresponds to a fragment of the H_2_L^1^ ligand, and the peak in m/z = 80.0497 corresponds to the species (Py)^+^.

Additionally, in the ESI(+)-MSMS for complexes (3) and (4) (Supplementary Figure S24. S25), the first peak corresponds to the protonated molecular ion [M + H]^+^ in m/z = 698.0455 in (3) and m/z = 514.9943 in (4). The loss coligand forms the species [Ni(L_2_)]^+^ in m/z = 435.9588 and m/z = 435.9527 in (3) and (4), respectively. Fragments of protonated triphenylphosphine are also found for complex (3) in m/z = 263.0975 and the species (Py)^+^ in m/z = 80.0507 for complex (4). Furthermore, a fragment of the final part of the H_2_L^2^ ligand is observed in both spectra in m/z = 136.0396 or m/z = 136.0423. The observed results corroborate the other characterization techniques and suggest the same structures present in the solid state and the solution.

### 3.5 ^1^H NMR spectra

The signals at the ^1^H NMR spectra of H_2_L^1^ and H_2_L^2^ indicate their cyclic form in solution, as observed in the solid state. There are doublets at 3.32 ppm and 3.64 ppm for H_2_L^1^ and 3.32 ppm and 3.65 ppm for H_2_L^2^ attributed to the hydrogen atoms H3a and H3b. The doublets observed at 4.35 ppm and 4.30 ppm for H_2_L^1^ and 4.49 ppm and 4.55 ppm for H_2_L^2^ can be attributed to the hydrogen atoms H7a and H7b (Ali et al., 2011; De Sousa et al., 2011). It is also possible to observe that in a characteristic range, 734–8.18 ppm, the doublets correspond to the aromatic hydrogen atoms H9 and H10. In addition, a singlet at 2.05 ppm for H_2_L^1^ and 2.06 ppm for H_2_L^2^ can be attributed to the hydrogen H5 of the -CH_3_ groups. In addition, there is a broader signal in the range of 8.26–8.33 ppm in the spectrum of both dithiocarbazates, which can be attributed to the oxygen-bound H1a (Pavan et al., 2010; De Sousa et al., 2011; Cavalcante et al., 2019).

In the ^1^H NMR spectra for (1) and (3), the singlets appearing in the range 2.39–2.41 ppm can be attributed to the hydrogen H5 of -CH_3,_ and the singlets in the range 4.28–4.43 ppm correspond to the H7 hydrogen of the group -CH_2_. With the complexation and deprotonation of the ligand, signals are observed at 5.89 ppm and 5.58 ppm for (1) and (3), respectively, which can be attributed to the 3H hydrogen of the -CH group. In addition, the absence of a signal is observed at approximately 8.30 ppm, indicating deprotonation of the O-H group. The signals between 7.31 and 8.18 ppm correspond to the aromatic hydrogen atoms, with signals to the hydrogens of -PPh_3_ according to previously published studies (Elsayed et al., 2021; Lima et al., 2021).

The same behavior is observed for (2) and (4), where the singlets between 1.23 and 1.44 ppm are attributed to the hydrogen atoms of the -CH_3_ group and the singlets between 4.03 and 4.09 ppm are assigned to the hydrogen atoms of the -S-CH_2_ group. The hydrogen atoms of the -CH group correspond to the signals at 4.89 ppm for (2) and 4.71 ppm for (4). Additionally, the signals of the hydrogen atoms from the aromatic ring of the dithiocarbazates are observed as two doublets in a characteristic range. The signals that appear in the range 8.12–11.14 ppm can be attributed to the hydrogen atoms of pyridine, according to similar compounds in the literature. ^26,60 1^H NMR spectra data, multiplicity, and coupling constant (*J*) tables are given in SI (Supplementary Figure S26–S31, Supplementary Tables S2–S7).

### 3.6 Biological activity analysis

The cytotoxicity of all compounds was evaluated *in vitro* against four human cancer cell lines. Notably, the compounds showed biological activity in a low range of concentration, which indicates high efficiency as potential drugs, but the response varied according to the cell type (Table 3).

**TABLE 3:** Cytotoxic activity of the ligands H_2_L^1^ and H_2_L^2^ and the complexes (1–4). The results are presented as the inhibition concentration that causes a 50% decrease in cell growth (IC_50_) against cancer cells (values estimated by non-linear regression of data from viability assessment).

IC_50%_ (inhibitory concentration 50%) ± SD						
Cell line	H_2_L^1^	H_2_L^2^	(1)	(2)	(3)	(4)
NALM-6	25.5 ± 0.016	12.5 ± 0.028	40.3 ± 0.029	8.5 ± 0.022	20.6 ± 0.030	17.2 ± 0.030
697	27.4 ± 0.010	27.1 ± 0.012	23.6 ± 0.098	30.3 ± 0.014	65.6 ± 0.020	13.4 ± 0.023
U251	29.7 ± 0.024	29.7 ± 0.019	>150	23.3 ± 0.016	>150	59.9 ± 0.029
MDA-MB-231	83.3 ± 0.096	74.8 ± 0.036	69.6 ± 0.066	10.5 ± 0.020	37.6 ± 0.140	21.9 ± 0.031

**FIGURE 11:**
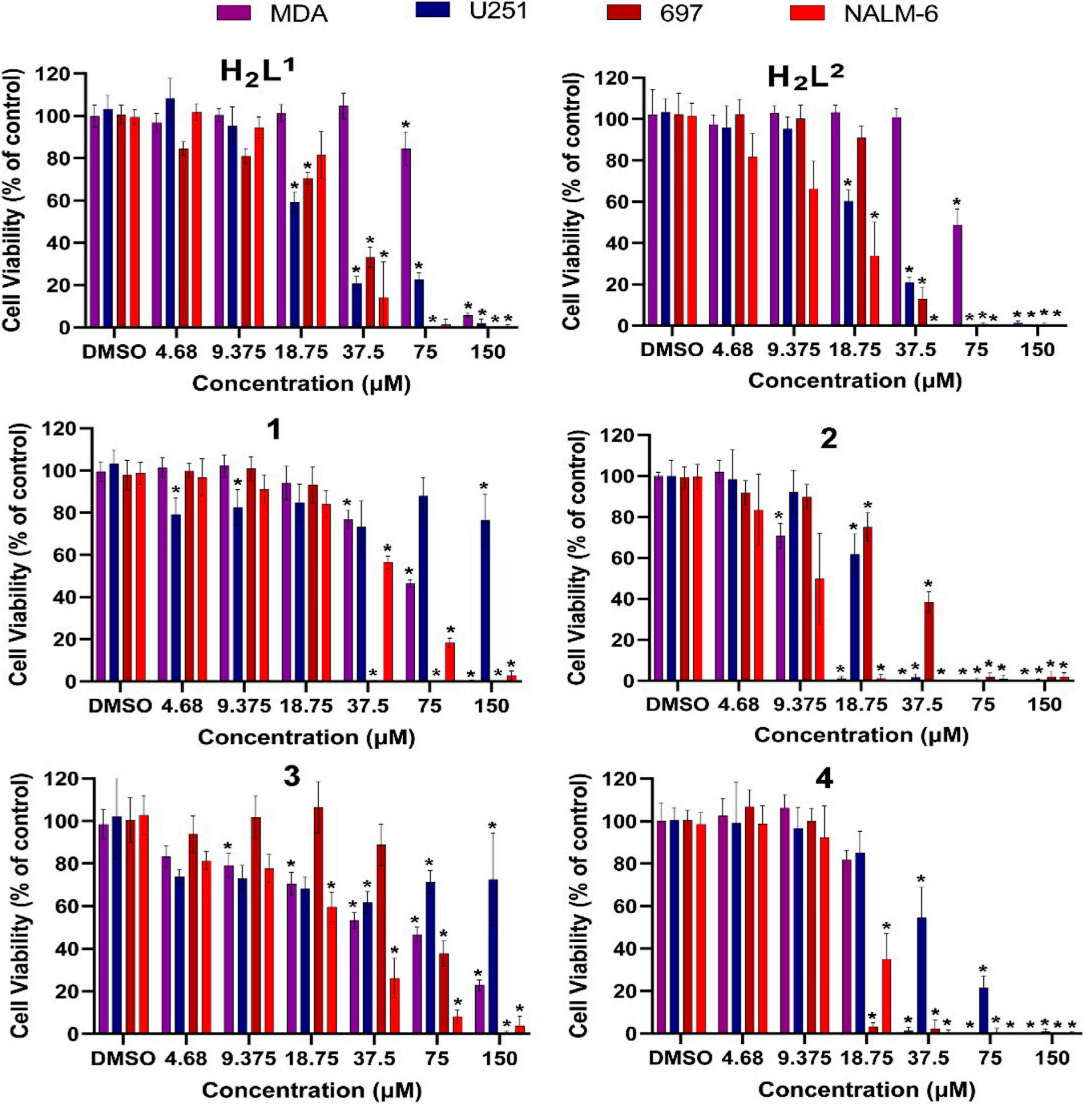
Evaluation of cytotoxic effects by MTT/resazurin assay. Concentrations lower than 4.68 µM were deleted since their effects were negligible. The asterisk indicates that cell viability is significantly different from the respective DMSO control (*p* < 0.5, Kruskal–Wallis followed by Dunn’s comparison test). DMSO at 0.01% did not affect the cell viability of any cell line.

A difference in susceptibility (see IC_50%_) as well as different response profiles among the cell lines was evidenced (Figure 11).

**FIGURE 12:**
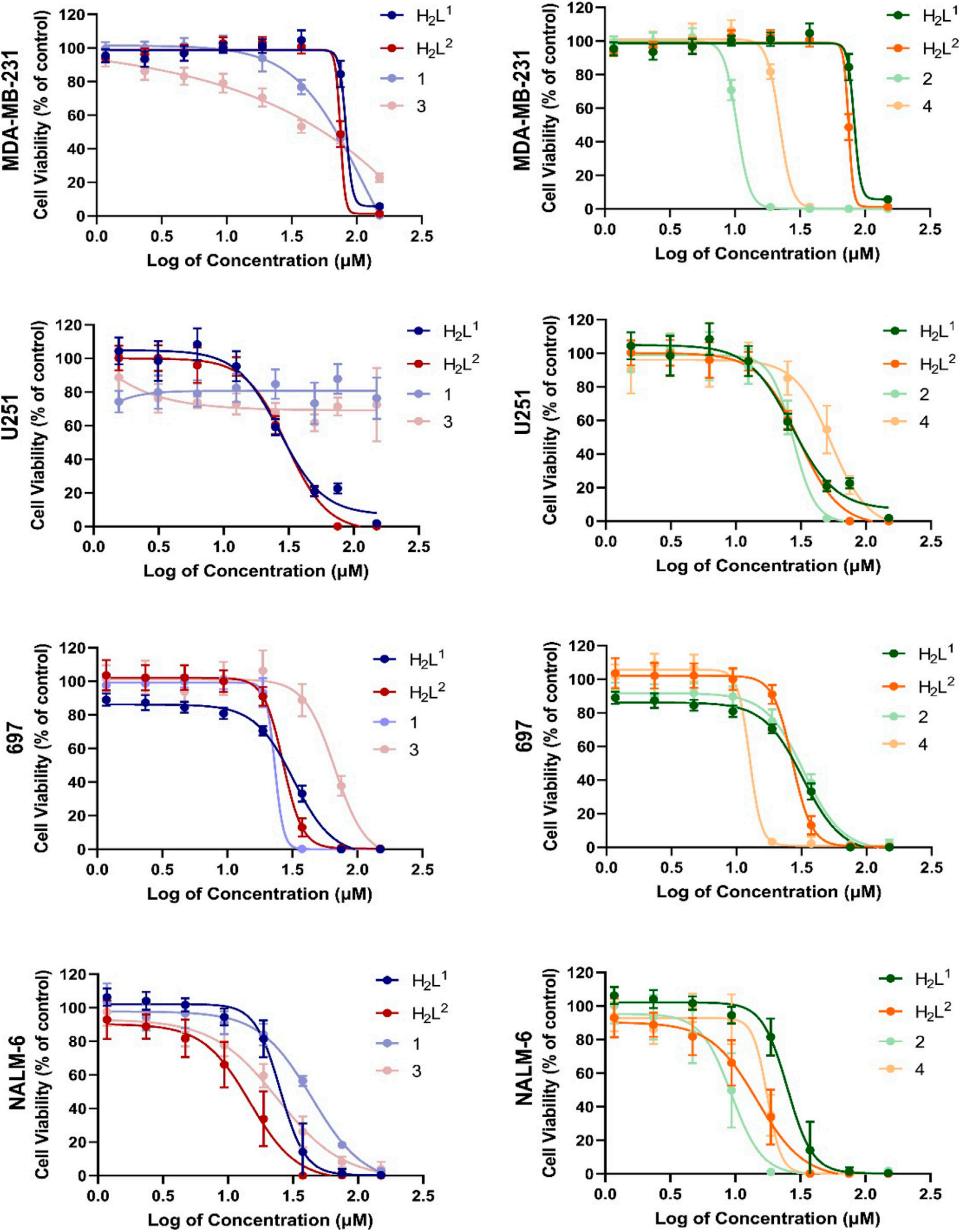
Concentration–response curves of all tested compounds in each cell line after 72 h exposure. Concentrations were shown as logarithms to perform the non-linear regression of data, allowing the calculation of concentration able to inhibit 50% of cell viability in cultures (IC_50%_).

Cancer cells exhibit different biochemical patterns and signaling pathways according to the original tissue (Banti and Hadjikakou, 2016; Chlapek et al., 2017; Oren et al., 2021). If a molecule interacts with a specific protein, for example, it is expected that the biological response reflects the relevance of that protein for cell viability maintenance. In this sense, the H_2_L^1^ (a molecule with a Br atom) showed to be more toxic than the H_2_L^2^ (with NO_2_) at low concentrations for leukemia cells, but not for solid tumor cells (Figure 12).

This graphical approach—comparison among compounds—allowed a preliminary analysis of the relevance of structural features on toxic activity (Figure 12). Except for 697 cells, for which no significant differences were noted, compound (2) was more toxic than compound (1). O the other hand, except for NALM-6, also with no significant differences, compound (4) was more toxic than compound (2). More interestingly, the activities of compounds (1) and (3) were completely abrogated in human glioma U251 cells. These findings indicate that the additional aromatic rings in compounds (1) and (3) decrease their biological activity. There are two main hypotheses to explain this phenomenon. The first one is that this portion of the molecule is relevant for interaction with a target, and the second one is that the increase of the hydrophobic group reduces bioavailability.

Our results showed the potential of the Ni(II) complexes as a prototype of new antitumor drugs. Despite anti-cancer activity already having been demonstrated for Ni(II) complexes which targeted DNA (Zhang et al., 2018) and kinase proteins (Santos et al., 2022), this is not a frequent metal in pharmacological studies (Cu and Zn are much more usual to be found in the specialized literature, for example), making Ni(II) complexes an open field of opportunities to be explored.

### 3.7 Molecular docking simulation

In the attempt to design and better understand how each compound studied in this work, based on the cell type and its IC_50%_ results described below, interact with some proteins expressed in their genome code, more specifically in the cancer cells type, we performed a series of *in silico* simulations through molecular docking methodology. For this purpose, the use of different crystallographic structures for each target cell was taken into account.

Once the IC_50_ values indicate how much the ligand can inhibit cell activity, we can observe that the lowest values indicate the strong affinity between the ligand and the cell type, and consequently the power of inhibition of the cell activity. Considering that the ASP fitness scores are dimensionless, and the values obtained in the docking calculations for this type of algorithm will, in each case, inform the scale of the score that indicates how good the pose is, the higher the score value, the better the docking result is likely to be. In this work, we choose to make an inverse proportional analysis between the IC_50_% values (lowest values) with the ASP fitness values (highest values) to classify and correlate our molecules in this study with their inhibitory function for the protein target.

Table 4 shows the ranked scores of the fitness values (dimensionless) obtained in this study. When compared with the values presented in Table 3, we observed a good agreement in the sequence of inhibition and the score values observed in the docking studies by compound (4) observed in the simulation results when compared with the ligands.

**TABLE 4:** ASP fitness score values (dimensionless) of best-ranked compounds obtained from docking studies of the target enzyme for each cell type.

*Cell line*	*PDBCode*	Compound/ASP fitness					
		H_2_L^1^	H_2_L^2^	(1)	(2)	(3)	(4)
NALM-6	4KCG	13.03	27.78	10.66	30.74	15.93	17.95
697	7DW5	15.72	16.31	16.56	15.33	10.09	17.10
U251	6OQO	18.13	16.82	14.23	27.46	7.44	21.76
MDA-MB-231	6VJ3	20.36	22.16	25.44	34.03	29.59	32.46

Since the protein–receptor target is different for each cell type studied in this work, we will discuss the result of the docking studies separately in different sections to ease the visualization and compression of the interactions performed between the receptors and the six compounds studied here. In the next sections, the active site composition and main residues involved in the catalytic function of the enzymes will be described and discussed, as well as the interactions observed between the receptors and our compounds.

#### 3.7.1 Molecular docking of DCK kinase—NALM-6 cells

**FIGURE 13:**
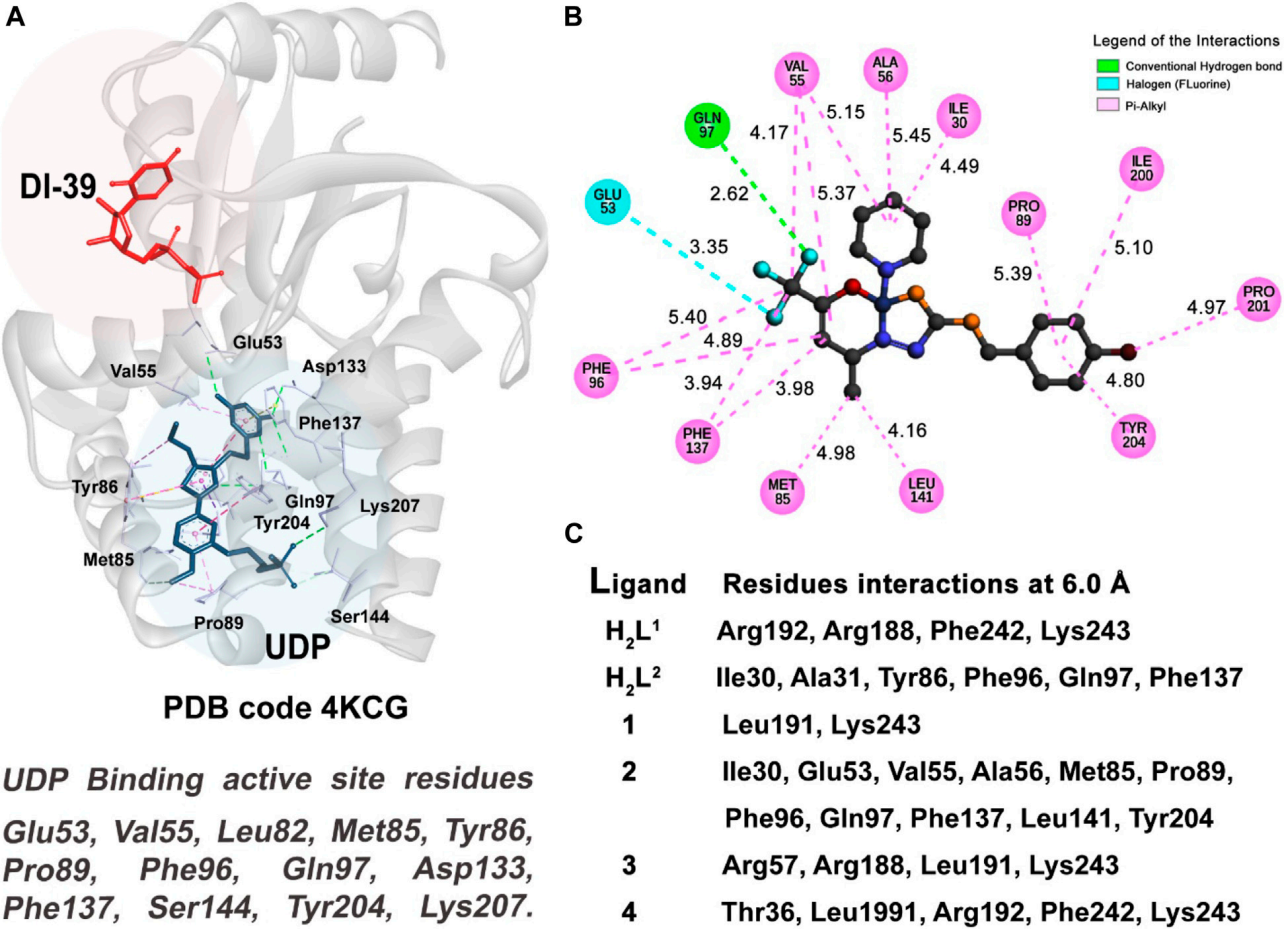
Molecular docking results of NALM-6 target. **(A)** Active site regions of UDP and DI-39 inhibitors and main residues of the DCK protein (**4KCG**) (Nathanson et al., 2014). **(B)** 2D representation of the interactions carried out between the best-ranked inhibitor, **2**, and the UDP active site. **(C)** Main nearest residues of both active sites of the 4KCG receptor (distance till 6.0 Å) and all the six compounds studied.

The active site of this enzyme is divided into two subsites, as shown in Figure 13. For this target, we performed two docking studies: the first study was performed considering the binding active site region located in the same region of the inhibitor DI-39 (in red), and in the second study, we took into account the UDP (in blue) region to select the binding active site. Our results indicate that our six compounds have a better fit and molecular affinity for the active site located in the UDP region, where we could observe more interactions and values of fitness 50% higher than in complexes formed with the residues in the DI-39 ligand.

As can be observed in Table 3 and Table 4, compound (2) and ligand H_2_L^2^ are the best-ranked molecules as better inhibitors in IC_50%_ study and docking studies. When checking in Figure 13C, the residues with which those compounds interact and the region of the interactions, it is clear that the interactions with the residues of the UDP active site are more efficient in making the inhibitor complex more stable. The interactions with the residue Gln97 are mainly strong hydrogen bond type, and in the case of the UDP molecule, this interaction occurs at a distance of 2.96 Å. Compound 2 has established this interaction at the distance of 2.62 Å, (see Figure 13B) and the ligand H_2_L^2^ performs the same interaction at 2.97 Å (not shown).

The pi-alkyl van der Waals type (in pink color in Figure 13B) interactions observed between the receptor and the UDP molecule (Supplementary Figure S33), compound (2) (shown in Figure 13B), and with the residues, Val55, Met85, Pro89, Phe96, Phe137, and Tyr204, are also good indicators to a better performance for a good inhibitor of this enzyme.

#### 3.7.2 Molecular docking of DUX4_1-150_-DNA_ERG_ HD1-HD2—697 cells

The DNA-binding double homeobox 4 fused with immunoglobulin domain 1 and 2 (DUX4_1-150_-DNA_ERG_ HD1-HD2) protein complexed with the expression recombinant gene (ERG_ALT_) has its catalytic activity related to the interaction between the residues of the Linker A and Linker B of the DUX4_1-150_ protein (Zhang et al., 2021). The mechanism of oncogenic biogenesis of ERG_alt_ is activated when the atom OE2 of the residue Glu93 of Linker A establishes a strong hydrogen bond (around 2.20 Å) with the atom ND2 of the residue Asn41 of Linker B.

**FIGURE 14:**
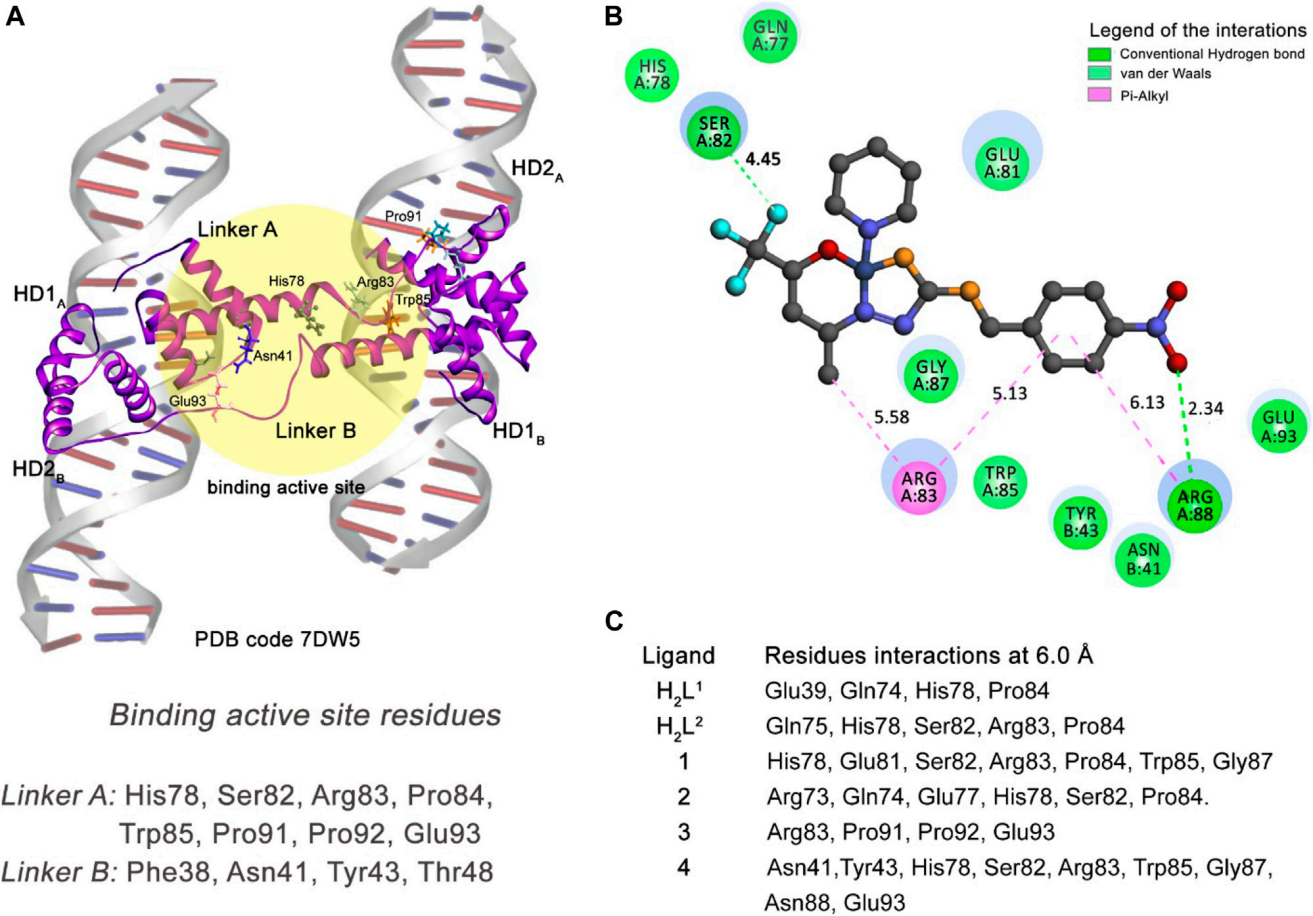
Molecular docking results of the 697 cell type target. **(A)** Binding active site regions of the Linkers A and B of the protein–DNA complex (**7DW5**) (Zhang et al., 2021). **(B)** 2D representation of the interactions carried out between the best-ranked inhibitor, **4,** and the active site. **(C)** Main nearest residues of both active sites of the **7DW5** receptor (distance till 6.0 Å) and all the six compounds studied.

A good strategy to avoid this interaction is the design of molecules able to interact with both linkers in the region where the residues Glu93 (Linker A) and Asn41 (Linker B) are located. Thus, by inhibiting the junction of the two monomers of the HD1 and HD2 proteins, the oncobiogenesis pathway of the ERG_alt_ can be blocked. We performed a docking study considering the linker junction of the DUX4_1-150_-DNA_ERG_ HD1-HD2 residues as the target receptor (see Figure 14A) and our six proposed compounds.

Our docking simulation reveals that compound (4) can interact *via* medium-range van der Waals (distance till 6.0 Å) interactions with both Asn41 (Linker B) and Glu93 (Linker A) residues (Figures 14B,C). In addition, this compound makes strong hydrogen bond interactions with Arg88 (Linker A) residue and other residues of the Linker A monomer through van der Waals and pi–alkyl interactions. This compound is the better inhibitor in our series of molecules for the 697-cell type. The other five compounds presented interactions only with residues of the Linker A monomer. This can explain their less inhibitory power when compared with the compound (4).

#### 3.7.3 Molecular docking of cyclic-dependent kinase 6 (CDK6)—U251 cells

The CDK6 crystallographic structure (6OQO) was taken as the enzyme to represent the U251 cell in this work. The main function of CDK6 is the regulation of the cell cycle through the phosphorylation of the tumor suppressor retinoblastoma protein (Bronner et al., 2019). The design of a specific inhibitor of the CDK6 is a therapeutic target for cancer treatment.

According to the values shown in Table 3, of IC_50%_, compound (2), followed by the ligands H_2_L^1^ and H_2_L^2^, are the best-ranked inhibitors of U251 cells. However, following the values presented in Table 4, the score of the ASP fitness, we can observe that compound (4) is the second most potent inhibitor instead of the ligands H_2_L^1^ and H_2_L^2^.

**FIGURE 15:**
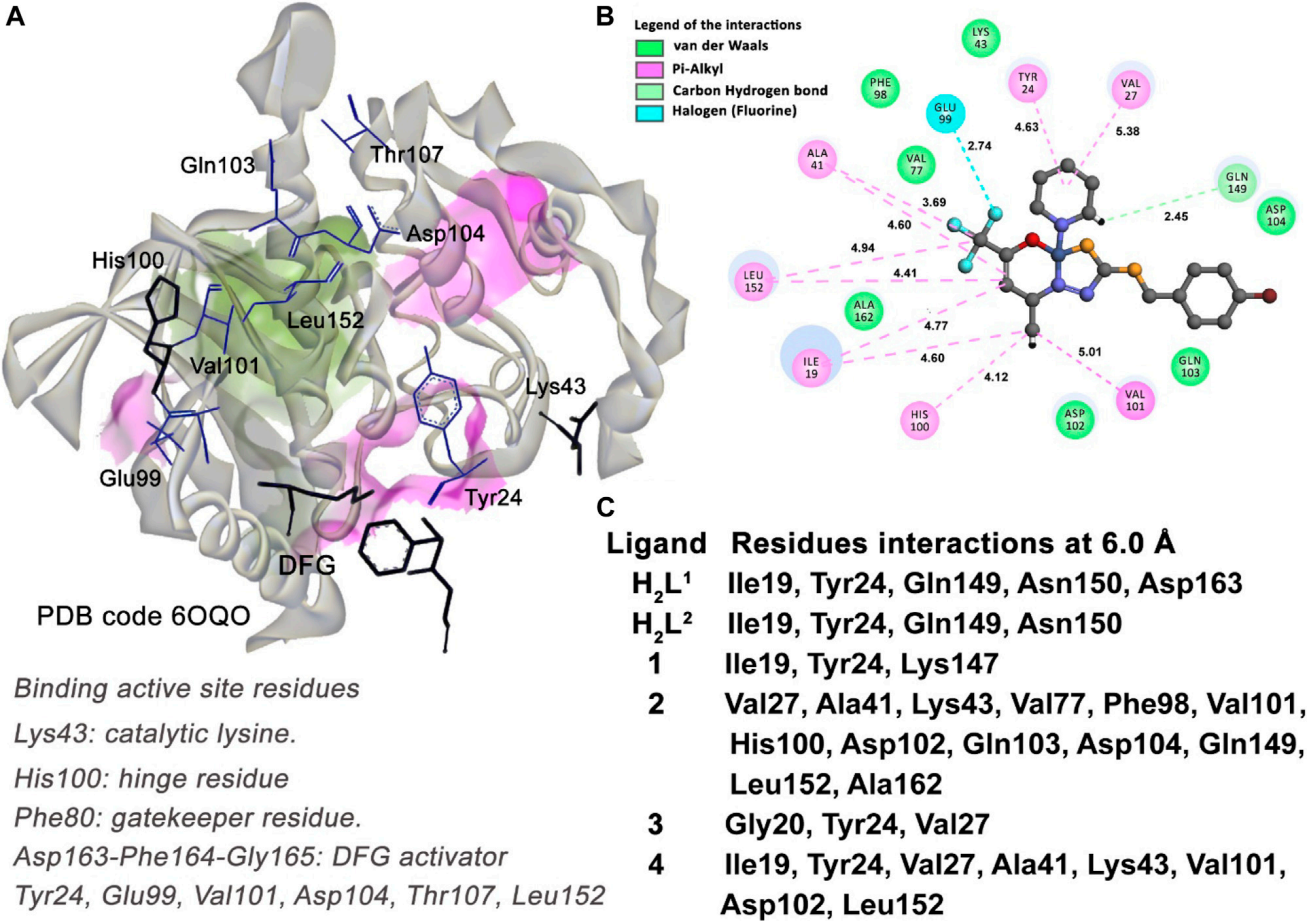
Molecular docking results of the U251 cell type target. **(A)** Binding active site regions of the CDK6 kinase protein (**6OQO**) (Bronner et al., 2019), the DFG catalytic triad, and the gatekeeper residue His100 are also represented. **(B)** 2D representation of the interactions carried out between the best-ranked inhibitor, **2,** and the active site. **(C)** Main nearest residues of both active sites of the **6OQO** receptor (distance till 6.0 Å) and all the six compounds studied.

Taking into account the residues with which the molecules in the study interact, as shown in Figure 15C, compounds (2) and (4) are the inhibitors that have more interactions with the binding active site residues. They interact with the Lys43 residue, the catalytic lysine of the CDK6 kinase, forming a medium-range van der Waals interaction. The compound (2) is the most potent inhibitor for CDK6 enzyme among the molecules, ranked due to the number of interactions with the residues of the binding active site of the protein. Among these, it is important to highlight the pi–alkyl interaction with the gatekeeper residue His100 (4.12 Å) and the strong halogen (fluorine) interaction with the Glu99 residue (2.74 Å).

#### 3.7.4 Molecular docking of human carbonic anhydrase—MDA-MB-231 cells

The inhibition of the human carbonic anhydrase (CA) enzyme is key to avoiding the acidification of the extracellular cells in several types of tumors in the crystallographic structure of CA protein, deposited in the Protein Data Bank under the code 6VJ3 complexed with a pyrimidine-based inhibitor named QYA.

The inhibitor shows strong hydrogen bond interactions with some residues located in the active site of the CA enzyme. The hydrogen bond interactions between the atoms of the ligand QYA and the residues Asn67 (3.03 Å), Gln92 (2.04 Å), Leu198 (2.48 Å), Thr199 (2.23 Å), and Thr200 (2.50 Å) were identified. Other interactions with key residues such as the Phe131 (cation–pi and pi–pi T-shaped interactions) and Val121 (pi–alkyl interaction) and a strong coordinative covalent bond with the tetracoordinate Zn ion (1.93 Å) are indicative interactions for the specific interaction profile of an efficient inhibitor (Petreni et al., 2020). For a molecule to be considered a good candidate inhibitor for the CA enzyme, it is necessary to establish indicative interactions, as described previously, with the key residues of the active site.

**FIGURE 16:**
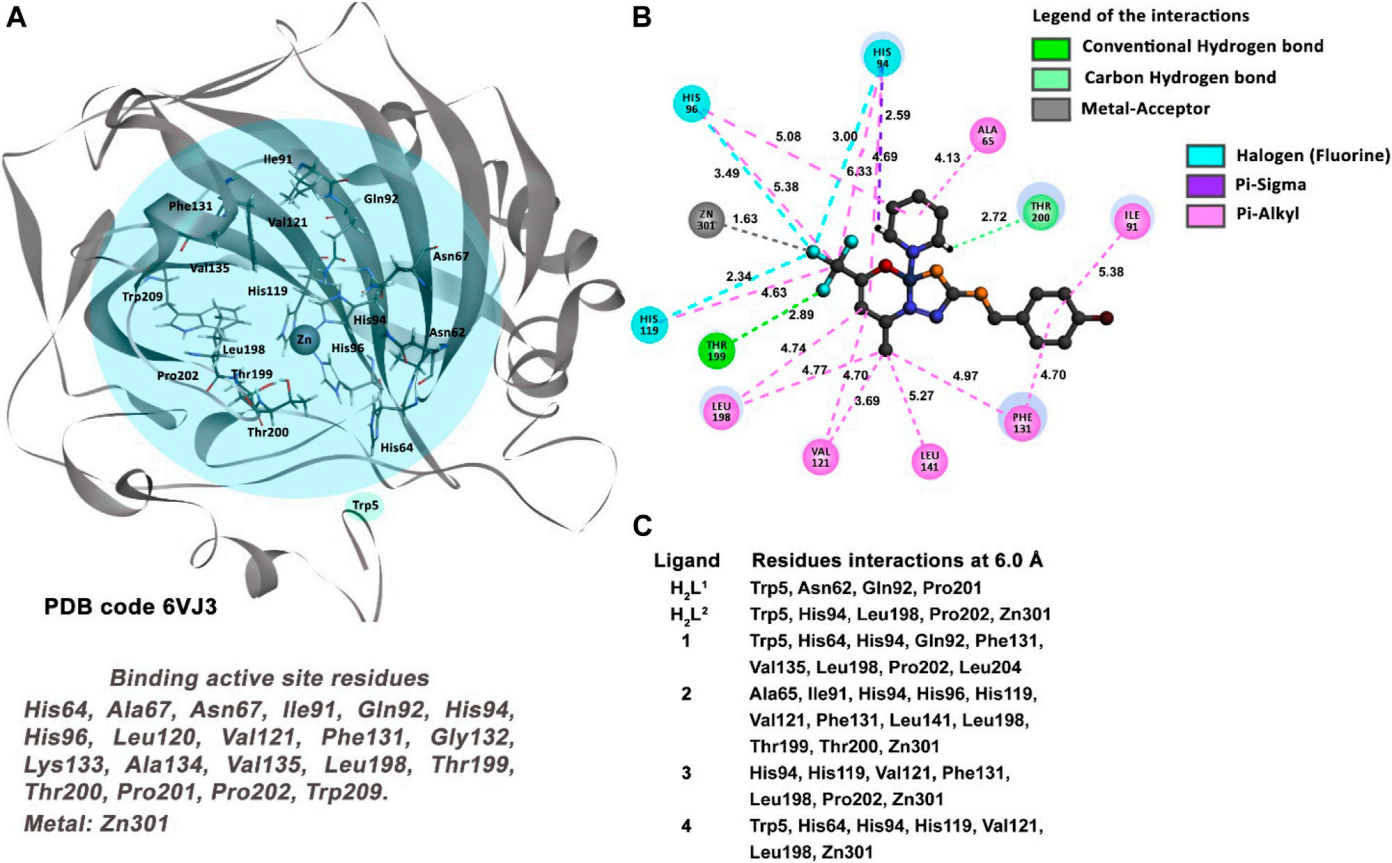
Molecular docking results of the MDA-MB-231 cell type target. **(A)** Binding active site regions of the CA protein (**6VJ3**) (Petreni et al., 2020). **(B)** 2D representation of the interactions carried out between the best-ranked inhibitor, **2,** and the residues of the active site of the receptor. **(C)** Main nearest residues of both active sites of the **6VJ3** receptor (distance till 6.0 Å) and all the six compounds studied.

The docking study between the CA and the six molecules proposed reveals compound (2) as the best-ranked ligand as an inhibitor (Table 4). As described in Figure 16C, all molecules interact with some residues of the active site of the protein. However, compounds (2), (3), and (4) have interactions with key residues, such as the Val121, Leu198, and the metal ion Zn. The metal-acceptor-type interactions with the Zn ion and the compounds (2), (3), and (4) were observed at distances of 2.72 Å, 1.76 Å, and 3.32 Å, respectively.

On the other hand, compound (2) is the unique candidate to interact with the residues Thr199 (2.23 Å) and Thr200 (2.50 Å), with strong hydrogen bond interactions. These interactions give this compound a better profile of inhibition compared with the other five molecules in this study.

## 4 Conclusion

Two dithiocarbazate ligands and four new Ni(II) complexes were synthesized, and their structures were investigated by various analytical approaches. The crystal structure of the H_2_L^1^ ligand revealed a cyclic compound in the solid state, and the single-crystal X-ray diffraction data of the Ni(II) complexes showed the dithiocarbazate ligands coordinated to the metal center by the *ONS* donor atoms. Moreover, the square planar coordination geometry is completed by a triphenylphosphine or pyridine molecule. The Hirshfeld surface analysis allowed the evaluation of the topography of intermolecular interactions and quantitative data on the contacts that most contribute to the formation of the crystal lattice. The mass spectrometry analysis revealed the presence of molecular ions [M + H]^+^ and characteristic fragmentations of the compounds. All compounds showed biological activity in the *in vitro* screening. Most importantly, the effects had different intensities and behaviors according to the tumor cell line used, pointing to a target-specific mechanism of action. The results also showed that some structural variations can modify the biological activity, which highlights the next steps for eventual pharmacologic studies with these prototypes. The molecular docking simulation showed a good agreement in the sequence of inhibition and the score values observed in the docking studies. According to the docking results, compounds (2) and (4) show the best performance as complexes for a good inhibitor, considering all the enzymes studied. The other molecules studied showed cell type-dependent performance in inhibitory activity.

## Data Availability

The original contributions presented in the study are included in the article/Supplementary Materials; further inquiries can be directed to the corresponding author.
