# Supplementary material for: Dithiocarbazate ligands and their Ni(II) complexes with potential biological activity: Structural, antitumor and molecular docking study
Source: Front Mol Biosci. 2023 Mar 7;10:1146820. doi: 10.3389/fmolb.2023.1146820 (PMC10034969; doi:10.3389/fmolb.2023.1146820)
Supplement: Supplementary file 3 [file DataSheet6.PDF]

Dithiocarbazate ligand-based Ni(II) complexes: Synthesis, structural investigations, solution behavior, antitumor activity and molecular docking studies

Supplementary Material

Summary:

Table S1. Angular strain and strain (cm^{-1}) frequencies of the normal vibration modes selected for all compounds studied. 3

Table S2. ^{1}H-NMR data for H_2L^1. 4

Table S3. ^{1}H-NMR data for H_2L^2. 5

Table S4. ^{1}H-NMR data for (1). 6

Table S5. ^{1}H-NMR data for (2). 6

Table S6. ^{1}H-NMR data for (3). 7

Table S7. ^{1}H-NMR data for (4). 7

Table S8. Results obtained in the electron spectroscopy with wavelength values in nm. 8

Figure S1. Projection view of (1) showing the hydrogen bonds present. 9

Figure S2. Projection view of (2) showing the hydrogen bonds present. 9

Figure S3. Projection view of (3) showing the hydrogen bonds present. 10

Figure S4. Projection view of (4) showing the hydrogen bonds present. 10

Figure S5. Hirshfeld surface of H_2L^1 mapped with d_{norm}. 11

Figure S6. Fingerprint plots for H_2L^1. 11

Figure S7. Fingerprint plots for (1). 12

Figure S8. Fingerprint plots for (2). 13

Figure S9. Fingerprint plots for (3). 14

Figure S10. Fingerprint plots for (4). 15

Figure S11. IR spectra of H_2L^1. 16

Figure S12. IR spectra of H_2L^2. 16

Figure S13. IR spectra of compound (1). 16
Figure S14. IR spectra of compound (2). 17
Figure S15. IR spectra of compound (3). 17
Figure S16. IR spectra of compound (4). 17
Figure S17. ESI(+)-MS of H_2L^1 and H_2L^2. 18
Figure S18. ESI(+)-MS of (1)-(4). 18
Figure S19. ESI(+)-MSMS of H_2L^2. 19
Figure S20. ESI(+)-MSMS of (3)......19
Figure S21. ESI(+)-MSMS of (4)......20
Figure S22. ^{1}H-NMR spectra of compound H_2L^1. 20
Figure S23. ^{1}H-NMR spectra of compound H_2L^2. 21
Figure S24. ^{1}H-NMR spectra of compound (1)......21
Figure S25. ^{1}H-NMR spectra of compound (2)......22
Figure S26. ^{1}H-NMR spectra of compound (3)......22
Figure S27. ^{1}H-NMR spectra of compound (4)......23
Figure S28. UV-vis spectra of compound H_2L^1, (1) and (2) in MeOH. 23
Figure S29. UV-vis spectra of compound H_2L^1, (1) and (2) in DMSO. 24
Figure S30. UV-vis spectra of compound H_2L^2, (3) and (4) in MeOH. 24
Figure S31. UV-vis spectra of compound H_2L^2, (3) and (4) in DMSO. 25
Figure S32. Superposition between the 2D crystallographic structure and 2D redocking (best pose) of the known compounds UDP (Inhibitor of the NALM-6 cell), QYA (inhibitor of MDA-MB-231 cell), and NIJ (Inhibitor of U251 cell) obtained during the validation of docking protocol. The Figure is also shown the values of the RMSD and the ASP fitness score……26

Figure S33. Main interactions and respective distances between the residues of the active site and known inhibitors. For (A) interactions between the UDP inhibitor and the human deoxycytidine kinase active site residues; (B) main interactions between the NIJ classical inhibitor and the CDK6 kinase binding site residues; (C) interactions between the QYA inhibitor and the human carbonyl anhydrase residues……27

Table S1. Angular strain and strain (cm^{-1}) frequencies of the normal vibration modes selected for all compounds studied.

	H_2L^1	H_2L^2	(1)	(2)	(3)	(4)
**ν(C=S)**	1300	1310	-	-	-	-
**ν(C–S)**	734	732	745	760	747	759
**ν(O–H)**	3199	3151	-	-	-	-
**ν(N–N)**	1113	1113	1096	1070	1096	1068
**ν(C=N)**	1643	1636	1599	1606	1598	1604
**ν(C–F)**	1200-1137	1205-1138	1182-1137	1192-1114	1179-1139	1190-1113
**ν(C=C)**	1588	1596	1529	1528	*	*
	1486	1446	1483	1485	1479	1486
**ν(NO_2)asy**	-	1515	-	-	1515	1516
**ν(NO_2)sym**	-	1344	-	-	1342	1342
**δ(Py)**	-	-	-	689	-	689
**ν(Ni–PPh_3)**	-	-	693	-	693	-

Legend: (-) does not apply and (*) band not observed.

Table S2. ^{1}H-NMR data for H_2L^1.

δ (ppm)	Multiplicity	Integral	*J* (Hz)	Atribuition
2.05	s	3	-	5 (C**H**$_3$)
3.32	d	1	$^{2}J_{H3a\text{-}H3b}$ = 19.81 Hz	3a (C**H**$_2$)
3.64	d	1	$^{2}J_{H3a\text{-}H3b}$ = 19.81 Hz	3b (C**H**$_2$)
4.30	d	1	$^{2}J_{H7a\text{-}H7b}$ = 13.94 Hz	7b (S-C**H**$_2$)
4.35	d	1	$^{2}J_{H7a\text{-}H7b}$ = 13.94 Hz	7a (S-C**H**$_2$)
7.34	d	2	$^{3}J_{H9\text{-}H10}$ = 8.44 Hz	9 (–C$\mathbf{H}^{9}_{Ar}$=)
7.51	d	2	$^{3}J_{H9\text{-}H10}$ = 8.44 Hz	10 (–C$\mathbf{H}^{10}_{Ar}$=)
8.26	s	1	-	1a (O-**H**)

Table S3. ^{1}H-NMR data for H_2L^2.

δ (ppm)	Multiplicity	Integral	*J* (Hz)	Atribuition
2.06	d	3	-	5 (C**H₃**)
3.32	d	1	$^2J_{H3a\text{-}H3b}$ = 19.81 Hz	3a (C**H₂**)
3.65	d	1	$^2J_{H3a\text{-}H3b}$ = 19.81 Hz	3b (C**H₂**)
4.49	d	1	$^2J_{H7a\text{-}H7b}$ = 14.31 Hz	7b (S-C**H₂**)
4.55	d	1	$^2J_{H7a\text{-}H7b}$ = 14.31 Hz	7a (S-C**H₂**)
7.65	d	2	$^3J_{H9\text{-}H10}$ = 8.80 Hz	9 (–C$\mathbf{H}^9_{Ar}$=)
8.18	d	2	$^3J_{H9\text{-}H10}$ = 8.80 Hz	10 (–C$\mathbf{H}^{10}_{Ar}$=)
8.33	s	1	-	1a (O-**H**)

Table S4. ^{1}H-NMR data for (1).

δ (ppm)	Multiplicity	Integral	*J* (Hz)	Atribuition
2.41	S	3	-	5 (C**H**$_3$)
4.28	s	2	-	7 (S-C**H**$_2$)
5.89	s	1	-	3 (C**H**)
7.31	d	2	$^3J_{H9-H10}$ = 8.44 Hz	9 (–C**H**$^9_{Ar}$=)
7.44-7.33	m	17	-	10 (–C**H**$^{10}_{Ar}$=) e H (PPh$_3$)

Table S5. ^{1}H-NMR data for (2).

δ (ppm)	Multiplicity	Integral	*J* (Hz)	Atribuition
14..4	S	3	-	5 (C**H**$_3$)
4.03	s	2	-	7 (S-C**H**$_2$)
4.89	s	1		3 (C**H**)
7.34	d	2	$^3J_{H9-H10}$ = 8.07 Hz	9 (–C**H**$^9_{Ar}$=)

7.52	d	2	$^{3}J_{H9\text{-}H10}$ = 8.07 Hz	10 (–C**H**^{10}$_{Ar}$=)
8.12-7.97	m	3	-	13 e 14 (Py)
10.46	s	2	-	12 (Py)

Table S6. ^{1}H-NMR data for (3).

δ (ppm)	Multiplicity	Integral	*J* (Hz)	Atribuition
**2.39**	s	3	-	5 (C**H**$_3$)
**4.43**	s	2	-	7 (S-C**H**$_2$)
**5.58**	s	1	-	3 (C**H**)
**7.44-7.73**	m	17	-	9 (–C**H**^{9}$_{Ar}$=) e H (PPh$_3$)
**8.18**	d	2	$^{3}J_{H9\text{-}H10}$ = 8.44 Hz	10 (–C**H**^{10}$_{Ar}$=)

Table S7. ^{1}H-NMR data for (4).

δ (ppm)	Multiplicity	Integral	*J* (Hz)	Atribuition
1.23	s	3	-	5 (C**H**$_3$)
4.09	s	2	-	7 (S-C**H**$_2$)

4.71	s	1		3 (C**H**)
7.67	d	2	$^{3}J_{H9\text{-}H10}$ = 8.44 Hz	9 (–C**H**^{9}$_{Ar}$=)
8.13	m	1	-	14 (Py)
8.21	d	2	$^{3}J_{H9\text{-}H10}$ = 8.44 Hz	10 (–C**H**^{10}$_{Ar}$=)
8.27	s	2	-	13 (Py)
11.14	s	2	-	12 (Py)

Table S8. Results obtained in the electron spectroscopy with wavelength values in nm.

		**π – π*ᵃ**	**Log ε**	**n – π*ᵃ**	**Log ε**	**LMCT ᵃ**	**Log ε**
H_2L^1	MeOH	238 and 297	4.25 and 3.99	389	4.20	-	-
	DMSO	258 and 302	4.12 and 4.01	396	4.29	-	-
H_2L^2	MeOH	275	4.33	-	-	-	-
	DMSO	284	4.32	-	-	-	-
(1)	MeOH	237 and 262	4.69 and 4.55	318	4.06	369	4.12
	DMSO	265	4.44	-	-	380	3.95
(2)	MeOH	246 and 259	4.48 and 4.45	310	3.97	372	4.10
	DMSO	265	4.34	-	-	281	4.03
(3)	MeOH	241 and 268	4.67 and 4.63	-	-	368	4.15
	DMSO	271	4.39	-	-	378	3.84
(4)	MeOH	264	4.24	-	-	371	3.79
	DMSO	270	4.20	-	-	380	3.84

a) Absorption band values in nm.

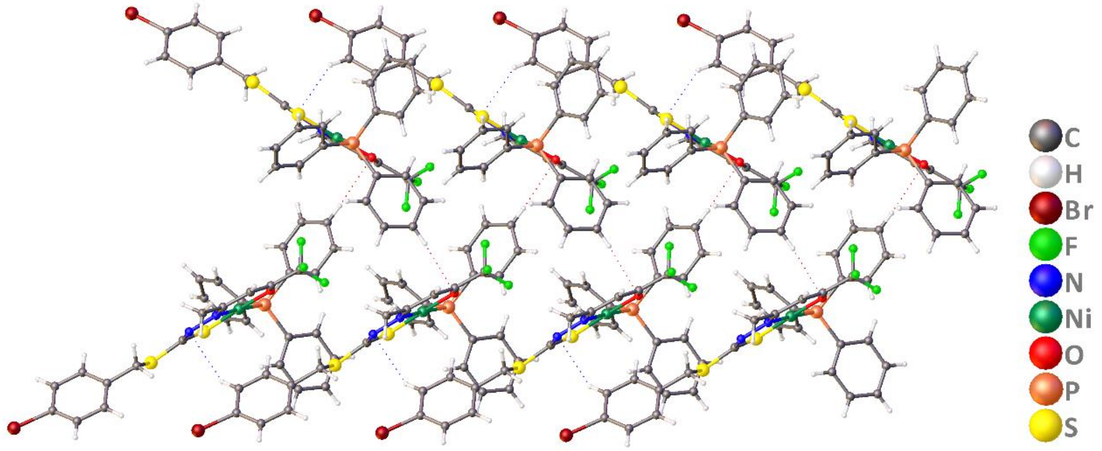

Figure S1. Projection view of (1) showing the hydrogen bonds present.

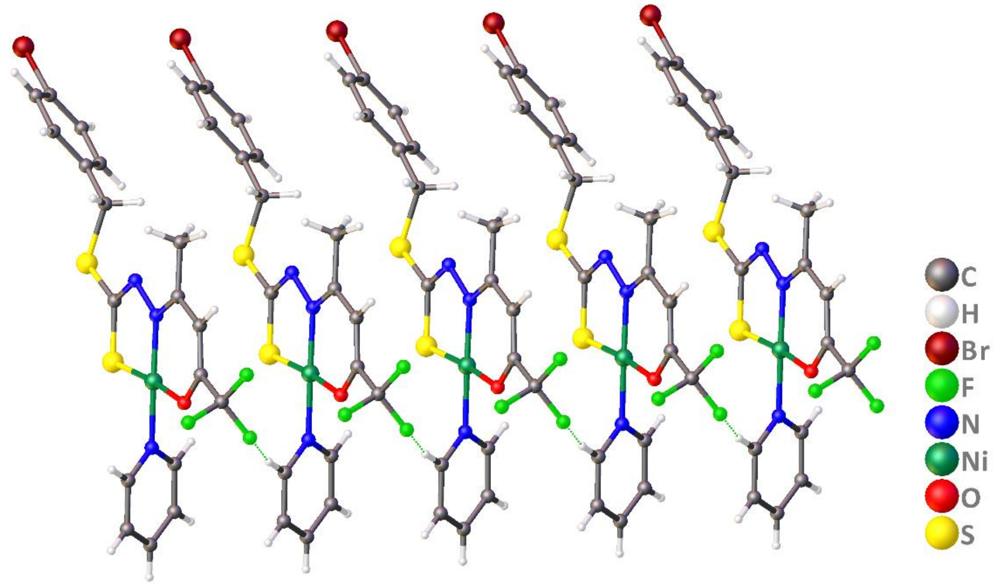

Figure S2. Projection view of (2) showing the hydrogen bonds present.

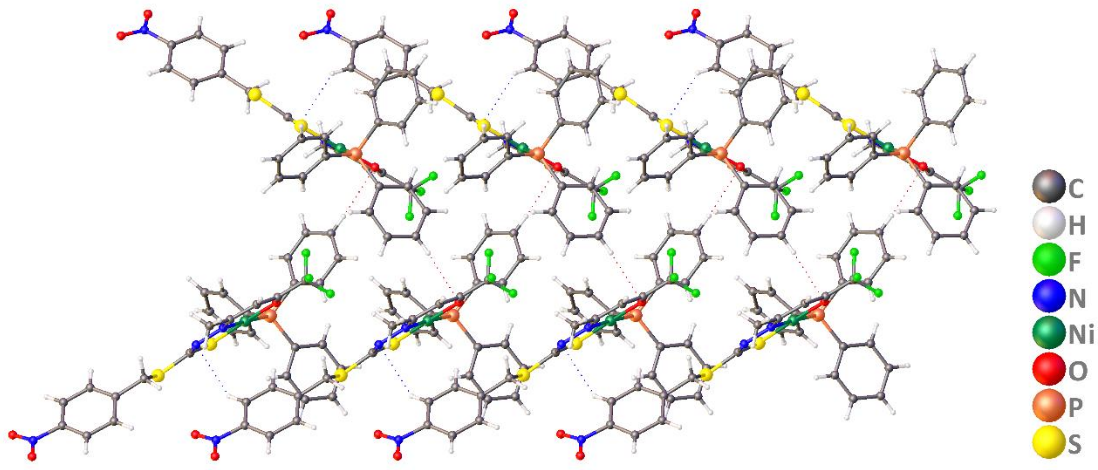

Figure S3. Projection view of (3) showing the hydrogen bonds present.

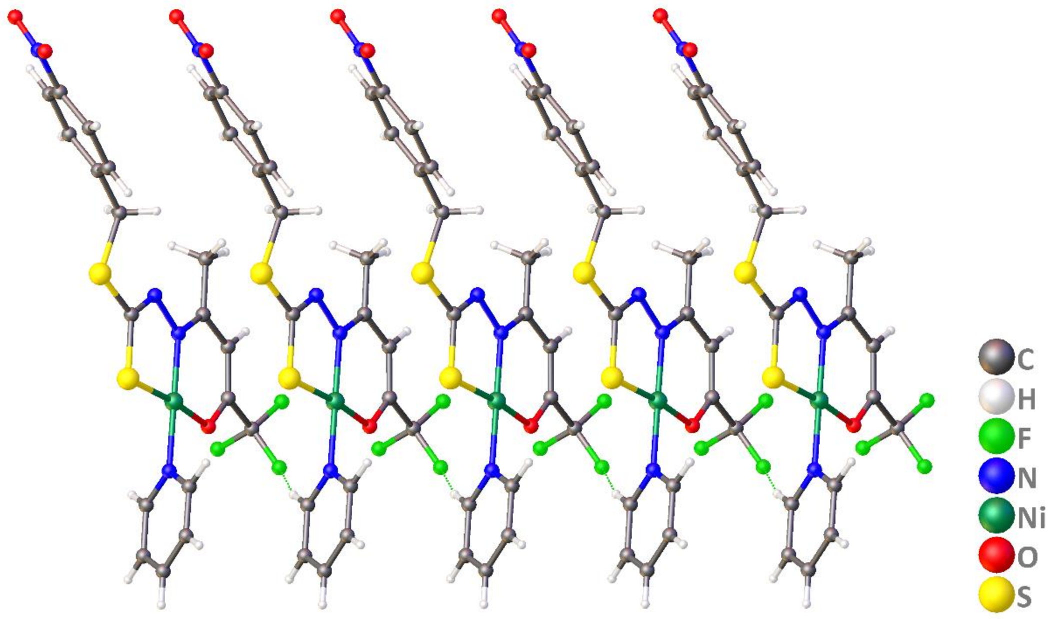

Figure S4. Projection view of (4) showing the hydrogen bonds present.

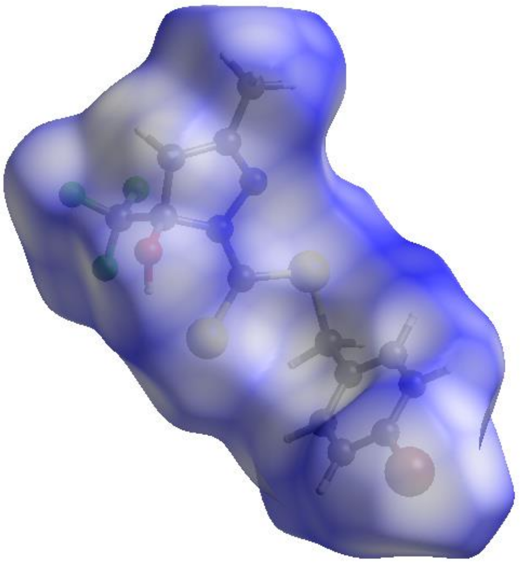

Figure S5. Hirshfeld surface of H_2L^1 mapped with d_{norm}.

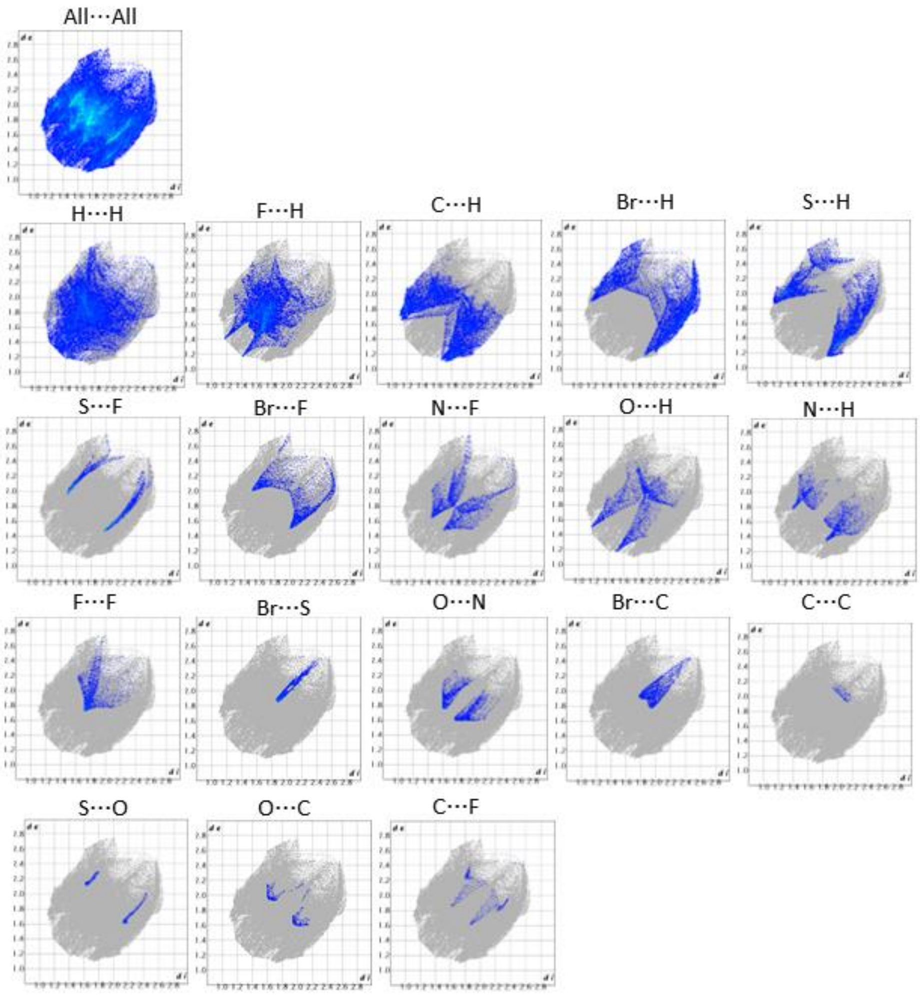

	H_2L^1 % contribution
H-H	25.5
F-H	15.9
C-H	13.3
Br-H	11.4
S-H	9.4
S-F	3.4
Br-F	3.1
N-F	3.1
O-H	2.8
N-H	2.1
F-F	2.1
Br-S	1.7
O-N	1.7
Br-C	1.5
C-C	1.5
S-O	0.7
O-C	0.4
C-F	0.4

Figure S6. Fingerprint plots for H_2L^1.

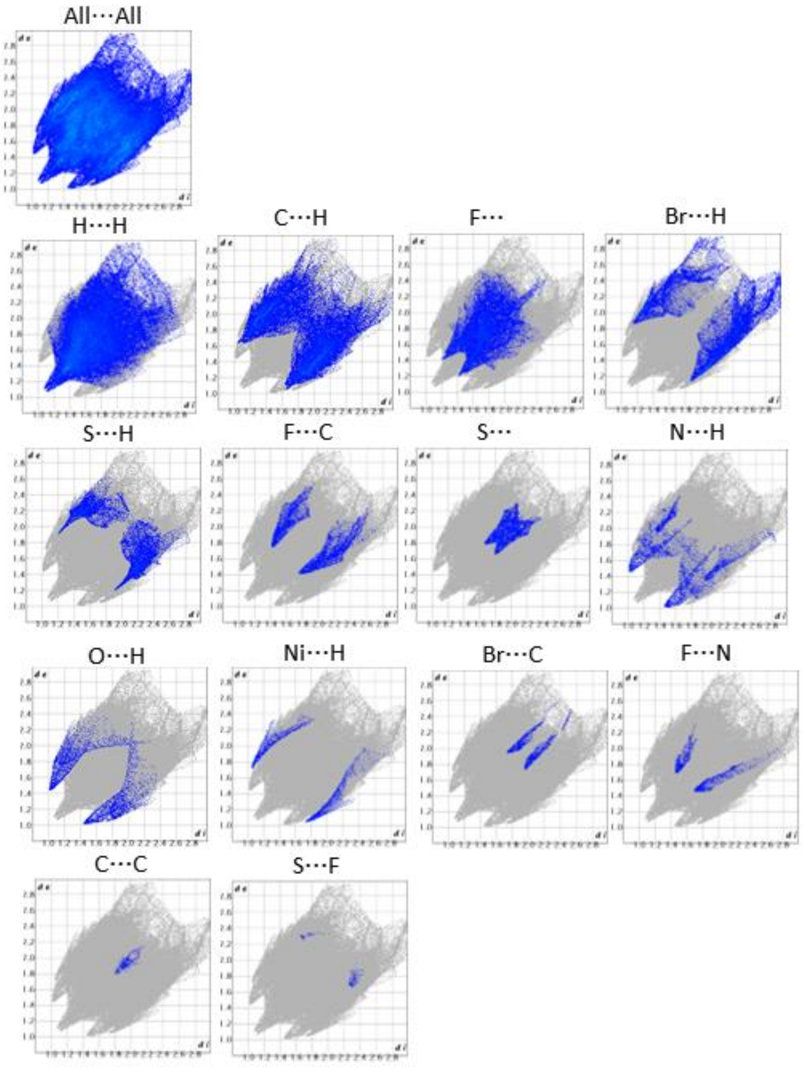

	(1)<br>% contribution
H-H	38.2
C-H	20.9
F-H	11.8
Br-H	9.5
S-H	6.0
F-C	3.2
S-C	2.6
N-H	2.6
O-H	2.1
Ni-H	1.2
Br-C	0.8
F-N	0.8
C-C	0.2
S-F	0.1

Figure S7. Fingerprint plots for (1).

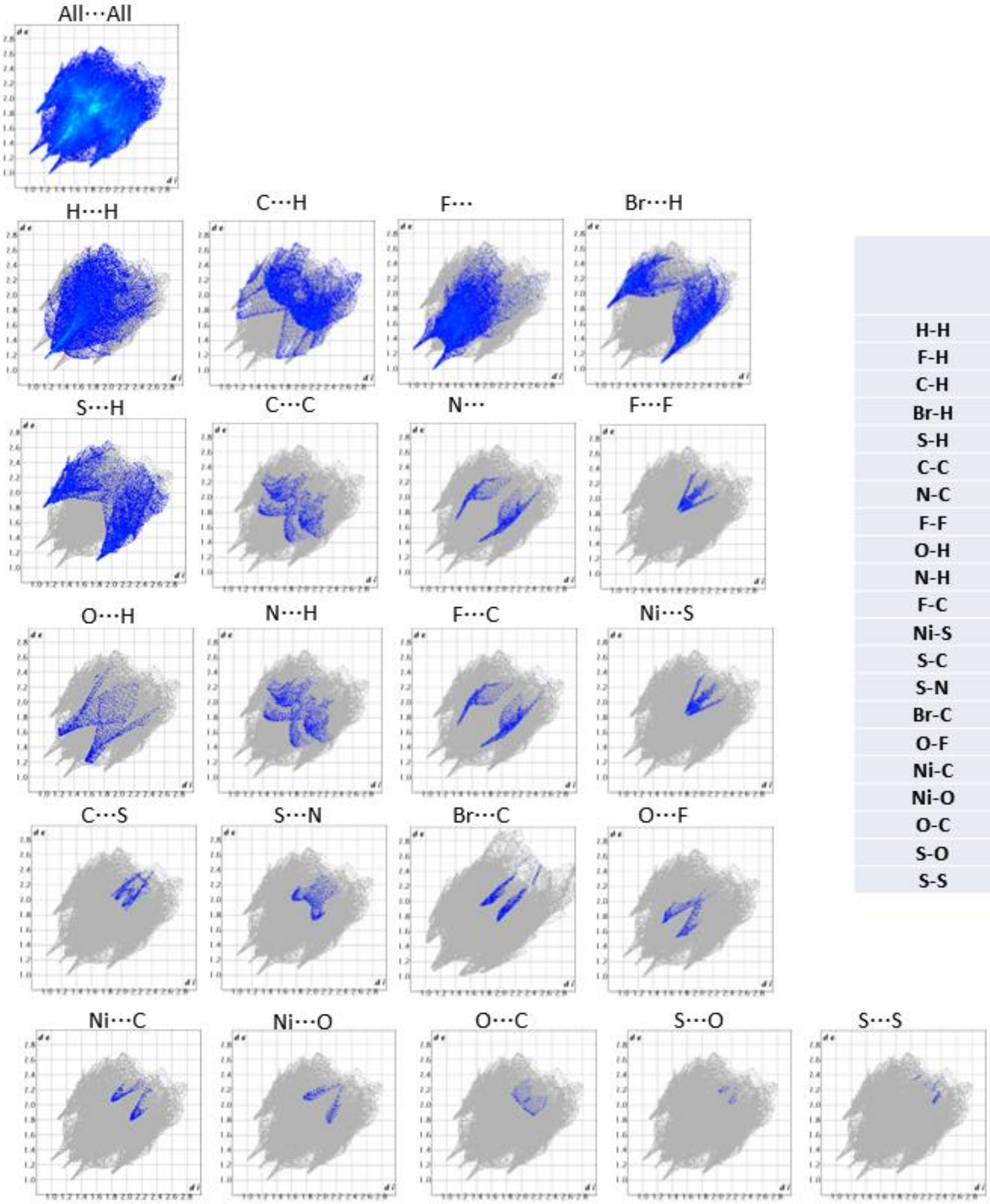

	(2)<br>% contribution
H-H	25.6
F-H	17.8
C-H	13
Br-H	12
S-H	11.9
C-C	3.0
N-C	2.6
F-F	2.3
O-H	1.9
N-H	1.9
F-C	1.8
Ni-S	1.1
S-C	1.0
S-N	1
Br-C	0.8
O-F	0.7
Ni-C	0.6
Ni-O	0.4
O-C	0.3
S-O	0.1
S-S	0.1

Figure S8. Fingerprint plots for (2).

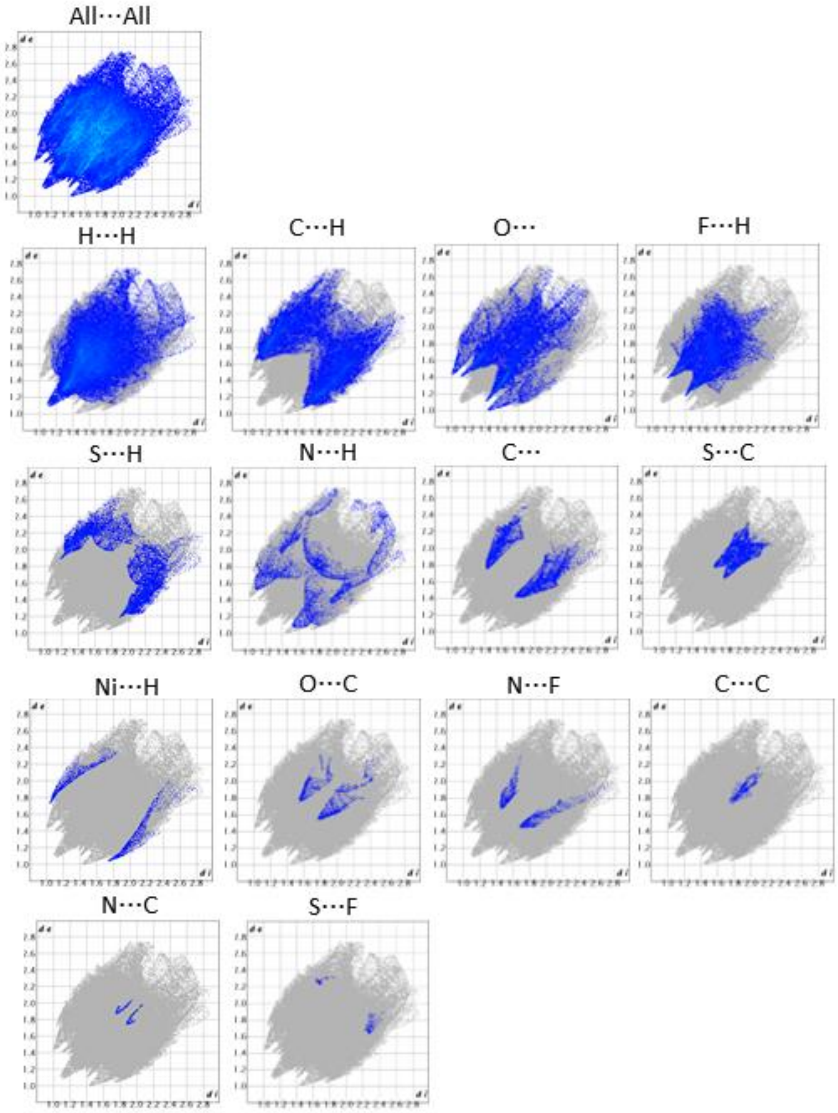

	(3) % contribution
H-H	34.2
C-H	20.6
O-H	14.6
F-H	11.8
S-H	6.0
N-H	3.5
C-F	3.1
S-C	2.7
Ni-H	1.1
O-C	0.8
N-F	0.8
C-C	0.3
N-C	0.3
S-F	0.2

Figure S9. Fingerprint plots for (3).

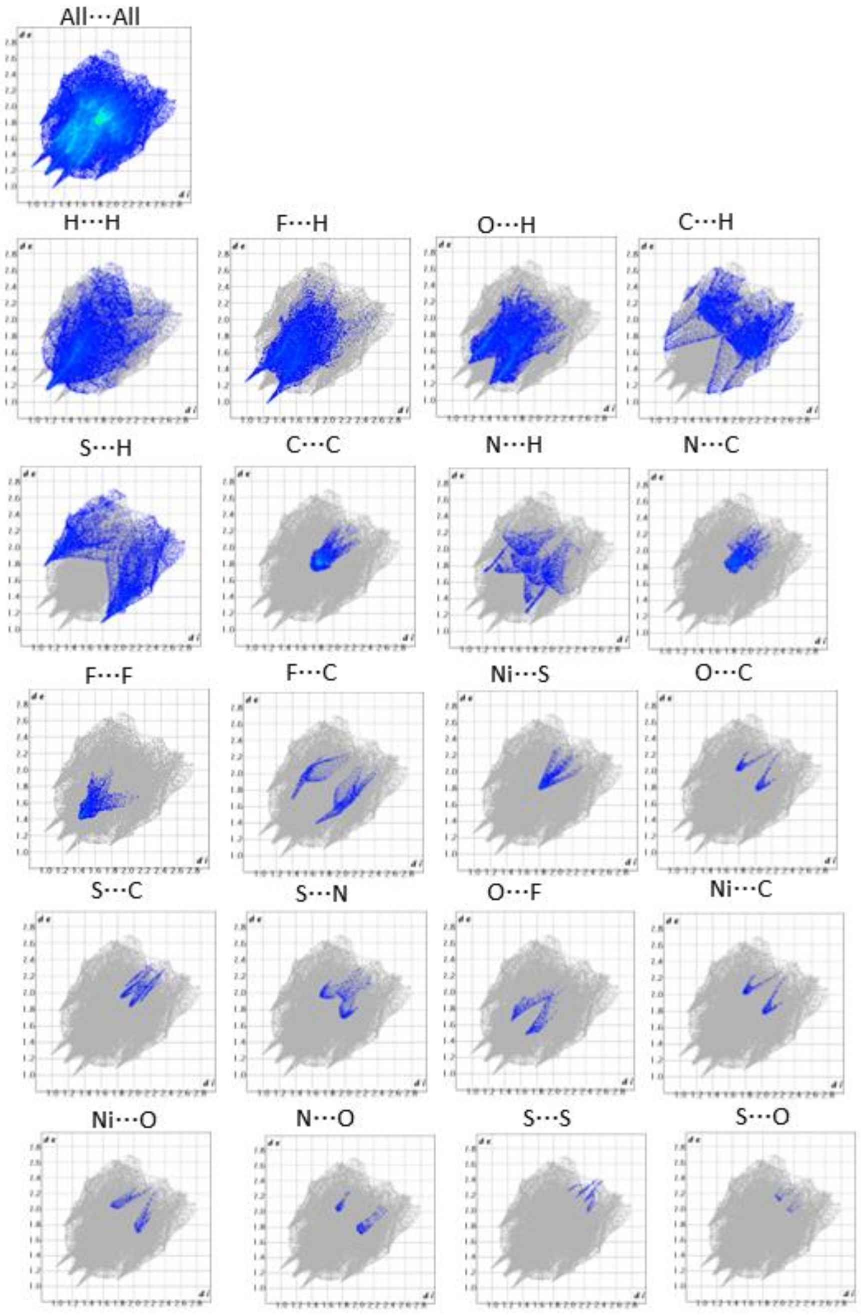

	(4) % contribution
H-H	21.7
F-H	17.5
O-H	17
C-H	11.7
S-H	11.4
C-C	3.4
N-H	3.1
N-C	3.1
F-F	2.3
F-C	1.7
Ni-S	1.2
O-C	1.2
S-C	1
S-N	0.9
F-O	0.8
Ni-C	0.5
Ni-O	0.5
O-N	0.3
S-S	0.2
S-O	0.1

Figure S10. Fingerprint plots for (4).

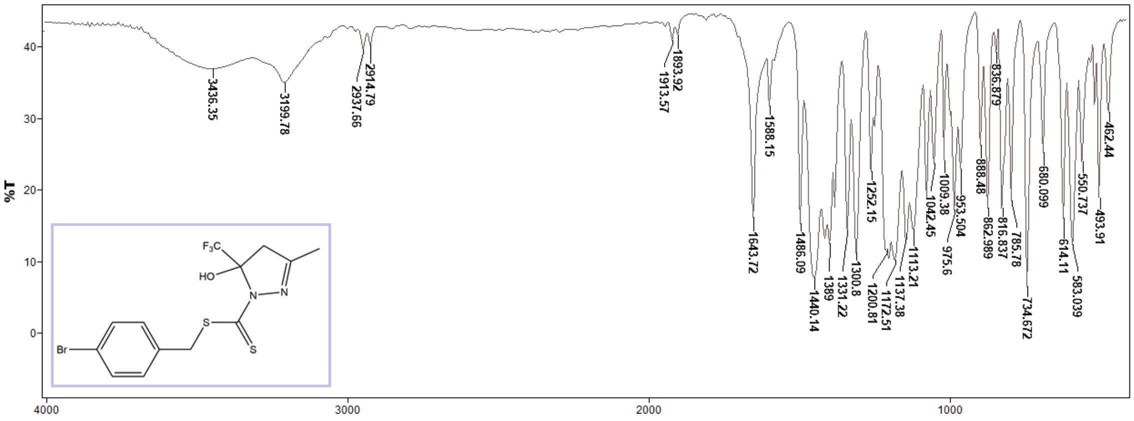

Figure S11. IR spectra of H_2L^1.

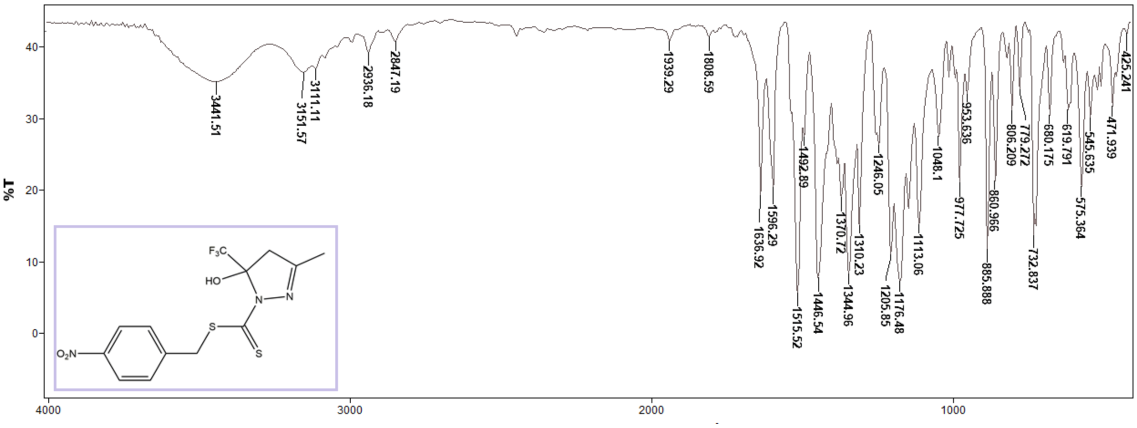

Figure S12. IR spectra of H_2L^2.

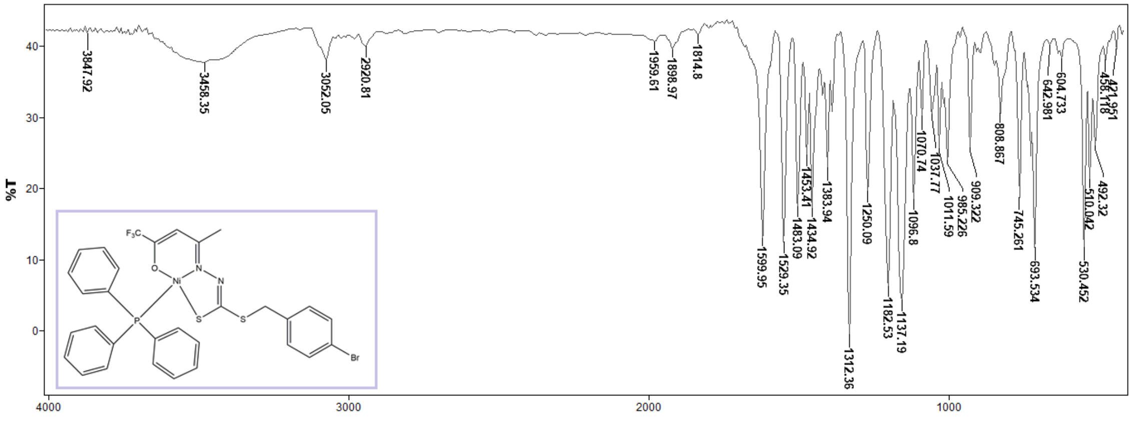

Figure S13. IR spectra of compound (1).

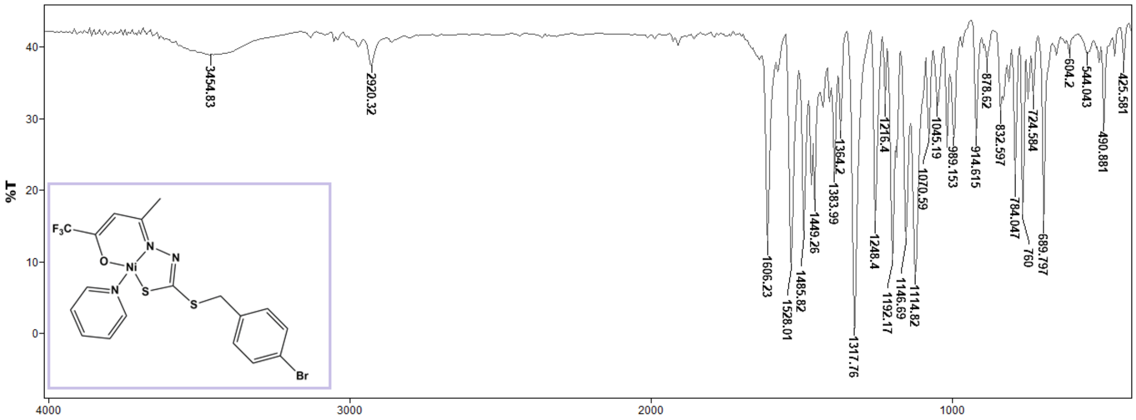

Figure S14. IR spectra of compound (2).

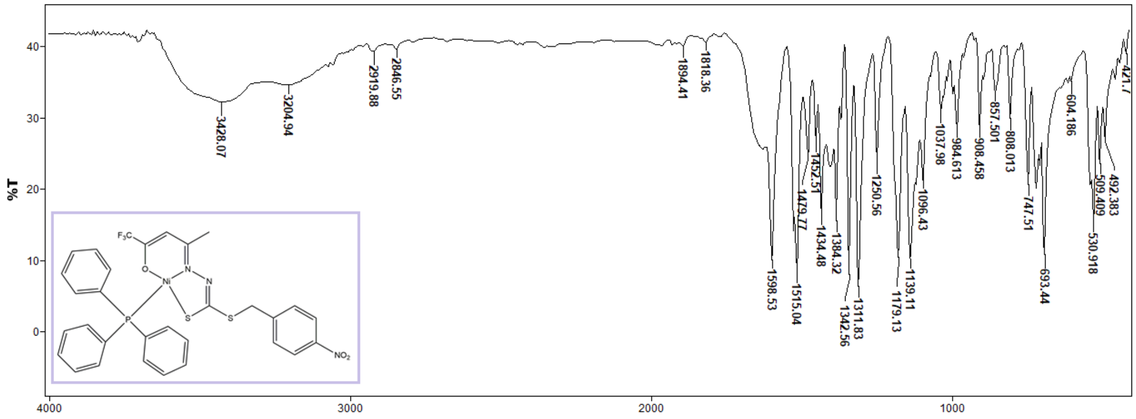

Figure S15. IR spectra of compound (3).

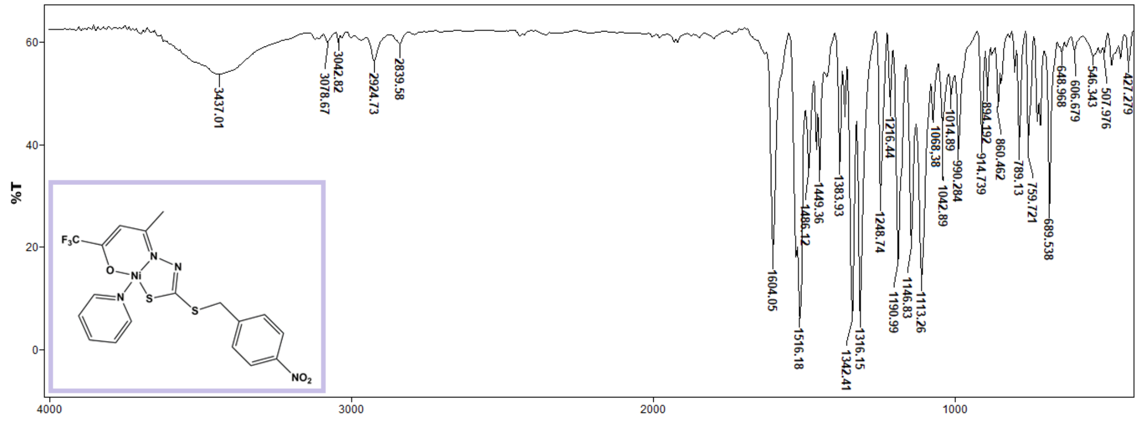

Figure S16. IR spectra of compound (4).

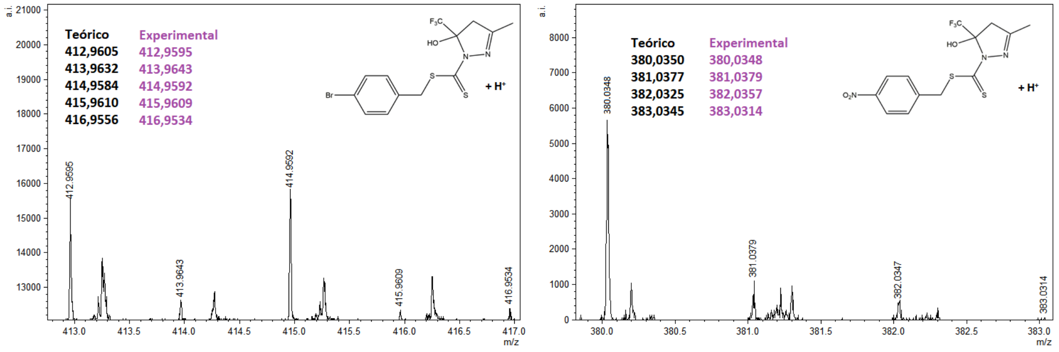

Figure S17. ESI(+)-MS of H_2L^1 and H_2L^2.

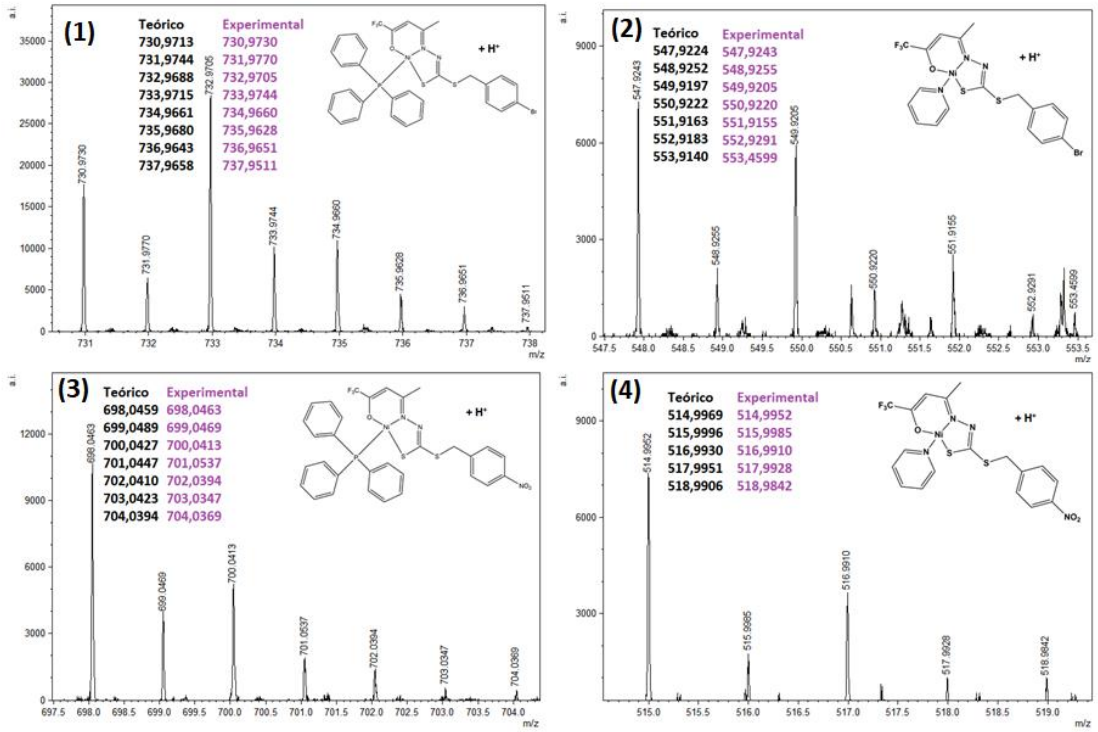

Figure S18. ESI(+)-MS of (1)-(4).

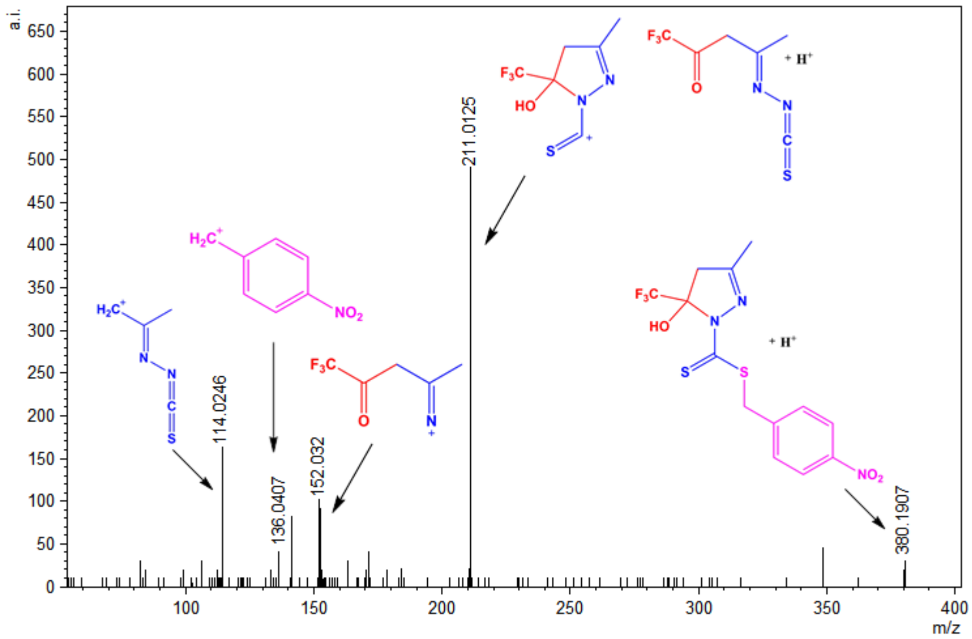

Figure S19. ESI(+)-MSMS of H_2L^2.

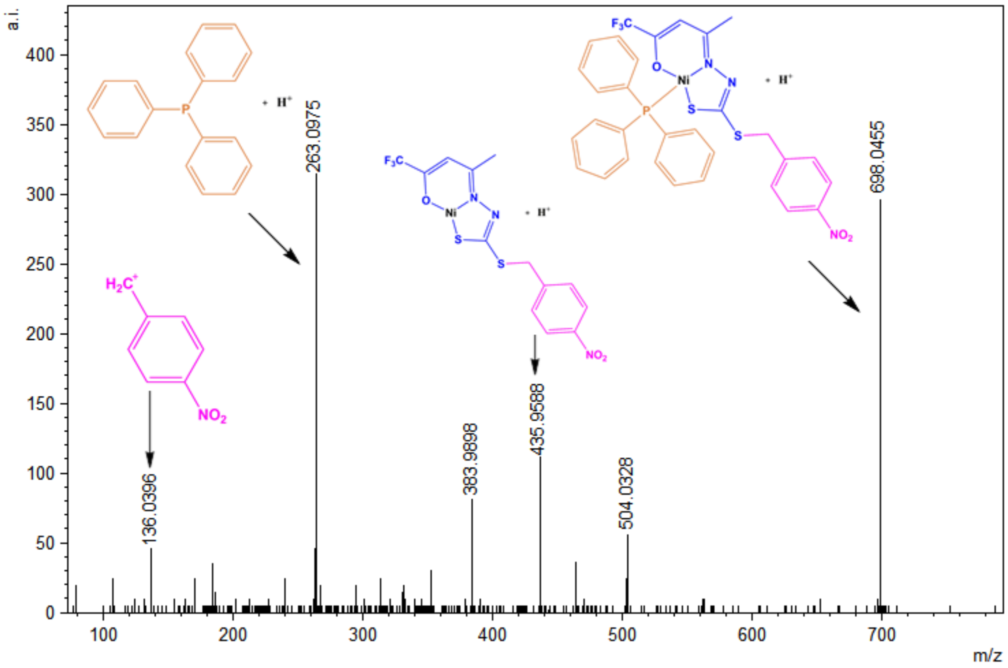

Figure S20. ESI(+)-MSMS of (3)

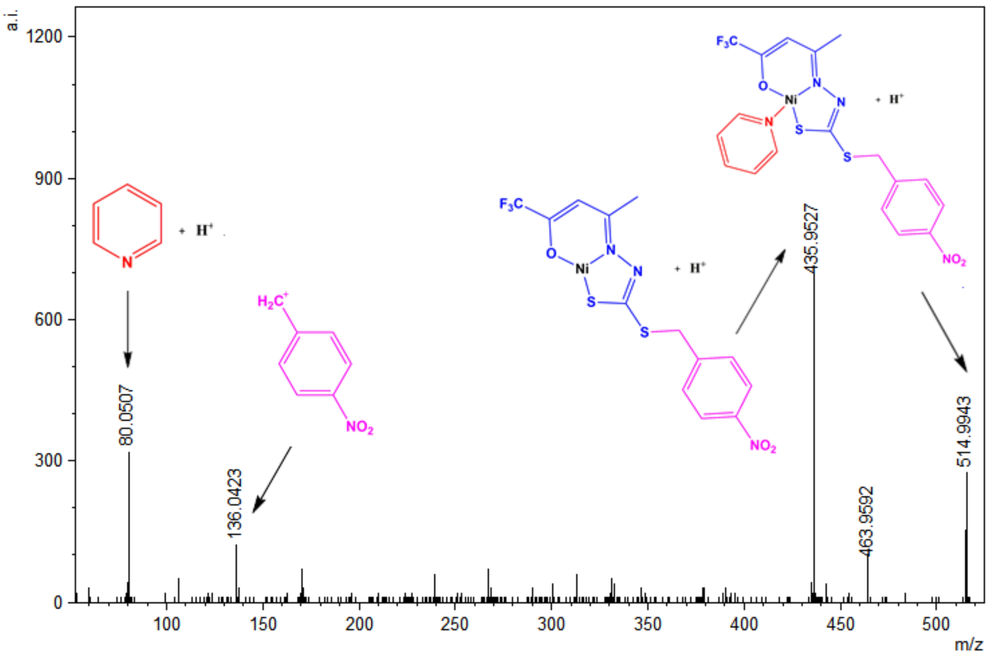

Figure S21. ESI(+)-MSMS of (4)

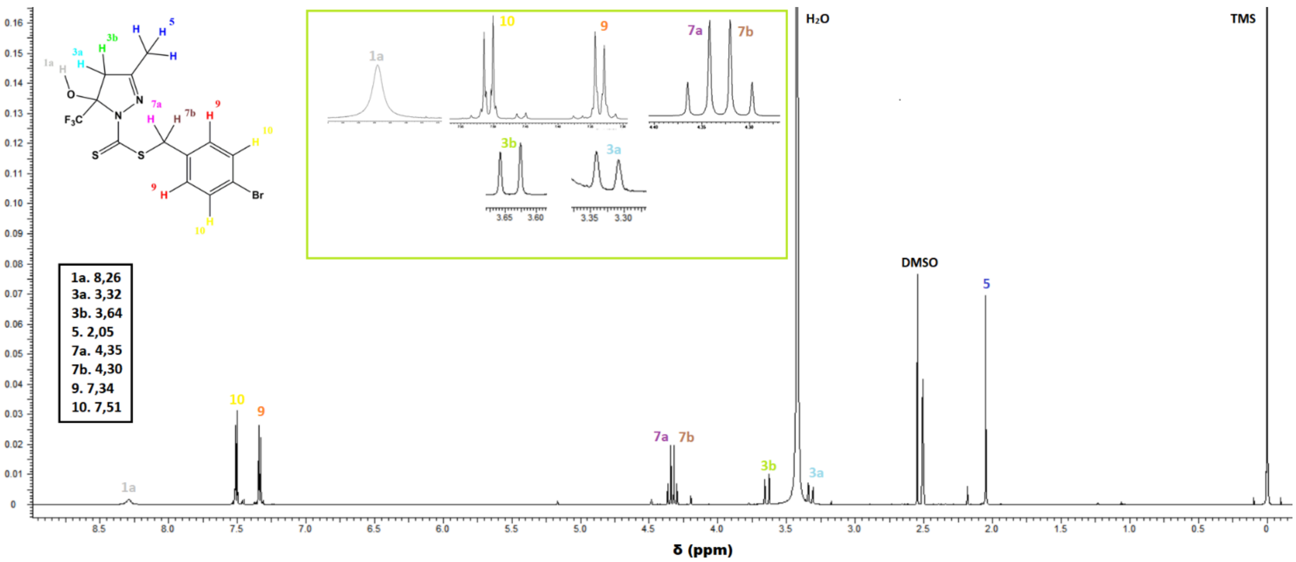

Figure S22. ^{1}H-NMR spectra of compound H_2L^1.

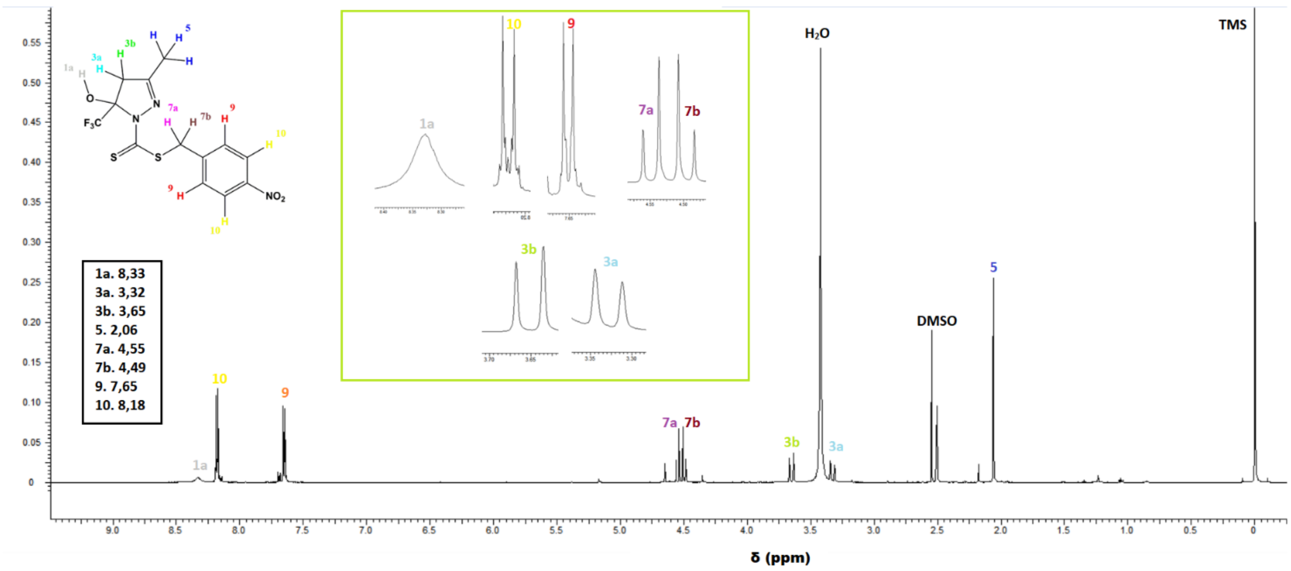

Figure S23. ^{1}H-NMR spectra of compound H_2L^2.

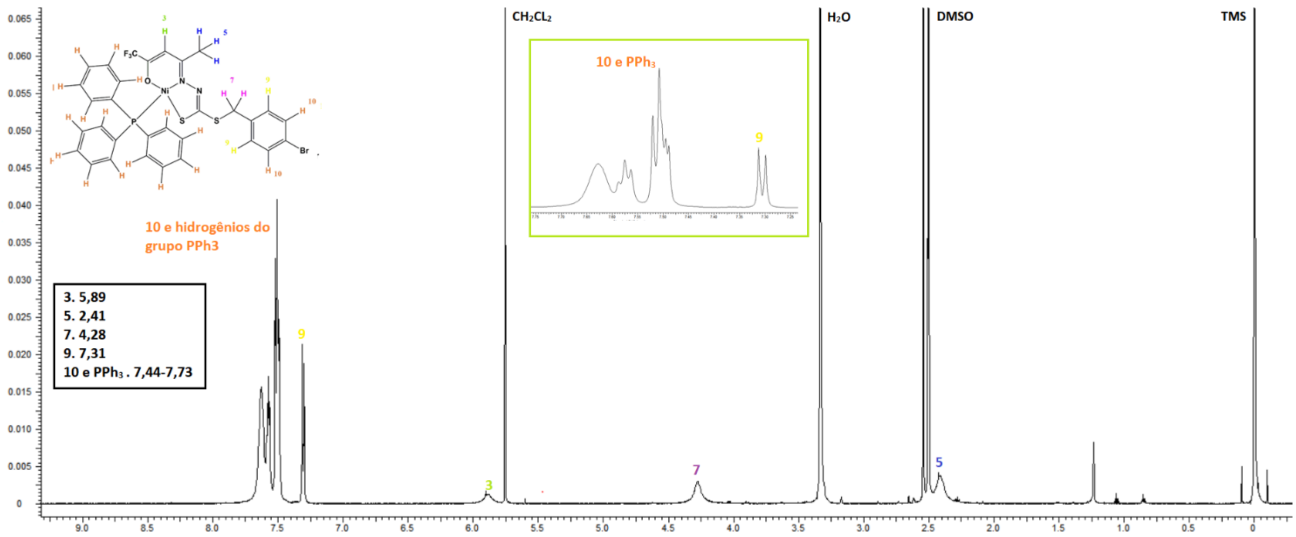

Figure S24. ^{1}H-NMR spectra of compound (1).

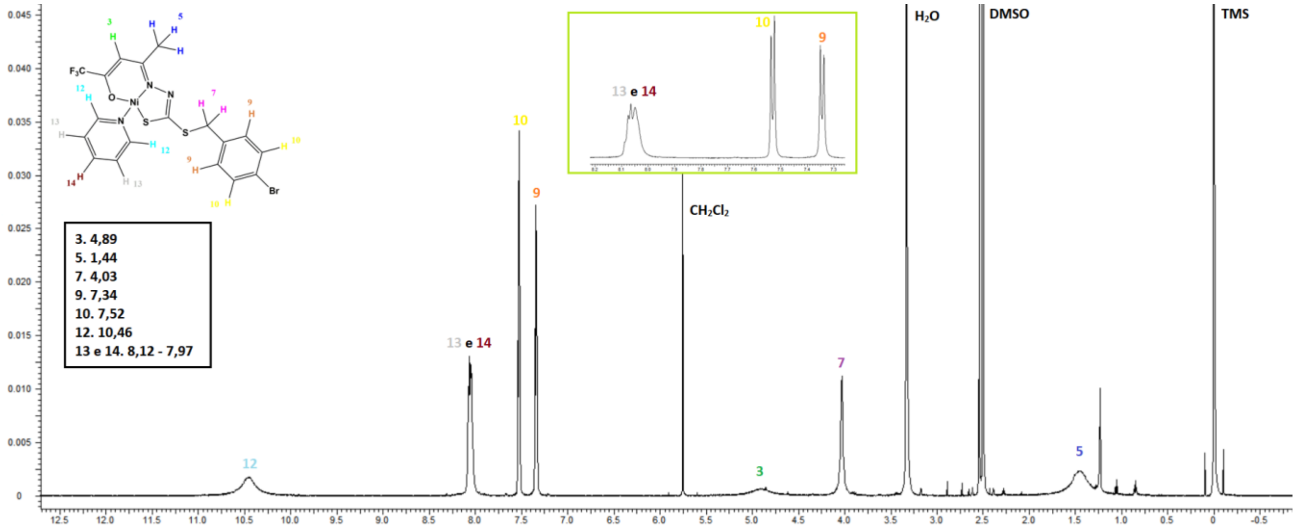

Figure S25. ^{1}H-NMR spectra of compound (2).

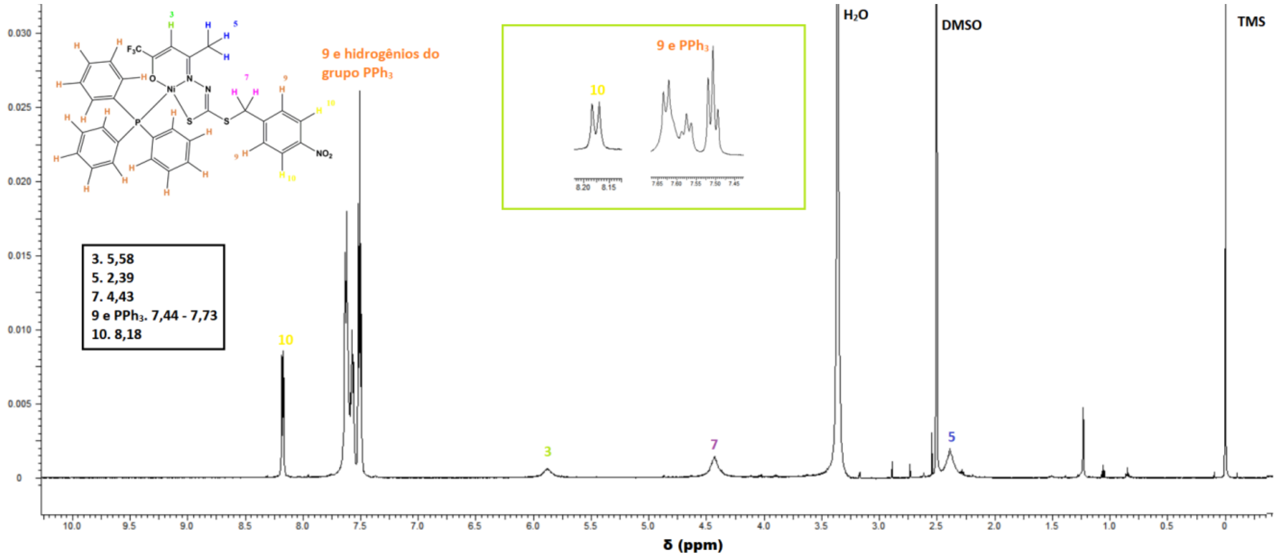

Figure S26. ^{1}H-NMR spectra of compound (3).

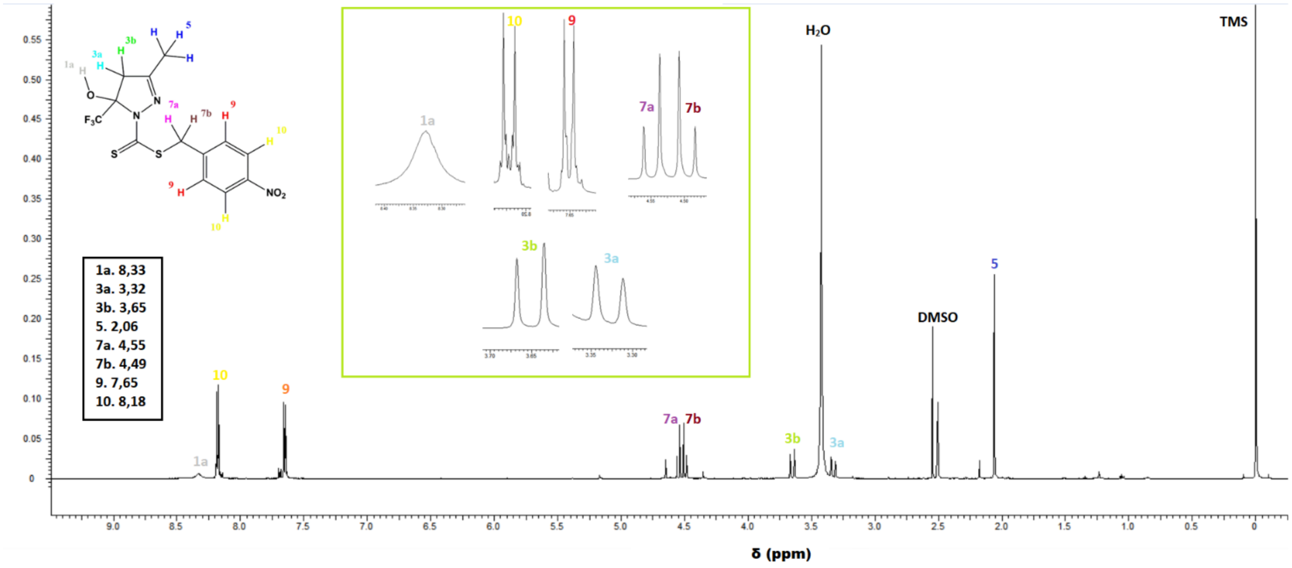

Figure S27. ^{1}H-NMR spectra of compound (4).

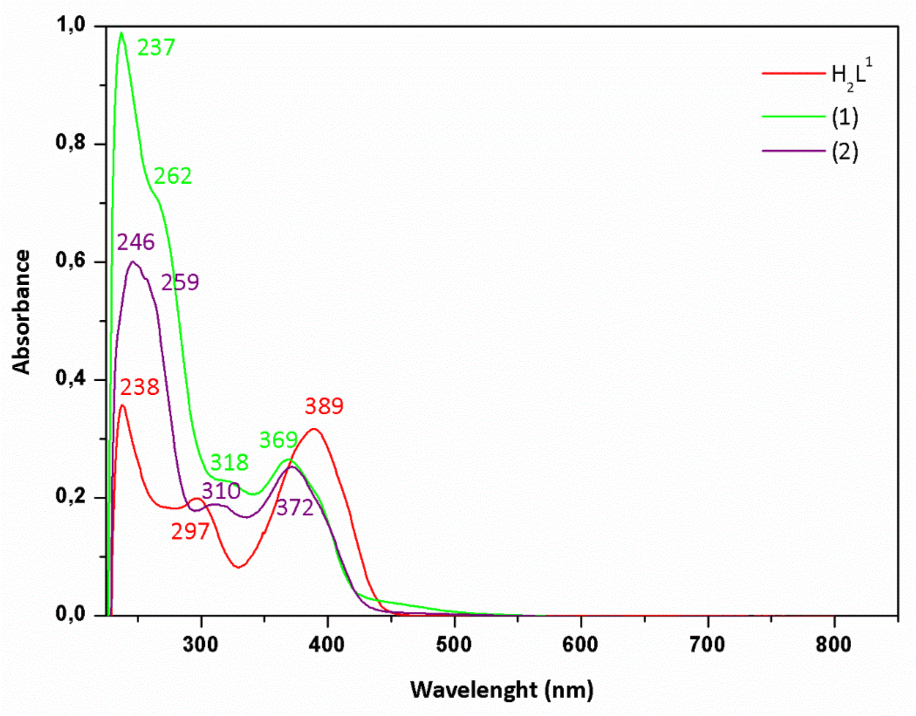

Figure S28. UV-vis spectra of compound H_2L^1, (1) and (2) in MeOH.

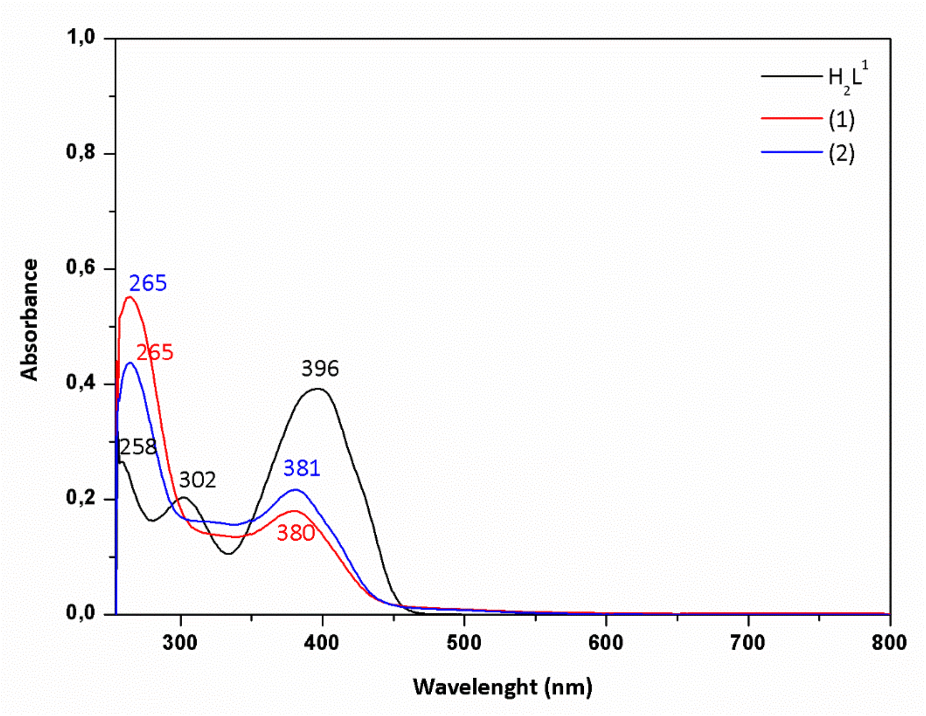

Figure S29. UV-vis spectra of compound H_2L^1, (1) and (2) in DMSO.

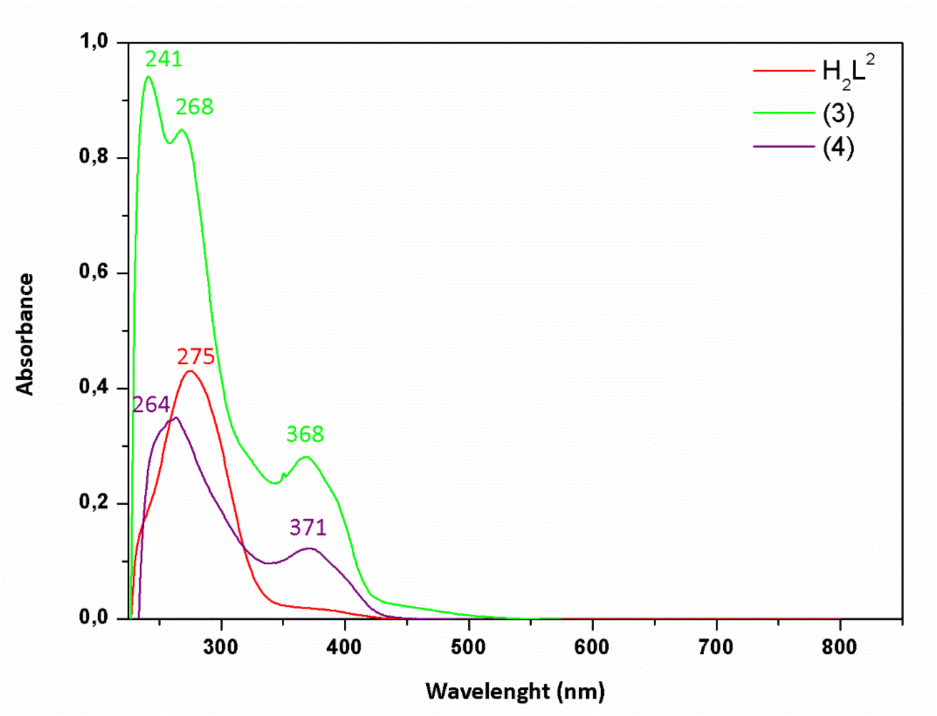

Figure S30. UV-vis spectra of compound H_2L^2, (3) and (4) in MeOH.

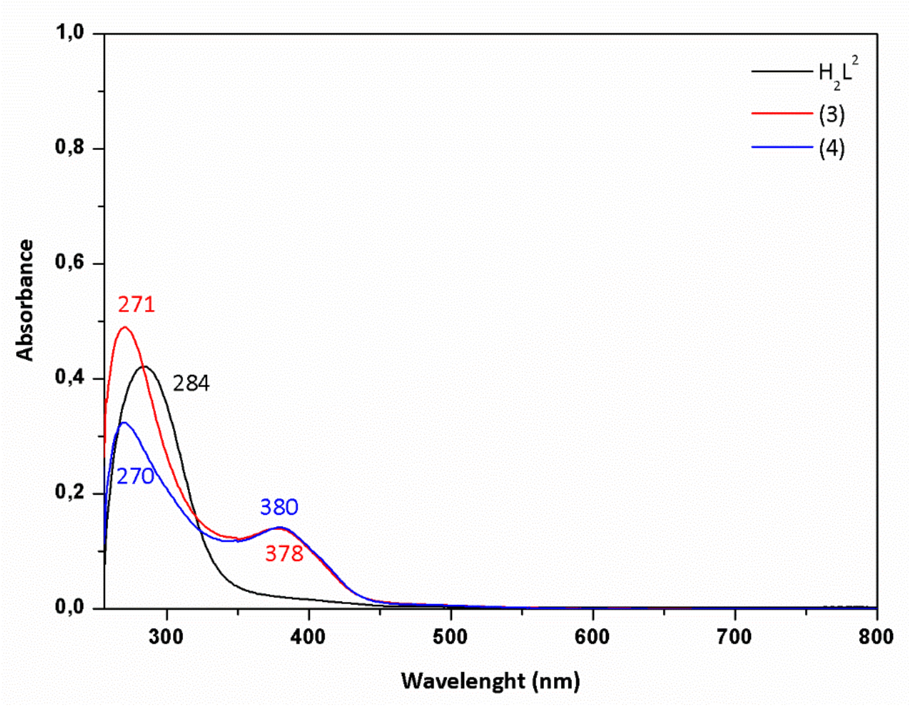

Figure S31. UV-vis spectra of compound H_2L^2, (3) and (4) in DMSO.

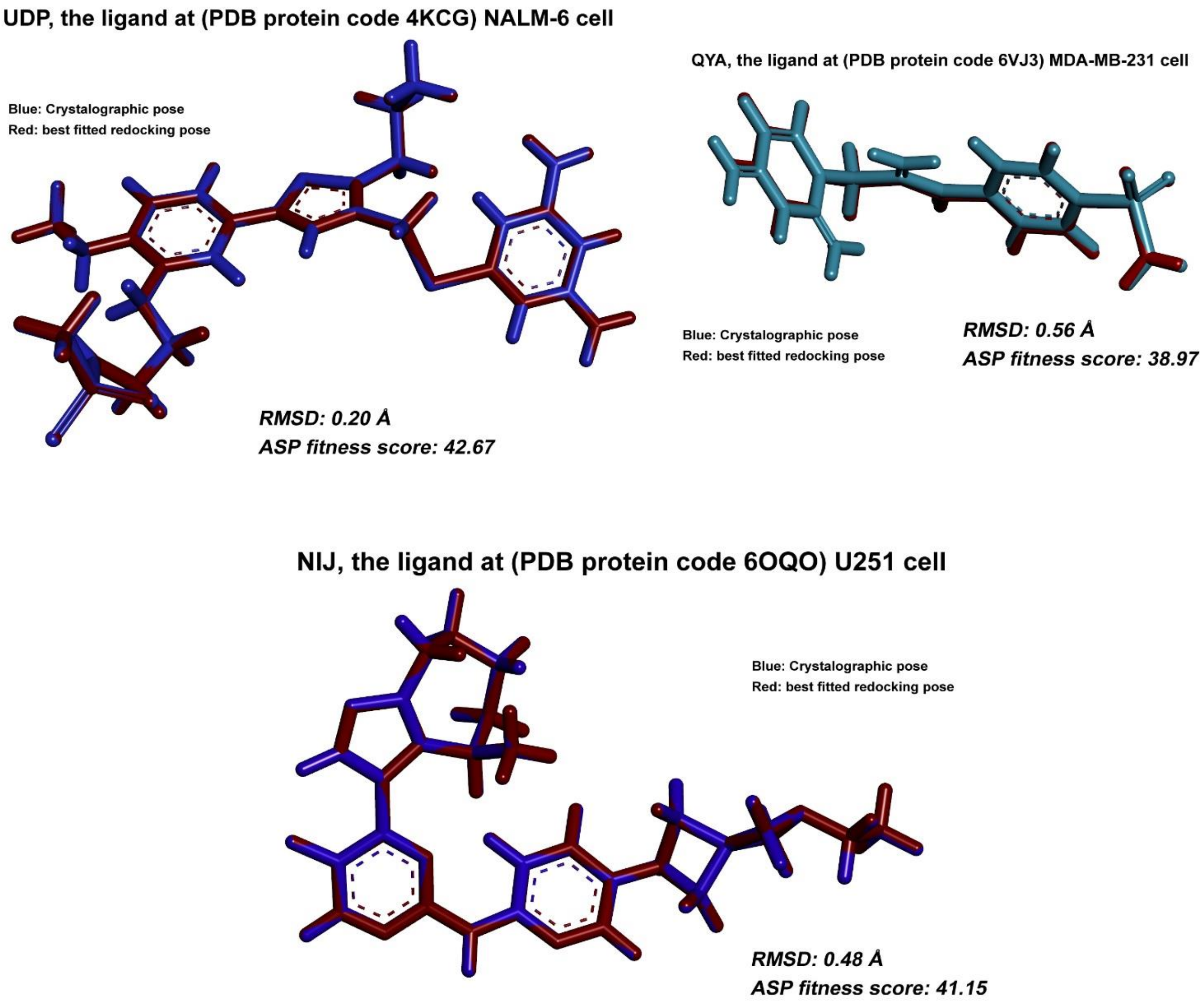

Figure S32. Superposition between the 2D crystallographic structure and 2D redocking (best pose) of the known compounds UDP (Inhibitor of the NALM-6 cell), QYA (inhibitor of MDA-MB-231 cell), and NIJ (Inhibitor of U251 cell) obtained during the validation of docking protocol. The Figure is also shown the values of the RMSD and the ASP fitness score.

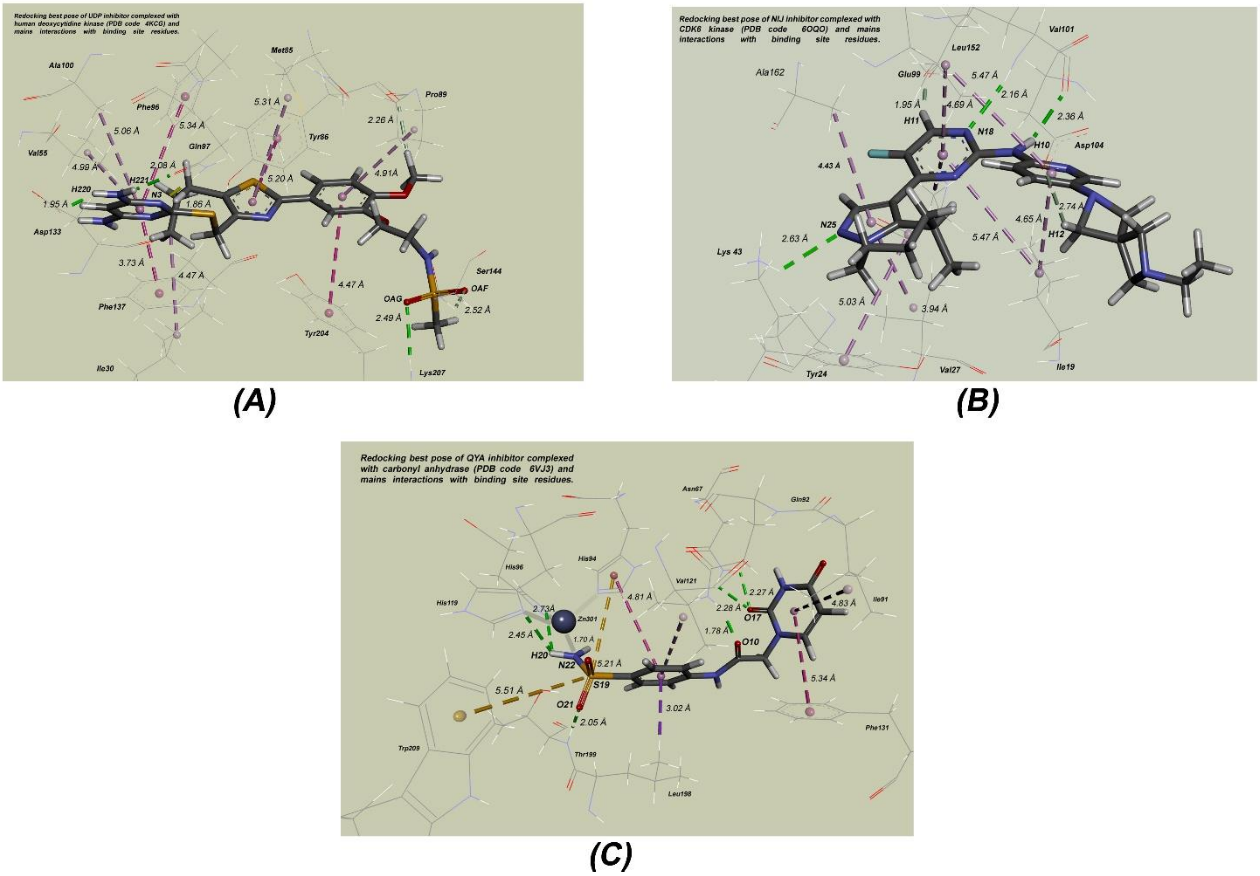

Figure S33. Main interactions and respective distances between the residues of the active site and known inhibitors. For (A) interactions between the UDP inhibitor and the human deoxycytidine kinase active site residues; (B) main interactions between the NIJ classical inhibitor and the CDK6 kinase binding site residues; (C) interactions between the QYA inhibitor and the human carbonyl anhydrase residues.
