# Supplementary material for: Dithiocarbazate ligands and their Ni(II) complexes with potential biological activity: Structural, antitumor and molecular docking study
Source: Front Mol Biosci. 2023 Mar 7;10:1146820. doi: 10.3389/fmolb.2023.1146820 (PMC10034969; doi:10.3389/fmolb.2023.1146820)

checkCIF/PLATON report

Structure factors have been supplied for datablock(s) ccg_coc_ni13_0m

THIS REPORT IS FOR GUIDANCE ONLY. IF USED AS PART OF A REVIEW PROCEDURE FOR PUBLICATION, IT SHOULD NOT REPLACE THE EXPERTISE OF AN EXPERIENCED CRYSTALLOGRAPHIC REFEREE.

No syntax errors found. CIF dictionary Interpreting this report

Datablock: ccg_coc_ni13_0m

```
Bond precision:   C-C = 0.0077 A           Wavelength=0.71073

Cell:              a=4.346(3)     b=38.97(3)            c=12.785(9)
                   alpha=90       beta=96.935(19)       gamma=90
Temperature:       273 K

                 Calculated                        Reported
Volume           2150(3)                           2150(3)
Space group      P 21/n                            P 1 21/n 1
Hall group       -P 2yn                            -P 2yn
Moiety formula   C18 H15 Br F3 N3 Ni O S2          C18 H15 Br F3 N3 Ni O S2
Sum formula      C18 H15 Br F3 N3 Ni O S2          C18 H15 Br F3 N3 Ni O S2
Mr               549.04                            549.07
Dx,g cm-3        1.696                             1.697
Z                4                                 4
Mu (mm-1)        2.995                             2.995
F000             1096.0                            1096.0
F000'            1097.65
h,k,lmax         5,46,15                           5,46,15
Nref             3916                              3886
Tmin,Tmax        0.700,0.796
Tmin'            0.113

Correction method= Not given

Data completeness= 0.992           Theta(max)= 25.276

R(reflections)= 0.0418( 1646)                         wR2(reflections)=
                                                      0.0843( 3886)
S = 0.753                  Npar= 264
```

The following ALERTS were generated. Each ALERT has the format
test-name_ALERT_alert-type_alert-level.
Click on the hyperlinks for more details of the test.

Alert level C

```
GOODF01_ALERT_2_C  The least squares goodness of fit parameter lies
            outside the range 0.80 <> 2.00
            Goodness of fit given =      0.753
RINTA01_ALERT_3_C  The value of Rint is greater than 0.12
            Rint given   0.171
PLAT026_ALERT_3_C Ratio Observed / Unique Reflections (too) Low ..        42% Check
PLAT057_ALERT_3_C Correction for Absorption Required   RT(exp) ...       1.14 Do !
PLAT234_ALERT_4_C Large Hirshfeld Difference C9       --C10      .       0.16 Ang.
PLAT242_ALERT_2_C Low    'MainMol' Ueq as Compared to Neighbors of        C11 Check
PLAT341_ALERT_3_C Low Bond Precision on  C-C Bonds ..............    0.00773 Ang.
PLAT906_ALERT_3_C Large K Value in the Analysis of Variance ......      5.548 Check
PLAT911_ALERT_3_C Missing FCF Refl Between Thmin & STh/L=    0.600         12 Report
```

Alert level G

```
PLAT020_ALERT_3_G The Value of Rint is Greater Than 0.12 .........      0.171 Report
PLAT063_ALERT_4_G Crystal Size Possibly too Large for Beam Size ..       0.72 mm
PLAT199_ALERT_1_G Reported _cell_measurement_temperature ..... (K)        273 Check
PLAT200_ALERT_1_G Reported   _diffrn_ambient_temperature ..... (K)        273 Check
PLAT242_ALERT_2_G Low    'MainMol' Ueq as Compared to Neighbors of         C1 Check
PLAT794_ALERT_5_G Tentative Bond Valency for Ni1       (III)     .       2.77 Info
PLAT912_ALERT_4_G Missing # of FCF Reflections Above STh/L=  0.600         18 Note
PLAT978_ALERT_2_G Number C-C Bonds with Positive Residual Density.          0 Info
```

0 **ALERT level A** = Most likely a serious problem - resolve or explain
0 **ALERT level B** = A potentially serious problem, consider carefully
9 **ALERT level C** = Check. Ensure it is not caused by an omission or oversight
8 **ALERT level G** = General information/check it is not something unexpected

2 ALERT type 1 CIF construction/syntax error, inconsistent or missing data
4 ALERT type 2 Indicator that the structure model may be wrong or deficient
7 ALERT type 3 Indicator that the structure quality may be low
3 ALERT type 4 Improvement, methodology, query or suggestion
1 ALERT type 5 Informative message, check

It is advisable to attempt to resolve as many as possible of the alerts in all categories. Often the minor alerts point to easily fixed oversights, errors and omissions in your CIF or refinement strategy, so attention to these fine details can be worthwhile. In order to resolve some of the more serious problems it may be necessary to carry out additional measurements or structure refinements. However, the purpose of your study may justify the reported deviations and the more serious of these should normally be commented upon in the discussion or experimental section of a paper or in the "special_details" fields of the CIF. checkCIF was carefully designed to identify outliers and unusual parameters, but every test has its limitations and alerts that are not important in a particular case may appear. Conversely, the absence of alerts does not guarantee there are no aspects of the results needing attention. It is up to the individual to critically assess their own results and, if necessary, seek expert advice.

Publication of your CIF in IUCr journals

A basic structural check has been run on your CIF. These basic checks will be run on all CIFs submitted for publication in IUCr journals (*Acta Crystallographica*, *Journal of Applied Crystallography*, *Journal of Synchrotron Radiation*); however, if you intend to submit to *Acta Crystallographica Section C* or *E* or *IUCrData*, you should make sure that full publication checks are run on the final version of your CIF prior to submission.

Publication of your CIF in other journals

Please refer to the *Notes for Authors* of the relevant journal for any special instructions relating to CIF submission.

PLATON version of 28/11/2022; check.def file version of 28/11/2022

Datablock ccg_coc_ni13_0m - ellipsoid plot

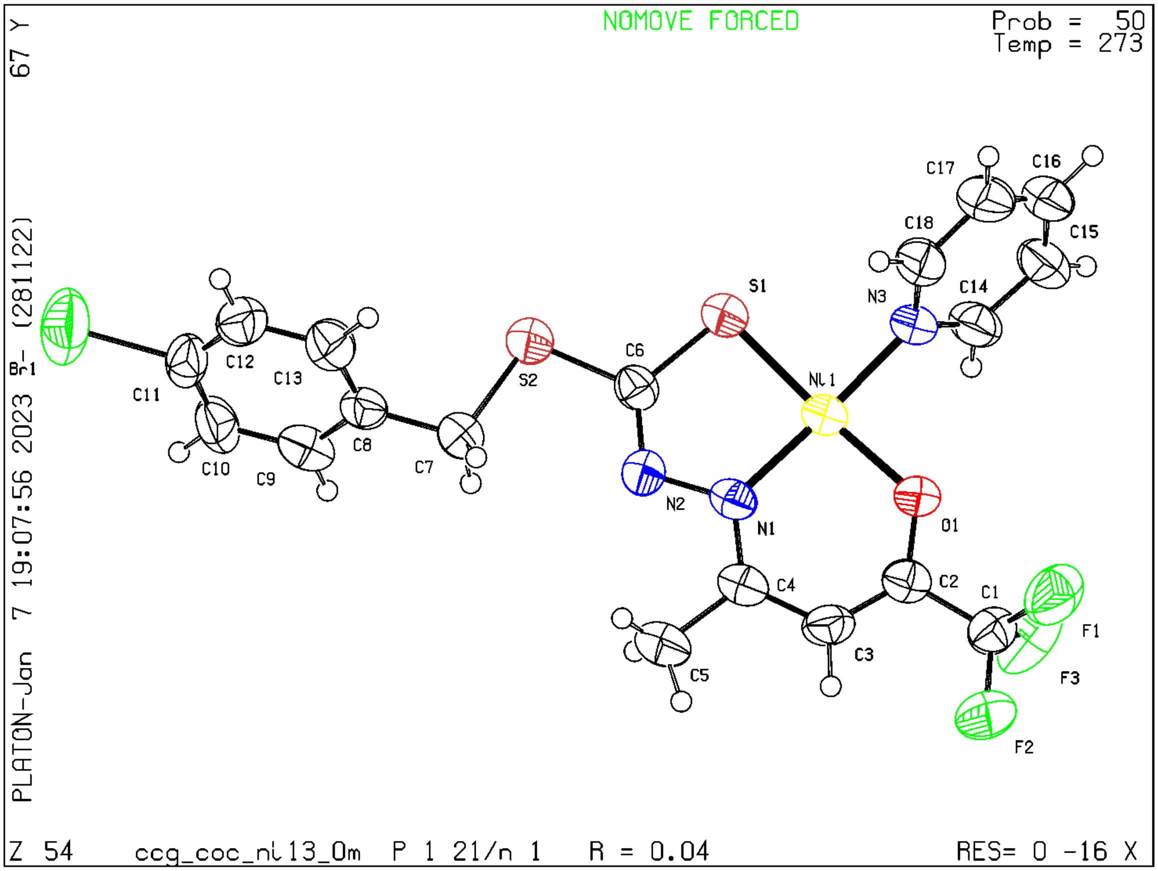

Supplement: Supplementary file 5 [file DataSheet3.PDF]
